# Stereoretentive Nucleophilic Aromatic Substitution Accesses Atropisomeric 4‐Amino‐*N*‐Arylquinolinium Salts

**DOI:** 10.1002/chem.71266

**Published:** 2026-06-20

**Authors:** Darren Holmes, Nia Foster, Saima Ansari, Paul Kavanagh, Paul Dingwall, Peter C. Knipe

**Affiliations:** ^1^ School of Chemistry and Chemical Engineering Queen's University Belfast Belfast UK; ^2^ Institute for Materials Discovery University College London London UK

**Keywords:** asymmetric catalysis, atropisomerism, nucleophilic aromatic substitution, quinolinium

## Abstract

We report a novel synthetic route to access *N*‐aryl‐4‐amino‐quinolinium salts via a stereoretentive S_N_Ar‐type reaction of amines with atropisomeric *N*‐arylquinolin‐4‐ones, by in situ formation of the corresponding chloroquinolinium chloride salt. Investigation into the properties of the chiral salts was carried out using x‐ray crystallography, DFT analysis, cyclic voltammetry and UV–vis spectroscopy. They were revealed to possess high rotational barriers (with lifetimes of millennia), new photophysical properties and low oxidation potentials. A neutral axially chiral quinolinimine could be obtained by a simple deprotonation of the quinolinium salt.

## Graphical abstract

Quinolin‐4‐ones were converted to chloroquinolinium salts, allowing formation of axially chiral aminoquinolinium salts via an enantioretentive S_N_Ar reaction with a variety of amine nucleophiles. The salts have a high rotational barrier, with a racemization half‐life of ∼1000s of years. Their solid‐state structures, and structure‐activity relationships in their photo‐ and electrochemistry were examined.

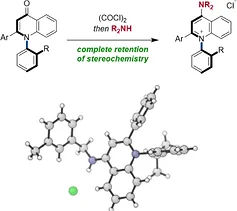

## 1 Introduction

Restricted rotation around a single bond—often a consequence of bulky *ortho* substituents in biaryl systems—can lead to distinct stereoisomers known as atropisomers, which exhibit axial chirality. Molecules containing an atropisomeric C─C axis have been extensively investigated as chiral catalysts [1], ligands [2, 3], and for their pharmaceutical properties [4]. However, molecules containing an axially chiral C─N bond have only recently gained interest. Naturally‐occurring molecules containing this linkage have been reported, such as marinopyrroles which display antibacterial properties [5] and the naphthylisoquinoline alkaloid family of molecules which demonstrate numerous bioactive properties (Figure 1A) [6]. Pharmaceutical interest in heterobiaryl atropisomerism has seen a significant increase in recent years with a number of reported structures found to have antitumor properties, including erastin [7], NCATS‐SM5637 [8], and, most notably, the first‐in‐class KRAS inhibitor sotorasib developed by Amgen [9]. Additional examples include methaqualone, a potent sedative and recreational drug and *N*‐aryl pyridone PH‐797804 which has shown anti‐inflammatory effects for lung conditions such as COVID‐19 and COPD [10, 11].

**FIGURE 1:**
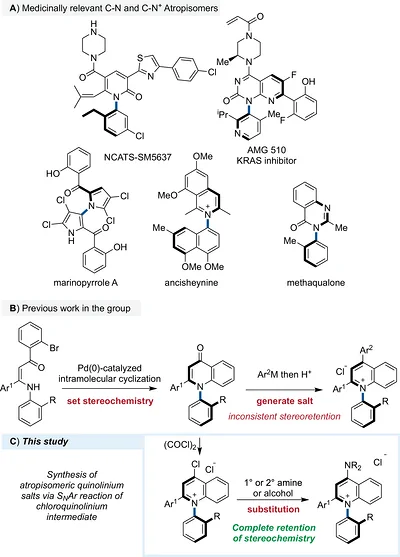
(A) Medicinally relevant C─N atropisomeric compounds; (B) previous approach to atropisomeric quinolinium salts via organometallic addition into quinolin‐4‐ones [12]; (C) proposed strategy to generate enantio‐enriched atropisomeric quionolinium salts via S_N_Ar reaction of a chloroquinolinium intermediate.

Synthetic approaches to molecules containing the C─N chiral axis are increasingly common [13, 14, 15, 16, 17, 18, 19, 20, 21, 22, 23, 24]. By contrast, to date there are few routes reported to access atropisomeric quaternary C─N^+^ biaryl salts, whose chemical properties remain minimally explored, though commercial antimicrobial (dequalinium) and antiparasitic (pyrvinium) therapeutics contain quaternized quinolinium cores. Contemporary methods exist to access racemic and achiral molecules of this family, including well‐established chemistry utilizing methods such as the Zincke reaction **[** 25 **]** and pyrylium condensation **[** 26 **]**, *N*‐Arylation strategies have emerged using hypervalent iodine (III) **[** 27, 28 **]**, Cu/Fe catalysis **[** 29 **]**, addition‐elimination to acridones **[** 30, 31 **]**, an electrochemically driven anodic oxidation approach **[** 32 **]**, and by copper‐catalyzed Chan–Lam coupling of pyridines with arylboronic acids [33]. Work published by Sparr et al. reporting the synthesis of atropisomeric *N*‐aryl acridinium catalysts **[** 34, 35 **]** inspired previous research in our group to synthesize *para‐*substituted axially chiral quinolinium salts from atropisomeric 4‐quinolone precursors while maintaining the chiral axis **[** 12 **]**. Synthesis of the quinolinium salt was achieved by two methods: addition of PhLi to the neutral quinonlin‐4‐ones or pre‐treatment of the neutral species with oxalyl chloride and subsequent addition of PhMgBr to yield the 4‐phenyl‐quinolinium salts with variable levels of stereoretention. The use of aryl organometallic reagents presented a difficulty in that a competing single electron reduction pathway led to product racemization in some cases. Here, we seek to exploit the reactivity of *N*‐aryl 4‐chloroquinolinium species toward S_N_Ar chemistry to achieve *para*‐substituted axially chiral *N*‐aryl quinolinium salts with full stereoretention, and avoiding the use of harsh organometallic reagents.

## 2 Results and Discussion

### 2.1 Synthesis

Initially a small group of enaminones was synthesized according to a two‐step procedure: Sonogashira coupling of 2‐bromobenzoyl chloride and phenylacetylene to give the corresponding diphenyl ynone, and a subsequent hydroamination with various *ortho*‐substituted anilines to give the desired enaminone substrate **1** (Scheme 1).

**SCHEME 1:**
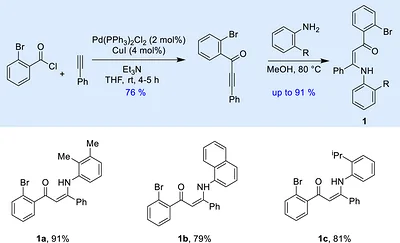
Two‐step synthesis of enaminones.

As shown in previous work published in the group, asymmetric palladium‐catalyzed intramolecular cyclization of enaminones was effective to obtain axially chiral *N*‐aryl‐4‐quinolones **[** 12 **]**. Building on the foundational work by Kitagawa **[** 36 **]**, Liu et al. reported very effective conditions to achieve these structures in good to excellent yield and *ee* **[** 37 **]**. With the enaminone substrates in hand, we carried out initial intramolecular Buchwald–Hartwig coupling reactions according to the procedure described by Liu et al. (Table 1).

**TABLE 1:** Re‐optimization of atropselective Buchwald–Hartwig cyclization of enaminone **1a**.

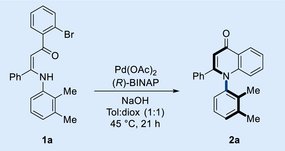				
Entry	Cat. loading (mol%)	Ligand loading (mol%)	Yield (%)^a^	*ee* (%)^b^
1^c^	5	10	50	9
2^c^	10	20	77	92
3^d^	10	20	30	55
4^e^	10	20	75	80

^a^ Yields are for isolated material.

^b^ *ee* determined by HPLC on a chiral stationary phase.

^c^ conducted on 0.1 mmol scale of **1a**.

^d^ conducted on 0.5 mmol scale of **1a**.

^e^ conducted by running 5 x 0.1 mmol scale reactions in parallel. For full experimental details please refer to the Supporting Information.

Initial conditions directly according to this procedure yielded the atropisomeric *N*‐aryl‐quinolin‐4‐one product in 50% yield, with only 9% *ee*. Increasing the catalyst and ligand loadings to 10 and 20 mol%, respectively, gave the desired chiral product in 77% yield and 92% *ee* (entry 2). However, despite various alterations of reaction conditions, scale‐up reactions could not reproduce the desired *ee* values found in the small‐scale reactions (entry 3). *In lieu* of a one‐pot scale‐up, larger amounts of chiral material were obtained by a “scale‐out” process, combining individual 0.1 mmol reactions (entry 4, see Supporting Information for details). We have previously highlighted the sensitivity of this reaction toward experimental factors: in an earlier study, use of the same (*R*)‐ligand and alternative palladium source was found to lead to a bias towards the opposite enantiomer of product **[** 12 **]**. This was attributed to the formation of BINAP monoxide when Pd(II) sources were used, which itself serves as a competent ligand. The reaction is therefore sensitive to the presence of any oxidants including adventitious oxygen and peroxide species within ethereal solvents. Though not available in our laboratory, it is likely that greater reproducibility could be achieved using glovebox protocols.

With the optimized conditions in hand, we synthesized a small library of quinolin‐4‐ones (Scheme 2) with various *ortho*‐substituted anilines. Quinolin‐4‐ones **2a** and **2c** were obtained in moderate to good yield (77% and 60%, respectively) and very good enantioselectivity (92% and 91%, respectively). *N*‐2‐Isopropylphenylquinolone **2b** showed much reduced yield and enantioselectivity under these conditions (52% yield, 85% *ee*) but had not been previously synthesized via this method. Though lower *ee* values were obtained on scaling‐out the reactions, selectivities were sufficiently high to be used in the subsequent S_N_Ar study.

**SCHEME 2:**
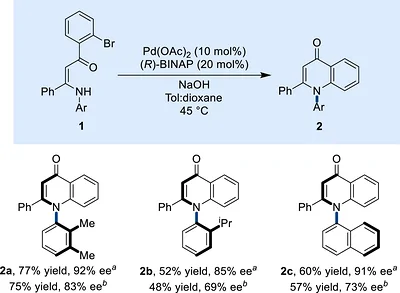
Enantioselective synthesis of quinolin‐2‐ones **2**. [a] Reaction conducted on 0.1 mmol scale; [b] conducted by running 5 x 0.1 mmol scale reactions in parallel. The absolute configuration of the products was determined by comparison with prior literature reports [37].

Previous efforts to isolate or confirm formation of the chloroquinolinium intermediate **3a** proved difficult [12]. Therefore, conditions were re‐optimized to form **3a** (Scheme 3 and see Supporting Information, section 2.3.1), with LC‐MS analysis of the resulting mixture showing the formation of chloroquinolinium chloride **3a** with a conversion of 90% within 1 h. When the reaction was carried out in the presence of DMF (2‐3 drops), similar to conditions reported by Tinnermann et al. [38], LC‐MS analysis revealed the appearance of a new species in the mixture with a similar retention time value to **3a** but with a mass of 353. We determined that this was the 4‐amino substituted product **4**, formed via an S_N_Ar‐type reaction of the chloroquinolinium intermediate **3a** with dimethyl amine formed by the partial decomposition of the DMF additive (Scheme 3).

**SCHEME 3:**
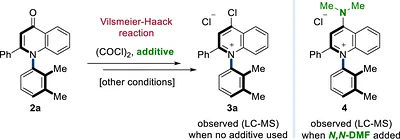
Chloroquinolinium chloride salt synthesis under Vilsmeier–Haack conditions, and unexpected formation of dimethylaminoquinolinium salt **4**.

The S_N_Ar substitution of amines into 4‐chloroquinolinium salts has previously been described in achiral systems **[** 39 **]**. Curious to know whether the intermediate **3** could undergo similar S_N_Ar‐type reactions, and to explore the effect on stereochemistry, we subjected **3** (formed in situ from the corresponding quinolin‐4‐one **2**) to similar conditions using pyrrolidine as a nucleophilic nitrogen source via a one‐pot procedure under mild conditions (Scheme 4). We were delighted to see that 4‐amino‐quinolinium salt **5a** was obtained in 69% yield with complete stereoretention. A library of salts was then synthesized using a variety of primary and secondary amines, with the stereoretention measured for each salt. HPLC resolution of the salts required extensive screening of chiral columns, and in some cases (**5g**–**5i**, **5m**, **5n**) was ultimately unsuccessful. Where enantioretention values exceed 100%, we assume this is due to error in the measurement of *ee* due to imperfect resolution or co‐elution of trace confounding impurities [40]. Enantio‐enrichment in related molecules has been observed during silica gel chromatography by a process known as self‐disproportionation of enantiomers [41, 42]. We do not believe this process to be in operation here, with no observation of any difference in *ee* between column fractions. The reaction generally gave good yields. The pyrrolidine‐derived molecules, **5a–c** were formed in moderate yields (69%–73%), similar to the more hindered diisopropylamine‐derived **5g–i** and dibenzylamine‐derived salts **5j–l**. In comparison, the piperidine‐derived quinoliniums **5d–f** and primary amine‐derived **5m–r** were formed in excellent yields (86%–99%). No significant side‐products were observed, with lower yields generally accompanied by formation of the corresponding quinolin‐2‐one **2**, likely due to hydrolysis by adventitious water. Recovered quinolin‐2‐ones had identical *ee* to the starting materials. Previous work in the group suggested the ability of the sp^3^‐C─N bond formed in the quinoline intermediate to undergo pyramidalization, allowing for facile rotation of the C─N axis and subsequent racemization of the quinolinium product (see Figure 2 below). In this work we observe virtually no racemization for any of the quinolinium products measured.

**SCHEME 4:**
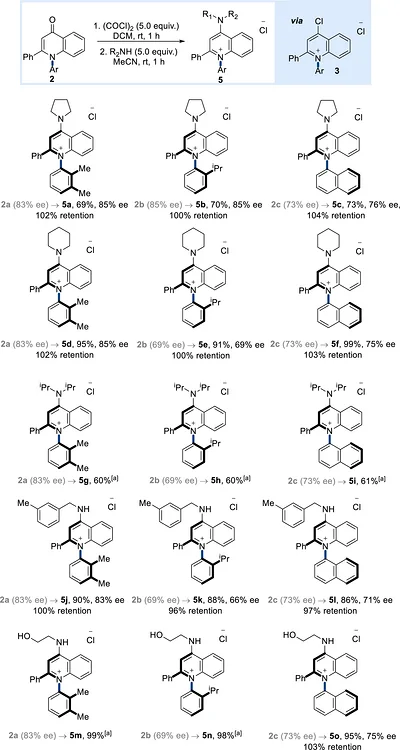
Scope of synthesis of 4‐aminoquinolinium salts **5** by stereoretentive S_N_Ar reaction. Yields are for isolated material. *ee* determined by HPLC on a chiral stationary phase. [a] *ee* of product could not be determined since product peaks did not fully resolve on any available chiral stationary phase. % retention calculated by $\frac{ee_{\mathrm{prod}}}{ee_{\mathrm{SM}}}\times 100$.

**FIGURE 2:**
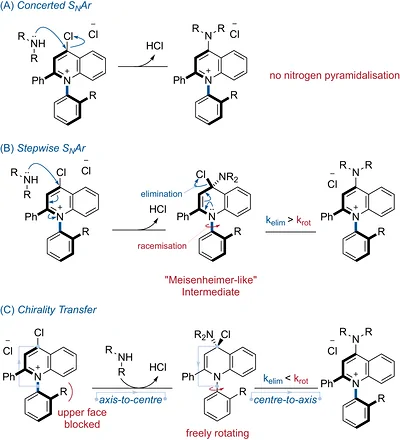
Possible mechanistic explanations of stereoretentive behavior.

In **5j–5o**, the use of primary amine nucleophiles means that the product quinolinium may exist in equilibrium with a neutral exocyclic imine derivative. Lambert has previously shown that 2π aromatic cyclopropenium cations can be deprotonated to generate the corresponding cyclopropenimines, which themselves can act as competent Bronsted bases for asymmetric catalysis [43]. To test whether analogous imines could be generated here, **5l** was treated with 1 m sodium hydroxide solution, which quantitatively converted it to the imine **6** (Scheme 5), with no loss in stereochemical integrity (see Supporting Information).

**SCHEME 5:**
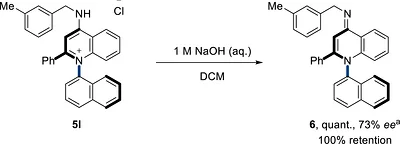
Synthesis of neutral exocyclic imine **6** from quinolinium salt **5l**. [a] The *ee* of **6** was determined by HPLC on a chiral stationary phase following its acidic hydrolysis to the corresponding quinolin‐4‐one **2c**.

### 2.2 Mechanistic Discussion

The exceptional stereoretention observed (which contrasts with our related work utilizing organometallic nucleophiles [12]) bears scrutiny. In those cases, we hypothesized that in the Meisenheimer‐like intermediate (or the product of single electron transfer between the nucleophile and quinolinium salt) there was increased pyramidalization of the axial nitrogen, and a commensurate reduction in rotational barrier leading to partial or complete racemization. In the current case, several explanations for the stereoretentive behavior can be offered. (1) The reaction may proceed via a concerted S_N_Ar process (Figure 2A). Jacobsen et al. reported that in cases where S_N_Ar reactions are carried out in heteroaromatic rings with good leaving groups, the reactions favor a concerted mechanism over the formation of the Meisenheimer intermediate [44]. A lack of Meisenheimer‐like intermediate would explain the lack of racemization observed in these reactions. (2) A stepwise S_N_Ar reaction occurs, but the Meisenheimer‐like intermediate is short‐lived, such that axial bond rotation does not compete kinetically with elimination of chloride (Figure 2B). (3) If axial bond rotation is faster than elimination, the same addition‐elimination reaction may also form the enantio‐enriched product by axis→centre→axis chirality transfer (Figure 2C). However, the distance between the chiral axis and the stereogenic center in the intermediate makes it unlikely this process would proceed with such high stereoinduction in both steps, and the steric congestion around the C─N axis in the Meisenheimer‐like intermediate suggest bond rotation is likely to be slow. We therefore suggest that the explanation for the stereoretentive behavior is either (1) or (2)—that is, the Meisenheimer‐like intermediate either does not form at all, or is sufficiently short‐lived to obviate racemization by bond‐rotation.

### 2.3 Analytical and Spectroscopic Properties

Single crystal racemates of **5k** were grown by vapor diffusion which allowed analysis to be carried out by single‐crystal x‐ray diffraction. Comparison to axially chiral 4‐phenyl‐quinolinium salts reported previously in the group showed a similar axial C‐N bond length. Interestingly, the exocyclic C^6^─N^2^ bond is the shortest C─N bond within the structure, which is most likely due to the significant contribution of the iminium resonance form of this structure (Figure 3). The *N*‐aryl ring is tilted out of plane with the quinolinium and is notably more tilted than previous examples by at least 3° [12]. Greater pyramidalization of the axial nitrogen is also observed (sum of angles around N = 360.0° in the 4‐phenyl salt [12, 45] *vs* 358.8° for **5k**), also consistent with the iminium resonance form being a significant contributor, though crystal packing effects cannot be ruled out as a factor in this difference. Coordination of the chloride counter‐ion was seen to occur with N^2^─H, in contrast with our previous studies where the counter‐ion was coordinated to the electron‐deficient π‐surface of the quinolinium ring. Although diffraction quality crystals of enantioenriched samples could not be obtained, we assign axial chirality to these structures on the basis of the literature procedure used and confirmation that the method is enantioretentive in all measurable examples with no likely pathway for stereoinversion.

**FIGURE 3:**
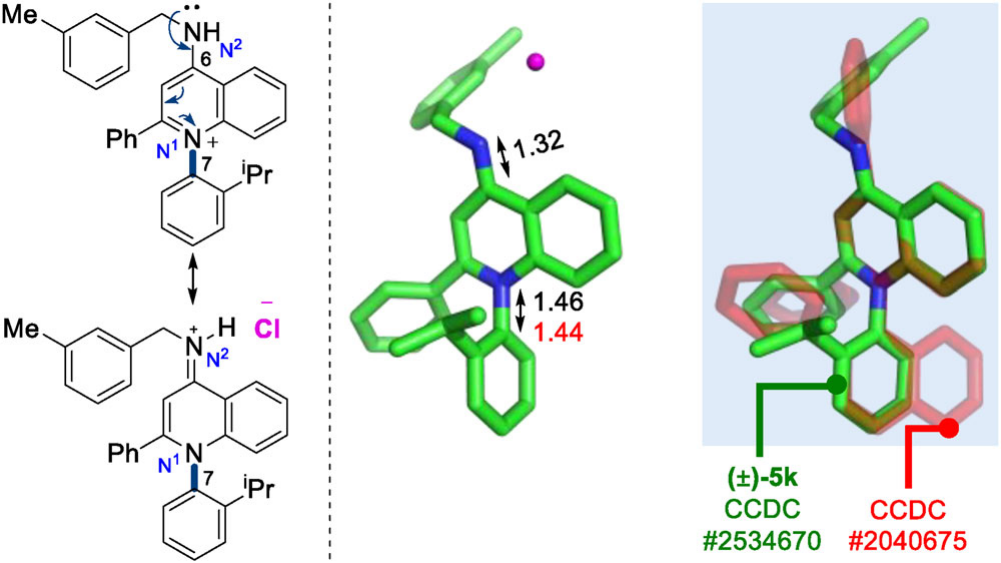
Single crystal x‐ray structure of **(±)‐5k** (CCDC #2534670) [46]. Inset: overlaid with CCDC #2040675. Interatomic distances (in Å) for **5k** are indicated in black, and the corresponding distances for CCDC #2040675 in red.

Given the previously noted difficulty in determining rotational barriers experimentally for these types of structures [12, 47], barriers were obtained using a DFT approach for **5b, 5c,** and **5i** (Figure 4). This analysis revealed that **5b** and **5c** possess the highest barriers to rotation (Δ*G* ^‡^ = 36.4 and 36.0 kcal mol^−1^, respectively). The barrier for **5c** represents a reduction of 3.6 kcal mol^−1^ compared with the analogous 4‐arylated quinolinium salt previously reported [12], consistent with the greater pyramidalization present at the axial nitrogen. Interestingly, the nature of the 4‐amino substituent appears to influence the rotational barrier of the molecule, evident by the reduced kinetic barrier of **5i** (32.2 kcal mol^−1^) [48]. The barrier for the exocyclic imine **6** was significantly reduced compared with the quinolinium salts (26.2 kcal mol^−1^, *t*_1/2_ ∼16 days), consistent with greater pyramidalization of the axial nitrogen. These results suggest that for any future use in asymmetric catalysis the imine should be formed immediately prior to use and used at low temperatures, or the barrier should be raised by introducing additional steric bulk at the chiral axis.

**FIGURE 4:**
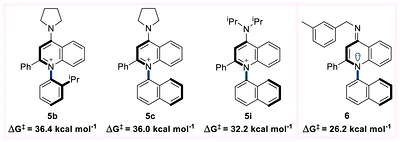
Barriers to rotation for **5b**, **5c**, **5i**, and **6** determined by DFT, modelled in the absence of a counter‐ion. Values calculated at the B3LYP/6‐311+G(d,p)//B3LYP/6‐31G(d,p)/GD3BJ level of theory [49].

Research conducted by Lambert et al. reported the use of 2π aromatic trisaminocyclopropenium (TAC^+^) ion as an electrophotocatalyst via an initial oxidation under electrochemical conditions to form the dication intermediate **[** 50, 51, 52 **]**. A key feature of this TAC^+^ species allowing this catalytic behavior was its accessible oxidation potential (*E*_1/2_ = +0.88 V vs. Fc/Fc^+^; converted from +1.26 V vs. SCE using an offset of 0.38 V) [53]. We conceived that the quinolinium salts synthesized might behave as a 6π aromatic analogue of these systems. Anodic (oxidation) peak potentials (E_pa_) for a number of quinolinium compounds bearing secondary amino groups were determined by cyclic voltammetry (Figure 5). All compounds displayed irreversible oxidation waves, consistent with a rapid follow‐up chemical reaction of the initially formed radical dication. The 4‐piperidinyl compounds **5d**–**f** were found to have values between +0.79 to +0.82 V vs Fc/Fc^+^, slightly lower than the oxidation potential reported for the TAC^+^ ion [50]. Furthermore, the variation in values between these compounds suggests the nature of the *N*‐aryl ring and amine may allow tuning of this redox potential [54]. Pyrrolidinyl‐substituted **5a** was found to be slightly lower again (+0.72 V vs. Fc/Fc^+^) and, curiously, compound **5** **g** was found to have two much lower oxidation peaks (*E*_ox1_ = +0.22 V, *E*_ox2_ = +0.65 V vs. Fc/Fc^+^), possibly a consequence of the bulky amine substituents preventing efficient conjugation of the exocyclic amine with the electron‐deficient π‐system.

**FIGURE 5:**
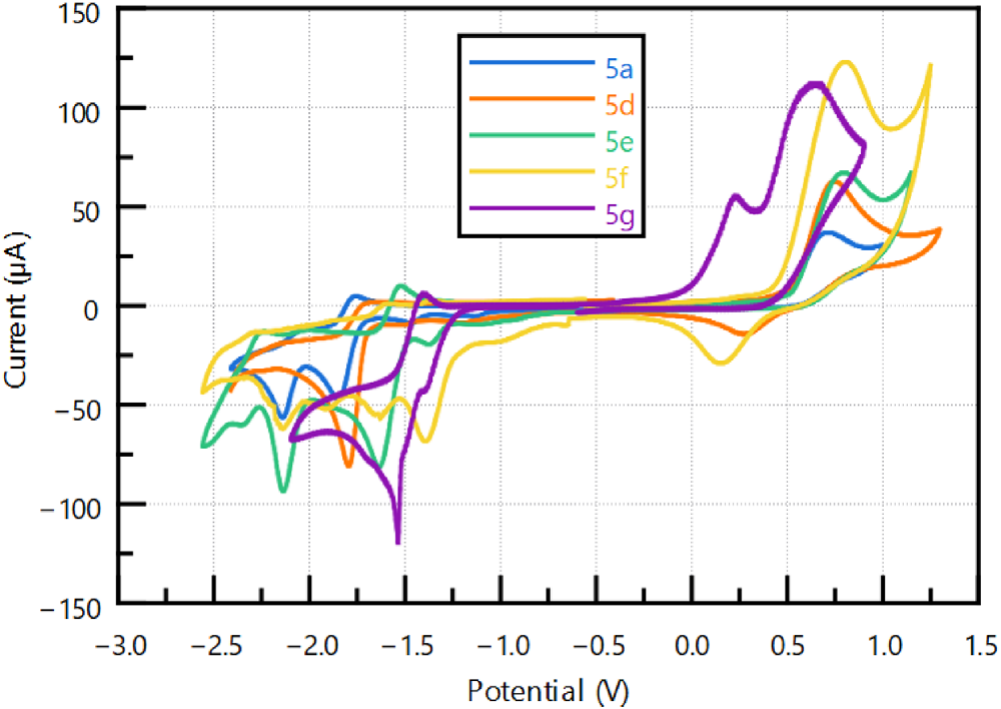
CV of **5a, 5d, 5e, 5f, and 5g** (5 mM) in MeCN/TBAP (0.15 M) at 100 mV/s. Potentials referenced to the Fc/Fc^+^ couple.

*N*‐Arylquinolinium salts bear structural similarity to *N*‐aryl betaine dyes which are notable for their photophysical properties, such as solvatochromism [55, 56] while pyridinium/quinolinium‐based compounds have been used in anion sensing [57] and as color‐tunable liquid crystals [58]. Given this, we sought to determine any photochemical‐based structure‐activity relationships which may exist in the library of quinolinium salts (Figure 6). The UV–vis absorption spectra of the piperidyl series **5d–f** compared with the original quinolone starting materials **2a–c** shows a significant increase in absorption and a pronounced bathochromic shift for the salts (Figure 6A). As seen in previous 4‐phenyl‐quinoliniums, the nature of the *N*‐aryl ring appears to have no impact on the absorption [57]. In addition, absorption spectra of the *N*‐naphthyl series of compounds **5c 5f**, **5i**, **5l,** and **5o** demonstrate the effect of the 4‐amino substituent on the absorption (Figure 6B). Interestingly, not much variation is seen among compounds **5c**, **5l** and **5o** but a slight bathochromic shift can be seen in the 4‐piperidyl and 4‐isopropyl salts **5f** and **5i**. This highlights the effect of electron‐donating substituents on the quinolinium species’ absorption properties [59], most likely due to differing π‐conjugation effects as a result of steric hinderance preventing efficient conjugation of the amine lone pair with the quinolinium π‐system [60, 61, 62].

**FIGURE 6:**
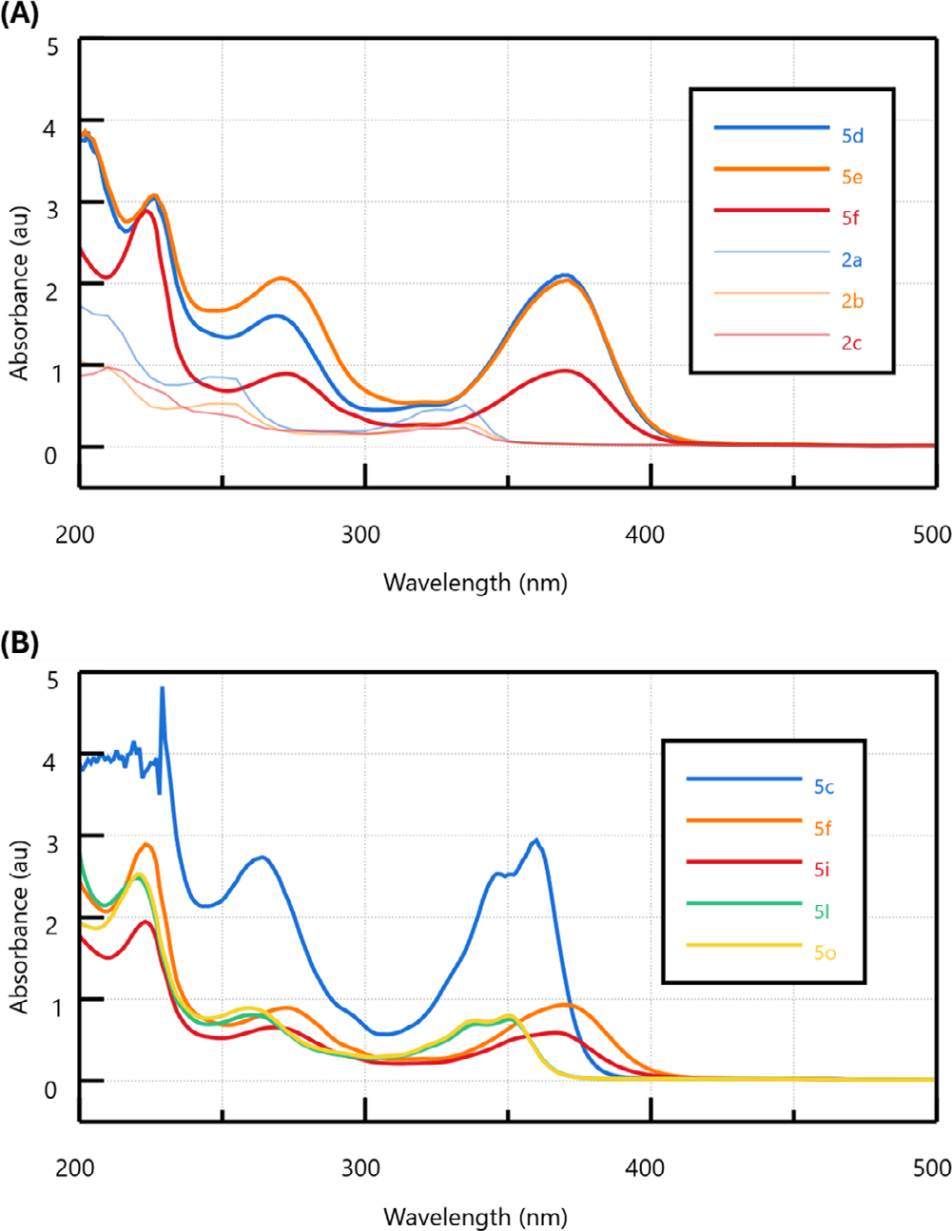
UV–vis absorption spectra of quinolinium salts **5** and neutral quinolones **2**. All spectra were obtained at concentration of 2 mM in MeCN at room temperature. (A) Comparison of absorbance of starting quinolones **2a–c** with the corresponding 4‐piperidinyl‐quinolinium salts **5d–f**. (B) Comparison of influence of 4‐amino substituents **5c** (pyrrolidinyl), **5f** (piperidinyl), **5i** (diisopropylamino), **5l** (benzylamino) and **5o** (ethanolamino) in MeCN.

## 3 Conclusions

We report a novel method to access atropisomeric *N*‐aryl‐quinolinium salts via a stereoretentive S_N_Ar strategy. Primary and secondary amine nucleophiles are tolerated, permitting access to a series of novel axially chiral 4‐aminoquinolinium salts. The structure of these compounds was examined by single crystal x‐ray diffraction, showing significant conjugation of the exocyclic amine lone pair into the electron‐deficient quinolinium ring. DFT analysis showed the compounds display thermal racemization half‐lives of millennia. The electro‐ and photochemical properties of the salts were investigated by CV and UV–vis, respectively, and the presence of clear structure‐activity relationships highlights the tunability of the quinolinium platform. We suggest that this class of atropisomeric *N*‐aryl‐4‐aminoquinolinium salts may find use in asymmetric phase‐transfer catalysis [63, 64, 65, 66], or as chiral α‐cationic ligands in Pt or Au catalysis [38, 67]. The tunable photophysical properties and accessible oxidation potentials of these materials, comparable to established trisaminocyclopropenium electrophotocatalysts, suggest that further investigation of their potential in electrophotocatalysis is warranted [68, 69].

## 4 Experimental Section

### 4.1 General Procedure for the Synthesis of Enaminones (**1**)

A flame‐dried microwave tube was charged with the aryl propynone starting material (1.0 equiv.) and aniline derivatives (1.1 equiv.), evacuated and backfilled with Ar. The mixture was dissolved in MeOH (1.0 M), the tube sealed and stirred at 80°C for 6 h. The mixture was cooled and the desired enaminone product precipitated out of solution and was obtained by vacuum filtration and washed with cold MeOH to afford the desired product.

### 4.2 General Procedure for the Enantioselective Synthesis of Quinolin‐4‐ones (**2**)

A flame‐dried microwave tube was charged with enaminone **1** (0.1 mmol) and (*R*)‐BINAP (12 mg, 0.020 mmol) and evacuated and backfilled with Ar three times. To the flask was added toluene:dioxane (1:1 *v*:*v*, 1 mL) and the solution was sparged with Ar for 5 min. Then, powdered NaOH (6 mg, 0.15 mmol) was added and the solution sparged with Ar for a further 5 min. Pd(OAc)_2_ (2 mg, 0.01 mmol) was added and the solution was sparged for 5 min, turning bright red, quickly sealed and stirred at 45°C for 21 h. The resulting solution was filtered through celite, washed with DCM and concentrated in vacuo purified by flash column chromatography to afford the quinolin‐4‐ones as amorphous solids.

Reactions were scaled up by repeating the above procedure on the same scale in five separate microwave vials. The resulting solutions were filtered through celite, washed with DCM, combined and concentrated in vacuo before purification as described above.

### 4.3 General Procedure for the Enantioretentive Synthesis of Quinolinium Salts (**5**)

A room temperature flame‐dried round bottom flask was flushed with Ar and charged with quinolin‐4‐one **2** and subsequently evacuated and backfilled with Ar. DCM was added and stirred until full dissolution was observed. (COCl)_2_ was added, causing effervescence for approximately 1‐2 min and the resulting bright yellow solution was left to stir for 1 h. The volatiles were removed in vacuo and the flask was flushed with Ar and the resulting sticky yellow solid redissolved in MeCN. While stirring, the amine was added in one portion and the resulting mixture was stirred for 1 h (color change dependent on amine used). Volatiles were removed in vacuo. [Where primary amine nucleophiles were used, the reaction mixture was concentrated in vacuo and redissolved in DCM, washed with 0.1 N HCl (ca. 10 mL/mmol), dried over anhydrous MgSO_4_, filtered and concentrated in vacuo.] The crude material was purified by flash column chromatography to afford the desired quinolinium salt.

### 4.4 Synthesis of Exocyclic Imine (**6**)

Quinolinium salt **5l** (25 mg, 0.05 mmol, 71% *ee*) was dissolved in DCM (1 mL). In a separating funnel, this solution was shaken with aqueous 1.0 M NaOH (3 x 1 mL) and the organic layer was separated, dried over anhydrous MgSO_4_, filtered and concentrated in vacuo to yield the desired imine **6** as an orange solid (22 mg, quantitative). Resolution of the enantiomers of **6** was not achieved by chiral HPLC. However, hydrolysis of **6** under aqueous acidic conditions (1 M HCl) led to recovery of the corresponding quinolin‐4‐one **2c** with no loss in *ee* (73%).

## Conflicts of Interest

The authors declare no conflicts of interest.

## Supporting information



The authors have cited additional references within the Supporting Information [70, 71, 72, 73, 74, 75, 76, 77].
Full experimental procedures, additional discussion and spectroscopic data are provided in the Supporting Information.
DFT‐optimised structures and Gaussian out files generated in the course of this study are openly available from Queen's Research Portal (Data Repository) under the DOI: 10.17034/daa2c78b‐9801‐44c8‐a35c‐f772ba19ac5a.

## Data Availability

The data that supports the findings of this study are available in the supplementary material of this article
