# Supplementary material for: Stereoretentive Nucleophilic Aromatic Substitution Accesses Atropisomeric 4‐Amino‐N‐Arylquinolinium Salts
Source: Chemistry. 2026 Jun 20;32(37):e71266. doi: 10.1002/chem.71266 (PMC13628526; doi:10.1002/chem.71266)
Supplement: Supplementary file 1 [file CHEM-32-e71266-s001.docx]

**Stereoretentive Nucleophilic Aromatic Substitution Accesses Atropisomeric 4-Amino-N-arylquinolinium Salts**

***Supporting Information***

Darren Holmes, Nia Foster, Saima Ansari, Paul Kavanagh, Paul Dingwall and Peter C. Knipe

Contents

[1. General Experimental	4](#_Toc224115753)

[1.1 Naming and Numbering Compounds	4](#_Toc224115754)

[1.2 Solvents and Reagents	4](#_Toc224115755)

[1.3 Chromatography	4](#_Toc224115756)

[1.4 Spectroscopy	4](#_Toc224115757)

[1.5 Electrochemisty	5](#_Toc224115758)

[1.6 Crystallography	5](#_Toc224115759)

[2. Experimental Procedures and Characterization Data	6](#_Toc224115760)

[2.1 Enaminones	6](#_Toc224115761)

[2.2 Atropisomeric *N*-aryl-quinolin-4-ones	9](#_Toc224115762)

[2.3 *N*-aryl-4-aminoquinolinium Salts	11](#_Toc224115763)

[2.3.1 Optimization of Quinolinium Salt Synthesis	11](#_Toc224115764)

[2.3.2 Synthesis of N-aryl-4-aminoquinolinium Salts	12](#_Toc224115765)

[2.4 Synthesis of Axially Chiral Imine	21](#_Toc224115766)

[3. Unsuccessful Reactions	22](#_Toc224115767)

[3.1 Atroposelective Synthesis of N-aryl-quinolin-4-ones	22](#_Toc224115768)

[3.2 Formation of Quinolinium Salts	23](#_Toc224115769)

[3.3 Synthesis of Quinolinimines	24](#_Toc224115770)

[4. Density Functional Theory Calculations	25](#_Toc224115771)

[Methods	25](#_Toc224115772)

[Coordinates	25](#_Toc224115773)

[5b (Ground State)	25](#_Toc224115774)

[5b (Transition State)	26](#_Toc224115775)

[5c (Ground State)	26](#_Toc224115776)

[5c (Transition State)	27](#_Toc224115777)

[5i (Ground State)	27](#_Toc224115778)

[5i (Transition State)	28](#_Toc224115779)

[6 (Ground State)	29](#_Toc224115780)

[6 (Transition State)	29](#_Toc224115781)

[5. HPLC Data	31](#_Toc224115782)

[5.1 Quinolin-4-ones (2)	31](#_Toc224115783)

[5.2 4-amino-quinolinium salts (5)	41](#_Toc224115784)

[6. Cyclic Voltammetry	61](#_Toc224115785)

[7. UV-vis Data	64](#_Toc224115786)

[7.1 Quinolin-4-ones (2)	64](#_Toc224115787)

[7.2 4-amino-quinolinium salts (5)	65](#_Toc224115788)

[8. NMR Spectra	68](#_Toc224115789)

[9. X-ray Crystallography	93](#_Toc224115790)

[Single Crystal Data for 5k (CCDC 2534670)	93](#_Toc224115791)

[10. References	94](#_Toc224115792)

# 1. General Experimental

## 1.1 Naming and Numbering Compounds

Systematic compound names are those generated by ChemDraw Professional version 15.0.0.106 (Perkin Elmer) following IUPAC nomenclature.

## 1.2 Solvents and Reagents

Reactions were carried out under a nitrogen atmosphere in flame-dried glassware unless otherwise stated. Standard inert atmosphere techniques were used in handling all air- and moisture-sensitive reagents. Where necessary solvents were degassed prior to use by sparging with argon or nitrogen (3 hours). Anhydrous and peroxide-free 1,4-dioxane was obtained by percolation through basic activated alumina. Other solvents and reagents were used directly as received from commercial suppliers.

## 1.3 Chromatography

Flash column chromatography was carried out using Fluorochem 60 40-63 micron silica gel. Thin-layer chromatography was carried out using Merck Kieselgel 60 F254 (230-400 mesh) fluorescent treated silica, visualized under UV light (254 nm) or by staining with aqueous potassium permanganate solution, ninhydrin or ceric ammonium molybdate solutions.

## 1.4 Spectroscopy

^1^H and ^13^C NMR spectra were recorded using a Bruker 600 or 400 MHz spectrometer running TopSpin^TM^ software and are quoted in ppm for measurement against tetramethylsilane. Where no tetramethylsilane was present, spectra are referenced relative to the residual non-deuterated solvent peaks. Unless otherwise stated spectra were acquired at 298 K. Topspin^TM^ was used for processing and viewing NMR data. Chemical shifts (δ) are given in parts per million (ppm), and coupling constants (J) are given in Hertz (Hz). The ^1^H NMR spectra are reported as follows: δ / ppm (number of protons, multiplicity, coupling constant J / Hz (where appropriate), assignment). Multiplicity is abbreviated as follows: s = singlet, br = broad, d = doublet, t = triplet, q = quartet, m = multiplet. The numbering scheme used for NMR assignment is arbitrary and does not follow any particular convention. The ^13^C NMR spectra are reported in δ / ppm. Where necessary or appropriate, two-dimensional (COSY, HSQC, HMBC, NOESY or ROESY) NMR experiments were used to assist the assignment of signals in the ^1^H and ^13^C NMR spectra. In some cases, complete assignment of spectra was not possible (in particular, aromatic CHs corresponding to multiple phenyl groups overlapped significantly); in these cases only a partial assignment is reported.

Infra-red (IR) spectra were recorded on an Agilent Cary 630 spectrometer equipped with an attenuated total refractance (ATR) accessory. Samples were deposited on the ATR as a thin film. Only selected maximum absorbances (ν_max_) of the most intense peaks are reported (cm^-1^).

Liquid chromatography-mass spectrometry (LCMS) analysis was conducted using an instrument comprising an Agilent 1260 HPLC (equipped with Infinity II quaternary pump, vial sampler, integrated column compartment and variable wavelength detector) and MSD single quadrupole mass S4 spectrometer. Samples were analysed using an Agilent Infinitylab poroshell 120 column (2.7 μm, 2.1 x 150 mm) under an acetonitrile/water gradient with 0.1% HCOOH additive.

High resolution mass spectra (HRMS) were recorded by Analytical Services and Environmental Projects (ASEP) at Queen’s University Belfast on a Waters LCT Premier ToF mass spectrometer using the electrospray ionisation (ESI) technique.

UV-Vis spectra recorded on an Agilent Cary 60 spectrophotometer at a scan rate of 600 nm/min over 1 nm increments from 200-800 nm.

## 1.5 Electrochemisty

Cyclic voltammetry experiments were performed using a Metrohm Autolab potentiostat (NOVA software) in a 5mL glass electrochemical cell equipped with a 3mm diameter glassy carbon working electrode, a silver/silver chloride leakless electrode (eDAQ: ET072-1), and a titanium wire counter electrode. Measurements were conducted in acetonitrile containing tetrabutylammonium perchlorate (TBAP) (0.15M) as supporting electrolyte. Solutions were purged with argon prior to measurement. Potentials are reported relative to the ferrocene/ferrocenium (Fc/Fc⁺) redox couple added as an internal reference.

## 1.6 Crystallography

Low temperature^[1]^ single crystal Xray diffraction studies were carried out using CuK*_α_* radiation on an Agilent Supernova EoS S2 diffractometer equipped with an area detector and graphite monochromator. CrysAlis^PRO^ software was used for the data collection, integration, reduction and finalisation.^[2]^ Structure solution was performed using direct methods with ShelXT^[3]^ and refined by full matrix least-squares on *F*^2^ with ShelXL^[4]^ on Olex2 software.^[5]^ Full refinement details are given in the supplementary material (CIF). CCDC 2534670 (5k) contain the supplementary crystallographic data for this paper. These data are provided free of charge by The Cambridge Crystallographic Data Centre and copies can be obtained free of charge *via* [www.ccdc.cam.ac.uk/data_request/cif](https://www.ccdc.cam.ac.uk/data_request/cif).

# 2. Experimental Procedures and Characterization Data

**1-(2-Bromophenyl)-3-phenylprop-2-yn-1-one (S1)**

This compound was prepared according to the literature procedure.^[6]^ An oven-dried microwave vial was charged with 2-bromobenzoyl chloride (5.00 g, 22.8 mmol, 1.2 equiv.), Pd(PPh_3_)_2_Cl_2_ (267 mg, 0.38 mmol, 2.0 mol%) and Et_3_N (3.17 mL, 22.8 mmol, 1.2 equiv.), then THF (80 mL, 0.25M) was added and the resulting solution was stirred for 10 min. CuI (145 mg, 0.76 mmol, 4.0 mol%) was then added and allowed to stir for a further 10 min. Phenylacetylene (2.08 mL, 19 mmol, 1.0 equiv.) was added, resulting in a colour change from orange to dark green-black solution and allowed to stir at room temperature for 18 h. The resulting reaction mixture was quenched with 0.1 N HCl (ca. 2.0 mL/mmol), washed with saturated NH_4_Cl aqueous solution (*ca*. 10 mL/mmol), extracted with DCM (*ca*. 3 x 10 mL/mmol) and dried over anhydrous magnesium sulfate. The solvent was removed *in vacuo* and the resulting crude material was purified by column chromatography (silica gel, 5 % EtOAc/PE) to afford the product **S1** as an orange oil (3.96 g, 73 % yield). **^1^H NMR** (400 MHz, CDCl_3_): δ_H_ = 8.06 (1H, dd, J = 7.6, 1.8 Hz), 7.71(1H, dd, J = 7.9, 1.0 Hz), 7.65 (2H, m), 7.51-7.35 (5H, m). **^13^C NMR** (101 MHz, CDCl_3_): δ_C_ = 177.4, 137.4, 135.0, 133.5, 133.1, 132.8, 131.1, 128.8, 127.5, 121.2, 119.9, 94.2, 88.0. The data obtained are consistent with that reported in the literature.^[6]^

## 2.1 Enaminones

**General Procedure A (preparation of enaminones)**

Prepared according to the literature procedure.^[7]^ A flame-dried microwave tube was charged with **S1** (1.0 equiv.) and aniline derivatives (1.1 equiv.), evacuated and backfilled with Ar. The mixture was dissolved in MeOH (1.0 M), the tube sealed and stirred at 80 °C for 6 h. The mixture was cooled and the desired enaminone product precipitated out of solution and was obtained by vacuum filtration and washed with cold MeOH to afford the desired product. The data obtained are consistent with that reported in the literature.^[7-8]^

**(*Z*)-1-(2-bromophenyl)-3-((2,3-dimethylphenyl)amino)-3-phenylprop-2-en-1-one (1a)**

Prepared according to **General Procedure A** using **S1** (570 mg, 2.0 mmol, 1.0 equiv.), 2,3‑dimethylaniline (241 μL, 2.0 mmol, 1.0 equiv.) and MeOH (2 mL). **1a** was obtained as a yellow, crystalline solid (739 mg, 91%).

**^1^H NMR** (400 MHz, CDCl_3_): δ_H_ = 12.60 (1H, s, N-H), 7.61 (1H, dd, J = 7.8, 1.0 Hz), 7.37-7.28 (4H, m), 7.27-7.20 (3H, m), 6.87 (1H, d, J = 7.5 Hz), 6.76 (1H, t, J = 7.8 Hz), 6.45 (1H, d, J = 7.8 Hz), 5.76 (1H, s), 2.36 (3H, s), 2.28 (3H, s). **^13^C NMR** (101 MHz, CDCl_3_), δ_C_ = 191.5, 162.7, 143.2, 137.9, 137.7, 135.5, 134.4, 130.4, 129.7, 129.4, 128.4, 128.3, 127.2, 126.9, 125.2, 123.7, 119.5, 100.1, 20.6, 14.3

**(Z)-1-(2-Bromophenyl)-3-(naphthalen-1-ylamino)-3-phenylprop-2-en-1-one (1b)**

Prepared according to **General Procedure A** using **S1** (570 mg, 2.0 mmol, 1.0 equiv.), 1‑naphthylamine (286 mg, 2.0 mmol, 1.0 equiv.) and MeOH (2 mL). **1b** was obtained as an orange solid (677 mg, 79%).

**^1^H NMR** (400 MHz, CDCl_3_): δ_H_ = 13.06, (1H, s, N-H), 8.41, (1H, d, J = 8.2 Hz), 7.85 (1H, d, J = 8.0 Hz), 7.69-7.49 (5H, m), 7.43‑7.28 (4H, m), 7.25-7.17 (2H, m), 7.12 (1H, t, J = 7.7 Hz), 6.71 (1H, d, J = 7.5 Hz), 5.91 (1H, s), 3.47 (1H, s). **^13^C NMR** (101 MHz, CDCl_3_): δ_C_ = 191.9, 162.9, 143.0, 135.4, 135.3, 134.1, 133.5, 130.6, 128.6, 128.4, 128.3, 128.2, 129.8, 129.4, 127.3, 126.9, 126.5, 125.4, 125.0, 122.7, 122.4, 119.6, 101.2

**(*Z*)-1-(2-bromophenyl)-3-((2-isopropylphenyl)amino)-3-phenylprop-2-en-1-one (1c)**

Prepared according to **General Procedure A** using **S1** (570 mg, 2.0 mmol, 1.0 equiv.), 2‑isopropylaniline (283 μL, 2.0 mmol, 1.0 equiv.) and MeOH (2 mL). **1c** was obtained as a yellow powder (681 mg, 81%).

**^1^H NMR** (400 MHz, CDCl_3_): δ_H_ = 12.74 (1H, s, N-H), 7.62 (2H, d, *J* = 8.1 Hz), 7.55 (1H, d, *J* = 7.7 Hz), 7.38-7.26 (7H, m), 7.26-7.21 (1H, m), 7.06 (1H, t, *J* = 7.4 Hz), 6.85 (1H, t, *J* = 8.5 Hz), 6.54 (1H, d, *J* = 8.1 Hz), 5.79 (1H, s), 3.49 (1H, sept), 1.36 (6H, d, *J* = 6.9 Hz). **^13^C NMR** (101 MHz): δ_C_ = 191.5, 162.5, 143.2, 141.9, 136.6, 135.3, 133.4, 130.4, 129.7, 129.3, 128.43, 128.41, 127.2, 126.2, 126.0, 125.62, 125.61, 119.5, 100.4, 28.3, 23.2,

**(Z)-1-(2-bromophenyl)-3-((2-*tert*-butylphenyl)amino)-3-phenylprop-2-en-1-one (1d)**

Prepared according to **General Procedure A** using **S1** (570 mg, 2.0 mmol, 1.0 equiv.), 2‑*tert*‑butylaniline (312 μL, 2.0 mmol, 1.0 equiv.) and MeOH (2 mL). **1d** was obtained as a yellow powder (433 mg, 50%).

**^1^H NMR** (400 MHz, CDCl_3_): δ_H_ = 12.85 (1H, s, N-H), 7.57 (1H, d, *J* = 8.0 Hz), 7.51 (1H, dd, *J* = 6.0, 1.7 Hz), 7.39 (1H, d, *J* = 8.0 Hz), 7.34-7.26 (2H, m), 7.25-7.16 (4H, m), 6.98 (1H, t, *J* = 7.8 Hz), 6.78 (1H, t, *J* = 7.7 Hz), 6.45 (1H, d, *J* = 7.7 Hz), 5.75 (1H, s), 1.60 (9H, s). **^13^C NMR** (101 MHz): δ_C_ = 191.4, 161.7, 143.3, 143.1, 137.5, 135.4, 133.3, 130.4, 129.6, 129.2, 128.9, 127.2, 126.8, 125.9, 125.5, 119.5, 100.6, 35.2, 30.7.

**(*Z*)-1-(2-bromophenyl)-3-((2-methoxyphenyl)amino)-3-phenylprop-2-en-1-one (1e)**

Prepared according to **General Procedure A** using **S1** (570 mg, 2.0 mmol, 1.0 equiv.), 2‑methoxyaniline (225 μL, 2.0 mmol, 1.0 equiv.) and MeOH (2 mL). **1e** was obtained as a yellow powder (635 mg, 78%).

**^1^H NMR** (400 MHz, CDCl_3_): δ_H_ = 12.47 (1H, s, N-H), 7.60 (1H, d, *J* = 7.9 Hz), 7.54 (1H, d, *J* = 7.7 Hz), 7.42-7.29 (6H, m), 7.22 (1H, t, *J* = 7.8 Hz), 6.97 (1H, t, *J* = 7.9 Hz), 6.87 (1H, d, *J* = 7.8 Hz), 6.58 (1H, t, *J* = 7.7 Hz), 5.74 (1H, s), 3.90 (3H, s). **^13^C NMR** (101 MHz): δ_C_ = 191.3, 160.8, 151.3, 143.2, 135.8, 133.4, 130.4, 129.8, 129.4, 128.5, 128.1, 127.2, 124.9, 123.6, 120.0, 119.5, 110.9, 101.2, 55.6.

## 2.2 Atropisomeric *N*-aryl-quinolin-4-ones

**General Procedure B (Kitagawa Cyclization)**

**0.1 mmol scale:** Prepared in analogy to the method described in the literature procedure.^[7]^ A flame-dried microwave tube was charged with enaminone **1** (1.0 equiv.) and (*R*)-BINAP (20 mol%) and evacuated and backfilled with Ar three times. To the flask was added toluene:dioxane (1:1) (0.1 M) and the solution was sparged with Ar for 5 min. Then, NaOH (1.5 equiv.) was added and the solution sparged with Ar for a further 5 min. Pd(OAc)_2_ (10 mol%) was added and the solution was sparged for 5 min, turning bright red, quickly sealed and stirred at 45 °C for 21 h. The resulting solution was filtered through celite®, washed with DCM and concentrated *in vacuo* purified by flash column chromatography to afford the quinolin-4-ones as amorphous solids.

**0.5 mmol scale:** 5 individual microwave vials were flame-dried and charged with enaminone **1** and (1.0 equiv.) and (*R*)-BINAP (20 mol%) and evacuated and backfilled with Ar three times. To each flask was added toluene:dioxane (1:1) (0.1 M) and the solutions were sparged for 5 min. Then, NaOH (1.5 equiv.) was added to the solution and sparged for a further 5 min. Pd(OAc)_2_ (10 mol%) was added and the solutions were sparged for 5 min, turning bright red, quickly sealed and stirred at 45 °C for 21 h in a large oil bath. The resulting solutions were filtered through celite®, washed with DCM, combined and concentrated *in vacuo*. The resulting mixed crude material was purified by flash column chromatography to afford the quinolin-4-ones as amorphous solids. The data obtained are consistent with those reported in the literature.^[7, 9]^

**1-(2,3-dimethylphenyl)-2-phenylquinolin-4(*1H*)-one (2a)**

Prepared according to **General Procedure B** using enaminone **1a** (41 mg, 0.1 mmol, 1.0 equiv.), (*R*)-BINAP (12 mg, 0.02 mmol, 20 mol%), tol:dioxane (1:1) (1 mL), NaOH (6 mg, 1.5 equiv.), Pd(OAc)_2_ (2 mg, 0.01 mmol, 10 mol%). Crude mixture purified by flash column chromatography (silica gel, 50% EtOAc/PE) to afford **2a** as an orange solid (**0.1 mmol scale**: 23 mg, 74 %, 92 % *ee*) (**0.5 mmol scale:** 113 mg, 70 %, 83 % *ee*).

**^1^H NMR** (400 MHz, CDCl_3_): δ_H_ = 8.53 (1H, dd, J = 7.8, 1.4 Hz), 7.49-7.43 (1H, m), 7.40-7.35 (1H, m), 7.24-7.16 (5H, m), 7.15‑7.07 (2H, m), 7.04-6.98 (1H, m), 6.76 (1H, d, J = 8.69 Hz), 6.44 (1H, s), 2.20 (3H, s), 1.81 (3H, s). **^13^C NMR** (101 MHz, CDCl_3_): δ_C_ = 178.0, 154.1, 142.1, 138.9, 137.9, 135.5, 135.2, 132.0, 130.7, 128.8, 128.7, 128.0, 127.7, 126.3, 126.2, 126.1, 123.8, 117.8, 112.6, 20.2, 14.4. **HPLC**: CHIRALART Cellulose‑SB, 15 % IPA/Hex, 1 mL/min, λ=240 nm, t_R_ (major) = 11.4 min, t_R_ (minor) = 13.2 min. **HRMS** (ES+): found 326.1551; C_23_H_19_NO, [M+H]^+^ requires 326.1539.

**1-(2-isopropylphenyl)-2-phenylquinolin-4(*1H*)-one (2b)**

Prepared according to **General Procedure B** using enaminone **1b** (43 mg, 0.1 mmol, 1.0 equiv.), (*R*)-BINAP (12 mg, 0.02 mmol, 20 mol%), tol:dioxane (1:1) (1 mL), NaOH (6 mg, 1.5 equiv.), Pd(OAc)_2_ (2 mg, 0.01 mmol, 10 mol%). Crude mixture purified by flash column chromatography (silica gel, 50% EtOAc/PE) to afford **2b** as an orange solid (**0.1 mmol scale**: 17 mg, 52 %, 85% *ee*) (**0.5 mmol scale:** 80 mg, 48 %, 69 % *ee*).

**^1^H NMR** (400 MHz, CDCl_3_): δ_H_ = 8.52 (1H, d, *J* = 7.9 Hz), 7.45 (1H, t, *J* = 7.5 Hz), 7.40-7.32 (3H, m), 7.30-7.27 (1H, m), 7.25-7.16 (6H, m), 6.78 (1H, d, *J* = 8.7 Hz), 6.47 (1H, s), 2.46 (1H, sept), 0.84 (3H, d, *J* = 6.7 Hz), 0.78 (3H, d, *J* = 6.7 Hz). ). **^13^C NMR** (101 MHz, CDCl_3_): δ_C_ = 178.2, 154.2, 147.1, 142.9, 136.5, 135.3, 131.9, 131.1, 130.2, 129.7, 129.0, 127.8, 127.7, 126.5, 126.4, 124.0, 118.5, 112.8, 27.9, 25.1, 22.1. **HPLC**: CHIRALART Cellulose‑SB, 15 % IPA/Hex, 1 mL/min, λ=240 nm, t_R_ (major) = 16.9 min, t_R_ (minor) = 19.56 min. **HRMS** (ES+): found 340.1748; C_24_H_21_NO, [M+H]^+^ requires 340.1696.

**1-(Naphthalen-1-yl)-2-phenylquinolin-4(1H)-one (2c)**

Prepared according to **General Procedure B** using enaminone **1c** (43 mg, 0.1 mmol, 1.0 equiv.), (*R*)-BINAP (12 mg, 0.02 mmol, 20 mol%), tol:dioxane (1:1) (1 mL), NaOH (6 mg, 1.5 equiv.), Pd(OAc)_2_ (2 mg, 0.01 mmol, 10 mol%). Crude mixture purified by flash column chromatography (silica gel, 50% EtOAc/PE) to afford **2c** as an orange solid (**0.1 mmol scale**: 22 mg, 60 %, 91 % *ee*) (**0.5 mmol scale:** 99 mg, 57 %, 73 % *ee*).

**^1^H NMR** (400 MHz, CDCl_3_),: δ_H_ = 8.59-8.53 (1H, m), 7.85 (2H, t, J = 8.23), 7.53-7.31 (7H, m), 7.10‑7.03 (3H, m), 7.02-6.94 (2H, m), 6.70-6.62 (H, m ), 6.53 (1H, s). **^13^C NMR** (101 MHz, CDCl_3_): δ_C_ = 178.0, 154.9, 142.7, 135.6, 135.5, 134.1, 132.1, 131.2, 129.8, 128.7, 128.6, 128.5, 128.4, 127.9, 127.5, 126.9, 126.3, 126.0, 125.2, 123.9, 122.7, 118.3, 112.8. **HPLC:** CHIRALPAK OD-RH, 60 % MeCN/H_2_O, 0.75 mL/min, λ = 250 nm, t_R1_ = 11.70 min, t_R2_ = 14.20 min. **HRMS** (ES+): found 348.1422; C_23_H_19_NO, [M+H]^+^ requires 348.1383.

## 2.3 *N*-aryl-4-aminoquinolinium Salts

### 2.3.1 Optimization of Quinolinium Salt Synthesis

Initially, O-functionalisation to generate quinolinium intermediate **3** and subsequent S_N_Ar reaction with amines to synthesize **5** was carried out according to the procedure described by Tinnermann *et al.^[10]^* We assumed less harsh conditions would be required to for functionalisation of **2a** due to the carbonyl group being in a less hindered position, *para* to the axially chiral C-N bond. Therefore, we optimized the reaction to be carried out on more mild conditions.

|  | | | | |
| --- | --- | --- | --- | --- |
| Entry | Temperature (°C) | Time (h) | Reactant equivalents | Yield (%) |
| 1 | 80 | 18 | 3.0 | 62 |
| 2 | rt | 1 | 3.0 | 60 |
| 3 | rt | 1 | 5.0 | 69 |

Table S1. Optimization of the synthesis of quinolinium salt **5** via treatment of **2** with oxalyl chloride and subsequent reaction with pyrrolidine. Both steps were reacted under the same reaction conditions described in the table. Yields provided are isolated yields of **5**.

### 2.3.2 Synthesis of N-aryl-4-aminoquinolinium Salts

**General Procedure C**

A room temperature flame-dried round bottom flask was flushed with Ar and charged with quinolin-4-one **2** and subsequently evacuated and backfilled with Ar. DCM was added and stirred until full dissolution was observed. (COCl)_2_ was added, causing effervescence for approximately 1-2 minutes and the resulting bright yellow solution was left to stir for 1 h. The volatiles were removed *in vacuo* and the flask was flushed with Ar and the resulting sticky yellow solid redissolved in MeCN. While stirring, the amine was added in one portion and the resulting mixture was stirred for 1 h (colour change dependent on amine used). Volatiles were removed *in vacuo* and the crude material purified by flash column chromatography to afford the desired quinolinium salt.

**General Procedure D**

A room temperature flame-dried round bottom flask was flushed with Ar and charged with quinolin-4-one **2** and subsequently evacuated and backfilled with Ar. DCM was added and stirred until full dissolution was observed. (COCl)_2_ was added, causing effervescence for approximately 1-2 minutes and the resulting bright yellow solution was left to stir for 1 h. The volatiles were removed *in vacuo* and the flask was flushed with Ar and the resulting sticky yellow solid redissolved in MeCN. While stirring, the amine was added in one portion and the resulting mixture was stirred for 1 h (colour change dependent on amine used). The reaction mixture was concentrated *in vacuo* and redissolved in DCM, washed with 0.1 N HCl (ca. 10 mL/mmol), dried over anhydrous MgSO_4_, filtered and concentrated *in vacuo*. The resulting crude material was purified by flash column chromatography to afford the desired quinolinium salt.

**1-(2,3-dimethylphenyl)-2-phenyl-4-(pyrrolidin-1-yl)quinolin-1-ium chloride (5a)**

Prepared according to **General Procedure C**, using quinolin-4-one **2a** (33 mg, 0.1 mmol, 1.0 equiv., 83 % *ee*), (COCl)_2_ (43 μL, 0.5 mmol, 5.0 equiv.), DCM (1 mL), pyrrolidine (41 μL, 0.5 mmol, 5.0 equiv.), MeCN (1 mL). Crude mixture purified by flash column chromatography (silica gel, 5% MeOH/DCM) to afford **3a** as an orange foam (29 mg, 69 %, 85 % *ee*).

**1H NMR** (600 MHz, CDCl_3_): δ_H_ = 8.74 (1H, d, J = 7.0 Hz), 7.74-7.64 (2H, m), 7.33-7.23 (5H, m), 7.21 (1H, d, *J* = 7.7 Hz), 7.17 (1H, t, *J* = 7.6 Hz), 7.07-6.98 (2H, m), 6.73 (1H, s), 4.50 (2H, bs), 3.94 (2H, bs), 2.29 (4H, bs), 2.22 (3H, s), 1.78 (3H, s). **^13^C NMR** (101 MHz, CDCl_3_): δ_C_ = 155.8, 154.5, 141.6, 139.6, 136.7, 134.4, 133.9, 133.6, 131.8, 130.1, 129.2, 128.44, 128.42, 127.6, 127.4, 127.0, 126.3, 119.2, 118.5, 105.3, 98.8, 92.7, 91.9, 91.7, 65.0, 54.3, 20.3, 15.0. **IR:** ν_max_ (thin film): 2931, 1997, 1610, 1422, 1339, 1267, 1026, 779, 766, 702 cm^-1^. **HRMS** (ES+): found 379.2166; C_27_H_27_N_2_ [M+H]^+^ requires 379.2169. **HPLC**: CHIRALART Cellulose-SB, 35% MeCN/H_2_O,0.75 mL/min, λ = 254 nm, t_R_ (major) = 21.7 min, t_R_ (minor) = 24.7 min. [α]_D_^25^ +75.4 (c = 0.05, CHCl_3_, 85 % *ee*).

**1-(2-isopropylphenyl)-2-phenyl-4-(pyrrolidin-1-yl)quinolin-1-ium chloride (5b)**

Prepared according to **General Procedure C**, using quinolin-4-one **2b** (33 mg, 0.1 mmol, 1.0 equiv., 85 % *ee*), (COCl)_2_ (43 μL, 0.5 mmol, 5.0 equiv.), DCM (1 mL), pyrrolidine (41 μL, 0.5 mmol, 5.0 equiv.), MeCN (1 mL). Crude mixture purified by flash column chromatography (silica gel, 5% MeOH/DCM) to afford **3b** as an orange foam (30 mg, 70 %, 85 % *ee*).

**1H NMR** (400 MHz, CDCl_3_): δ_H_ = 8.76 (1H, d, *J* = 8.4 Hz), 7.69 (2H, quint, *J* = 7.2 Hz), 7.48-7.43 (1H, m), 7.36 (2H, d, *J* = 4.0 Hz), 7.34-7.27 (6H, m), 7.03 (1H, d, *J* = 8.5 Hz), 6.77 (1H, s), 4.51 (2H, bs), 4.12-3.79 (2H, m), 2.35-2.22 (5H, m), 0.81 (6H, t, *J* = 7.4 Hz). **^13^C NMR** (101 MHz, CDCl_3_): δ_C_ = 154.4, 153.4, 144.9, 140.8, 134.0, 132.41, 132.44, 130.2, 129.1, 129.0, 128.6, 127.3, 127.2, 127.1, 126.2, 125.1, 118.5, 117.3, 104.0, 26.9, 24.0, 21.0. **IR:** ν_max_ (thin film): 2939, 2792, 1967, 1594, 1552, 1447, 1356, 1242, 1034, 771, 658. **HRMS** (ES+): found 393.2433; C_27_H_27_N_2_ [M+H]^+^ requires 393.2325 **HPLC**: CHIRALCEL OD-RH, 50% MeCN/H_2_O, 0.75 mL/min, λ = 254 nm, t_R_ (major) = 14.2 min, t_R_ (minor) = 19.0 min. [α]_D_^25^ -53.7 (c = 0.05, CHCl_3_, 85 % *ee*).

**1-(naphthalen-1-yl)-2-phenyl-4-(pyrrolidin-1-yl)quinolin-1-ium chloride (5c)**

Prepared according to **General Procedure C**, using quinolin-4-one **2c** (33 mg, 0.1 mmol, 1.0 equiv., 73 % *ee*), (COCl)_2_ (43 μL, 0.5 mmol, 5.0 equiv.), DCM (1 mL), pyrrolidine (41 μL, 0.5 mmol, 5.0 equiv.), MeCN (1 mL). Crude mixture purified by flash column chromatography (silica gel, 5% MeOH/DCM) to afford **3c** as an orange foam (31 mg, 73 %, 76 % *ee*).

**1H NMR** (400 MHz, CDCl_3_): δ_H_ = 8.76 (1H, d, J = 8.76 Hz), 7.90 (2H, t, J = 6.4 Hz), 7.67 (1H, t, J = 7.6 Hz), 7.58-7.50 (3H, m), 7.49-7.42 (2H, m), 7.30 (1H, d, *J* = 8.1 Hz), 7.20 (2H, d, *J* = 7.5 Hz), 7.15 (1H, t, *J* = 7.7 Hz), 7.06 (2H, t, *J* = 7.6 Hz), 6.92 (1H, d, J = 8.7 Hz), 6.81 (1H, s), 4.56 (2H, bs), 3.99 (2H, bs), 2.32 (4H, bs). **^13^C NMR** (101 MHz, CDCl_3_): δ_C_ = 155.7, 155.3, 141.8, 134.2, 134.1, 133.9, 133.8, 130.9, 130.2, 130.0, 128.9, 128.8, 128.5, 128.4, 128.3, 127.8, 127.4, 126.0, 125.5, 122.1, 119.7, 118.4, 105.2, 29.7. **IR:** ν_max_ (thin film): 2923, 2853, 2172, 2015, 1593, 1552, 1422, 1333, 1239, 1017, 774, 702. **HRMS** (ES+): found 401.2126; C_27_H_27_N_2_ [M+H]^+^ requires 401.2012.2169. **HPLC**: CHIRALPAK OD-RH, 50% MeCN/H_2_O, 0.75 mL/min, λ = 254 nm, t_R_ (major) = 13.1 min, t_R_ (minor) = 18.9 min. [α]_D_^25^ +40.0 (c = 0.1, CHCl_3_, 76 % *ee*).

**(R)-1-(2,3-dimethylphenyl)-2-phenyl-4-(piperidin-1-yl)quinolin-1-ium chloride (5d)**

Prepared according to **General Procedure C**, using quinolin-4-one **2a** (33 mg, 0.1 mmol, 1.0 equiv., 83 % *ee*), (COCl)_2_ (43 μL, 0.5 mmol, 5.0 equiv.), DCM (1 mL), piperidine (49 μL, 0.5 mmol, 5.0 equiv.), MeCN (1 mL). Crude mixture purified by flash column chromatography (silica gel, 5% MeOH/DCM) to afford **3d** as an orange foam (42 mg, 95 %, 85 % *ee*).

**1H NMR** (400 MHz, CDCl_3_): δ_H_ = 8.25 (1H, d, *J* = 8.2 Hz), 7.73 (1H, t, *J* = 7.7 Hz), 7.68 (1H, t, *J* = 7.7 Hz), 7.34-7.26 (5H, m), 7.23-7.16 (2H, m), 7.13 (1H, d, *J* = 7.5 Hz), 7.06 (2H, d, *J* = 7.8 Hz), 4.13-4.06 (4H, m), 2.22 (3H, s), 1.99-1.88 (6H, m), 1.76 (3H, s). **^13^C NMR** (101 MHz, CDCl_3_): δ_C_ = 160.6, 155.8, 141.6, 139.7, 136.6, 134.4, 134.1, 133.5, 132.0, 130.4, 129.0, 128.5, 127.6, 127.2, 127.1, 126.6, 119.8, 119.2, 108.1, 54.0, 26.3, 23.7, 20.3, 14.7. **IR:** ν_max_ (thin film): 2937, 2854, 1978, 1588, 1547, 1344, 1242, 1015, 721, 699. **HRMS** (ES+): found 379.2166; C_27_H_27_N_2_ [M+H]^+^ requires 393.2325. **HPLC**: CHIRALART Cellulose-SB, 35% MeCN/H_2_O, 0.75 mL/min, λ = 254 nm, t_R_ (major) = 22.1 min, t_R_ (minor) = 24.8 min. [α]_D_^25^ +30.3 (c = 0.05, CHCl_3_, 85 % ee).

**(R)-1-(2-isopropylphenyl)-2-phenyl-4-(piperidin-1-yl)quinolin-1-ium chloride (5e)**

Prepared according to **General Procedure C**, using quinolin-4-one **2b** (33 mg, 0.1 mmol, 1.0 equiv., 69 % *ee*), (COCl)_2_ (43 μL, 0.5 mmol, 5.0 equiv.), DCM (1 mL), piperidine (49 μL, 0.5 mmol, 5.0 equiv.), MeCN (1 mL). Crude mixture purified by flash column chromatography (silica gel, 5% MeOH/DCM) to afford **3e** as an orange foam (39 mg, 91 %, 69 % *ee*).

**1H NMR** (400 MHz, CDCl_3_): δ_H_ = 8.27 (1H, d, *J* = 8.3 Hz), 7.68 (2H, quint, *J* = 7.1 Hz), 7.53 (1H, d, *J* = 8.0 Hz), 7.47 (1H, t, *J* = 7.5 Hz), 7.42-7.35 (3H, m), 7.35-7.27 (4H, m), 7.12 (1H, s), 7.06 (1H, d, *J* = 8.7 Hz), 4.16-4.08 (4H, m), 2.25 (1H, sept, *J* = 6.8 Hz), 2.00-1.89 (6H, m), 0.81 (3H, d, *J* = 6.8 Hz), 0.77 (3H, d, *J* = (6.7 Hz). **^13^C NMR** (101 MHz, CDCl_3_): δ_C_ = 160.7, 155.8, 145.7, 142.4, 135.1, 133.9, 133.3, 131.5, 130.5, 130.3, 130.0, 128.5, 128.1, 127.7, 127.4, 126.5, 120.3, 119.3, 108.3, 54.0, 28.1, 26.4, 25.2, 23.7, 22.2. **IR:** ν_max_ (thin film): 2928, 2856, 2170, 1594, 1549, 1333, 1125, 768, 705. **HRMS** (ES+): found 407.2487; C_27_H_27_N_2_ [M+H]^+^ requires 407.2482. **HPLC**: CHIRALART Cellulose-SB, 35% MeCN/H_2_O, 0.75 mL/min, λ = 254 nm, t_R_ (major) = 14.1 min, t_R_ (minor) = 20.4 min. [α]_D_^25^ 12.8 (c = 0.05, CHCl_3_, 69 % *ee*).

**(R)-1-(naphthalen-1-yl)-2-phenyl-4-(piperidin-1-yl)quinolin-1-ium chloride (5f)**

Prepared according to **General Procedure C**, using quinolin-4-one **2c** (33 mg, 0.1 mmol, 1.0 equiv.73 % *ee*), (COCl)_2_ (43 μL, 0.5 mmol, 5.0 equiv.), DCM (1 mL), piperidine (49 μL, 0.5 mmol, 5.0 equiv.), MeCN (1 mL). Crude mixture purified by flash column chromatography (silica gel, 5% MeOH/DCM) to afford **3f** as an orange foam (44 mg, 99 %, 75 % *ee*).

**1H NMR** (400 MHz, CDCl_3_): δ_H_ = 8.29 (1H,d, J = 8.4 Hz), 7.97-7.88 (2H, m), 7.70-7.46 (6H, m), 7.32 (3H, m), 7.16 (2H, t, J = 9.3 Hz), 7.08 (2H, t, J = 7.3 Hz), 6.96 (1H, d, J = 8.7 Hz), 4.17 (4H, bs), 2.06‑1.92 (6H, m). **^13^C NMR** (101 MHz, CDCl_3_): δ_C_ = 161.0, 156.5, 142.3, 134.1, 134.13, 134.11, 133.7, 131.0, 130.1, 130.0, 129.0, 128.9, 128.7, 128.6, 128.2, 127.6, 126.3, 125.7, 122.0, 120.3, 119.2, 108.4, 54.1, 29.8, 26.3, 23.7. . **IR:** ν_max_ (thin film): 2928, 2867, 2153, 2039, 1593, 1549, 1330, 1261, 1147, 1012, 776, 729, 693. **HRMS** (ES+): found 415.2147; C_28_H_29_N_2_ [M+H]^+^ requires 415.2169. **HPLC**: CHIRALPAK OD-RH, 50% MeCN/H_2_O, 0.75 mL/min, λ = 254 nm, t_R_ (major) = 11.0 min, t_R_ (minor) = 18.9 min. [α]_D_^25^ +45.6 (c = 0.1, CHCl_3_, 75 % *ee*).

**(R)-4-(diisopropylamino)-1-(2,3-dimethylphenyl)-2-phenylquinolin-1-ium chloride (5g)**

Prepared according to **General Procedure C**, using quinolin-4-one **2a** (33 mg, 0.1 mmol, 1.0 equiv., 83 % *ee*), (COCl)_2_ (43 μL, 0.5 mmol, 5.0 equiv.), DCM (1 mL), diisopropylamine (70 μL, 0.5 mmol, 5.0 equiv.), MeCN (1 mL). Crude mixture purified by flash column chromatography (silica gel, 5% MeOH/DCM) to afford **3g** as an orange foam (27 mg, 60 %).

**1H NMR** (400 MHz, CDCl_3_): δ_H_ = 8.19 (1H, d, *J* = 7.8 Hz), 7.79-7.66 (2H, m), 7.37-7.28 (5H, m), 7.23-7.19 (3H, m), 7.13-7.05 (2H, m), 4.70-4.58 (2H, m), 2.23 (3H, s), 1.78 (3H, s), 1.71-1.65 (12H, m). **^13^C NMR** (101 MHz, CDCl_3_): δ_C_ = 158.9, 154.0, 142.0, 139.6, 136.5, 134.3, 134.1, 133.8, 132.1, 130.4, 128.8, 128.7, 127.5, 127.4, 127.1, 126.3, 120.0, 119.6, 107.8, 53.8, 29.9, 22.4, 22.2, 20.4, 14.9. **IR:** ν_max_ (thin film): 2928, 2856, 2039, 1588, 1544, 1325, 1264, 1139, 727, 696. **HRMS** (ES+): found 409.2737; C_27_H_27_N_2_ [M+H]^+^ requires 409.2638.

**(R)-4-(diisopropylamino)-1-(2-isopropylphenyl)-2-phenylquinolin-1-ium chloride (5h)**

Prepared according to **General Procedure C**, using quinolin-4-one **2b** (33 mg, 0.1 mmol, 1.0 equiv., 69 % *ee*), (COCl)_2_ (43 μL, 0.5 mmol, 5.0 equiv.), DCM (1 mL), diisopropylamine (70 μL, 0.5 mmol, 5.0 equiv.), MeCN (1 mL). Crude mixture purified by flash column chromatography (silica gel, 5% MeOH/DCM) to afford **3h** as an orange foam (27 mg, 60 %).

**1H NMR** (400 MHz, CDCl_3_): δ_H_ = 8.19 (1H, d, *J* = 8.3 Hz), 7.77-7.68 (2H, m), 7.59 (1H, d, *J* = 7.7 Hz), 7.50 (1H, t, *J* = 7.3 Hz), 7.46 (1H, t, *J* = 7.7 Hz), 7.38-7.34 (1H, m), 7.34-7.30 (5H, m), 7.12-7.08 (2H, m), 4.66 (2H, sept, *J* = 6.7 Hz), 2.20 (1H, sept, *J* = 6.8 Hz), 1.73 (6H, d, *J* = 6.7 Hz), 1.65 (6H, d, *J* = 6.7 Hz), 0.85 (3H, d, *J* = 6.8 Hz), 0.75 (3H, d, *J* = 6.8 Hz). **^13^C NMR** (101 MHz, CDCl_3_): δ_C_ = 158.7, 153.8, 145.5, 142.5, 134.7, 133.8, 133.3, 131.5, 130.5, 130.2, 129.4, 128.6, 128.0, 127.6, 127.0, 126.2, 120.3, 119.4, 107.7, 53.7, 47.4, 28.0, 25.1, 22.4, 22.1, 21.9, 19.1. **IR:** ν_max_ (thin film): 2922, 2845, 2166, 1593. **HRMS** (ES+): found 423.2870; C_27_H_27_N_2_ [M+H]^+^ requires 423.2795.

**(R)-4-(diisopropylamino)-1-(naphthalen-1-yl)-2-phenylquinolin-1-ium chloride (5i)**

Prepared according to **General Procedure C**, using quinolin-4-one **2c** (33 mg, 0.1 mmol, 1.0 equiv., 73 % *ee*), (COCl)_2_ (43 μL, 0.5 mmol, 5.0 equiv.), DCM (1 mL), diisopropylamine (70 μL, 0.5 mmol, 5.0 equiv.), MeCN (1 mL). Crude mixture purified by flash column chromatography (silica gel, 5% MeOH/DCM) to afford **3i** as an orange foam (28 mg, 61 %).

**1H NMR** (400 MHz, CDCl_3_): δ_H_ = 8.20 (1H, d, *J* = 8.4 Hz), 7.96 (2H, m), 7.71 (1H, d, 7.1 Hz), 7.65 (1H, t, 7.3 Hz), 7.60 (1H, t, 8.1 Hz), 7.60-7.50 (3H, m), 7.25-7.20 (3H, m), 7.19-7.13 (2H, m), 6.99 (1H, d, *J* = 8.4 Hz), 4.69 (2H, sept), 1.74-1.68 (12H, m). **^13^C NMR** (101 MHz, CDCl_3_): δ_C_ = 159.2, 154.8, 142.6, 135.04, 134.08, 133.94, 133.91, 131.1, 130.2, 129.9, 129.1, 128.9, 128.7, 128.4, 127.4, 126.9, 126.03, 125.99, 121.8, 120.6, 119.4, 107.9, 53.8, 29.8, 22.4, 22.1. **IR:** ν_max_ (thin film): 2931, 2150, 2031, 1590, 1544, 1502, 1327, 1270, 1120, 1015, 771, 726, 696. **HRMS** (ES+): found 431.2493; C_28_H_29_N_2_ [M+H]^+^ requires 431.2482.

**(R)-1-(2,3-dimethylphenyl)-4-((3-methylbenzyl)amino)-2-phenylquinolin-1-ium chloride (5j)**

Prepared according to **General Procedure D**, using quinolin-4-one **2a** (33 mg, 0.1 mmol, 1.0 equiv., 83 % *ee*), (COCl)_2_ (43 μL, 0.5 mmol, 5.0 equiv.), DCM (1 mL), 3-methylbenzylamine (63 μL, 0.5 mmol, 5.0 equiv.), MeCN (1 mL). Crude mixture purified by flash column chromatography (silica gel, 5% MeOH/DCM) to afford **3j** as an orange foam (42 mg, 90 %, 83 % *ee*).

**1H NMR** (400 MHz, CDCl_3_): δ_H_ = 12.12 (1H, bs), 9.70 (1H, d, *J* = 8.4 Hz), 7.78 (1H, t, *J* = 7.7 Hz), 7.66 (1H, t, *J* = 7.9 Hz), 7.35 (1H, bs), 7.33-7.28 (2H, m), 7.23-7.18 (4H, m), 7.14 (1H, t, *J* = 7.9 Hz), 7.07 (1H, d, *J* = 7.7 Hz), 6.99 (2H, d, *J* = 7.3 Hz), 6.96 (1H, d, *J* = 7.7 Hz), 6.93 (1H, d, *J* = 8.7 Hz), 6.55 (1H, s), 4.92 (2H, qd, *J* = 15.7 Hz, 5.4 Hz), 2.31 (3H, s), 2.21 (3H, s), 1.69 (3H, s). **^13^C NMR** (101 MHz, CDCl_3_): δ_C_ = 156.1, 155.7, 139.9, 139.7, 138.7, 136.9, 136.2, 134.5, 134.20, 134.16, 131.8, 130.2, 128.8, 128.7, 128.6, 128.5, 128.3, 127.8, 127.5, 127.2, 126.9, 125.2, 118.7, 118.2, 101.4, 47.3, 29.8, 21.5, 20.3, 14.5. **IR:** ν_max_ (thin film): 2928, 2859, 2172, 1978, 1613, 1577, 1472 1416, 1336, 774, 729, 699. **HRMS** (ES+): found 429.2352; C_27_H_27_N_2_ [M+H]^+^ requires 429.2325. **HPLC**: CHIRALART Cellulose-SB, 40% MeCN/H_2_O, 0.75 mL/min, λ = 254 nm, t_R_ (major) = 6.3 min, t_R_ (minor) = 7.1 min. [α]_D_^25^ +24.7 (c = 0.05, CHCl_3_, 83 % *ee*).

**(R)-1-(2-isopropylphenyl)-4-((3-methylbenzyl)amino)-2-phenylquinolin-1-ium chloride (5k)**

Prepared according to **General Procedure D**, using quinolin-4-one **2b** (33 mg, 0.1 mmol, 1.0 equiv., 69 % *ee*), (COCl)_2_ (43 μL, 0.5 mmol, 5.0 equiv.), DCM (1 mL), 3-methylbenzylamine (63 μL, 0.5 mmol, 5.0 equiv.), MeCN (1 mL). Crude mixture purified by flash column chromatography (silica gel, 5% MeOH/DCM) to afford **3k** as an orange foam (42 mg, 88 %, 66 % *ee*).

**1H NMR** (400 MHz, CDCl_3_): δ_H_ = 12.13-12.02 (1H, m), 9.68 (1H, d, *J* = 8.3 Hz), 7.75 (1H, t, *J* = 7.8 Hz), 7.65 (1H, t, *J* = 7.8 Hz), 7.44 (1H, t, *J* = 7.6 Hz), 7.37-7.27 (6H, m), 7.21 (3H, q, *J* = 8.0 Hz), 7.07 (1H, d, *J* = 7.4 Hz), 7.02 (2H, d, *J* = 7.4 Hz), 6.94 (1H, d, *J* = 8.7 Hz), 6.57 (1H, s), 5.00-4.94 (1H, m), 4.91-4.84 (1H, m), 2.30 (3H, s), 2.10 (1H, sept, *J* = 6.8 Hz), 0.74 (6H, dd, *J* = 8.4, 6.8 Hz). **^13^C NMR** (101 MHz, CDCl_3_): δ_C_ = 156.0, 155.6, 146.0, 140.4, 138.5, 136.1, 134.9, 133.75, 133.72, 131.2, 130.2, 130.3, 129.4, 128.7, 128.6, 128.4, 128.2, 128.1, 127.6, 127.2, 126.9, 125.1, 119.1, 118.1, 101.3, 59.5, 47.2, 38.1, 31.2, 29.7, 27.9, 24.9, 21.9, 21.4, 14.1. **IR:** ν_max_ (thin film): 2962, 2859, 2186, 2014, 1604, 1577, 1467, 1336, 1078, 904, 724, 693, 644. **HRMS** (ES+): found 443.2506; C_32_H_30_N_2_ [M+H]^+^ requires 443.2482 **HPLC**: CHIRALART Cellulose-SB, 45% MeCN/H_2_O, 0.75 mL/min, λ = 254 nm, t_R_ (major) = 13.1 min, t_R_ (minor) = 15.4 min. [α]_D_^25^ -37.2 (c = 0.05, CHCl_3_, 66 % *ee*).

**(R)-4-((3-methylbenzyl)amino)-1-(naphthalen-1-yl)-2-phenylquinolin-1-ium chloride (5l)**

Prepared according to **General Procedure D**, using quinolin-4-one **2c** (33 mg, 0.1 mmol, 1.0 equiv., 73 % *ee*), (COCl)_2_ (43 μL, 0.5 mmol, 5.0 equiv.), DCM (1 mL), 3-methylbenzylamine (63 μL, 0.5 mmol, 5.0 equiv.), MeCN (1 mL). Crude mixture purified by flash column chromatography (silica gel, 5% MeOH/DCM) to afford **3l** as an orange foam (42 mg, 86 %, 71 % *ee*).

**1H NMR** (400 MHz, CDCl_3_): δ_H_ = 12.29 (1H, m), 9.73 (1H, d, *J* = 8.3 Hz), 7.93-7.87 (2H, m), 7.73 (1H, t, *J* = 7.8 Hz), 7.57 (2H, m), 7.48 (1H, t, *J* = 7.7 Hz), 7.42 (1H, t, *J* = 7.4 Hz), 7.38 (1H, s), 7.33 (1H, d, *J* = 7.7 Hz), 7.27 (1H, d, *J* = 7.0 Hz), 7.20 (1H, t, *J* = 7.7 Hz), 7.17-7.12 (2H, m), 7.07 (1H, d, *J* = 7.4 Hz), 7.01 (2H, t, *J* = 7.7 Hz), 6.88 (2H, d, *J* = 7.5 Hz), 6.81 (1H, d, *J* = 8.7 Hz), 6.63 (1H, s), 5.06-5.98 (1H, m), 4.91-4.83 (1H, m) 2.31 (3H, s). **^13^C NMR** (101 MHz, CDCl_3_): δ_C_ = 155.5, 155.1, 139.3, 137.5, 135.0, 133.1, 133.0, 132.92, 132.94, 129.9, 129.2, 129.0, 127.9, 127.7, 127.6, 127.5, 127.1, 127.0, 126.6, 126.4, 126.2, 124.2, 124.1, 120.7, 118.1, 117.0, 100.4, 52.4, 46.3, 20.4. . **IR:** ν_max_ (thin film): 2931, 2130, 1997, 1604, 1574, 1421,1330, 780, 760, 699. **HRMS** (ES+): found 451.2234; C_33_H_27_N_2_ [M+H]^+^ requires 451.2169 **HPLC**: CHIRALPAK OD-RH, 60% MeCN/H_2_O, 0.75 mL/min, λ = 254 nm, t_R_ (major) = 11.4 min, t_R_ (minor) = 17.7 min. [α]_D_^25^ +79.0 (c = 0.1, CHCl_3_, 71 % *ee*).

**(R)-1-(2,3-dimethylphenyl)-4-((2-hydroxyethyl)amino)-2-phenylquinolin-1-ium chloride (5m)**

Prepared according to **General Procedure C**, using quinolin-4-one **2a** (33 mg, 0.1 mmol, 1.0 equiv., 83 % *ee*), (COCl)_2_ (43 μL, 0.5 mmol, 5.0 equiv.), DCM (1 mL), ethanolamine (30 μL, 0.5 mmol, 5.0 equiv.), MeCN (1 mL). Crude mixture purified by flash column chromatography (silica gel, 5 % MeOH/DCM) to afford **3m** as an orange foam (40 mg, 99 %).

**1H NMR** (400 MHz, CDCl_3_): δ_H_ = 10.60, (1H, bs), 9.76 (1H, d, *J* = 8.5 Hz), 7.78 (1H, t, *J* = 7.4 Hz), 7.68 (1H, t, *J* = 8.0 Hz), 7.34 (1H, t, *J* = 7.4 Hz), 7.31-7.27 (2H, m), 7.24-7.15 (4H, m), 7.03 (1H, d, *J* = 7.9 Hz), 6.96 (1H, d, *J* = 8.6 Hz), 6.59 (1H, s), 5.55 (1H, bs), 4.29-4.17 (2H, m), 3.70-3.60 (2H, m), 2.24 (3H, s), 1.75 (3H, s). **^13^C NMR** (101 MHz, CDCl_3_): δ_C_ = 156.6, 156.3, 139.7, 136.9, 134.4, 134.3, 134.1, 131.9, 130.3, 128.6, 128.5, 127.8, 127.4, 127.3, 126.9, 118.8, 117.9, 100.7, 58.6, 48.9, 29.8, 20.4, 14.5. **IR:** ν_max_ (thin film): 2922, 2856, 2025, 1967, 1580,1610, 1472, 1350, 1068, 765. **HRMS** (ES+): found 369.1980; C_25_H_25_N_2_O [M+H]^+^ requires 369.1961.

**(R)-4-((2-hydroxyethyl)amino)-1-(2-isopropylphenyl)-2-phenylquinolin-1-ium chloride (5o)**

Prepared according to **General Procedure C**, using quinolin-4-one **2b** (33 mg, 0.1 mmol, 1.0 equiv., 69 % *ee*), (COCl)_2_ (43 μL, 0.5 mmol, 5.0 equiv.), DCM (1 mL), ethanolamine (30 μL, 0.5 mmol, 5.0 equiv.), MeCN (1 mL). Crude mixture purified by flash column chromatography (silica gel, 5% MeOH/DCM) to afford **3n** as an orange foam (41 mg, 98 %).

**1H NMR** (400 MHz, CDCl_3_): δ_H_ = 9.64 (1H, bs), 7.76-7.69 (1H, m), 7.66 (1H, t, *J* = 8.3 Hz), 7.48-7.42 (1H, m), 7.39-7.31 (4H, m), 7.30-7.26 (3H, m), 7.25-7.22 (2H, m), 6.97 (1H, d, *J* = 8.6 Hz), 6.64 (1H, s), 4.24-4.11 (2H, m), 3.70 (2H, bs), 2.20 (1H, sept, *J* = 6.8 Hz), 0.82 (3H, d, *J* = 6.8 Hz), 0.77 (3H, d, *J* = 6.8 Hz). **^13^C NMR** (101 MHz, CDCl_3_): δ_C_ = 154.5, 154.3, 144.0, 138.3, 133.1, 132.0, 131.8, 129.3, 128.4, 128.1, 127.5, 126.4, 126.2, 125.7, 125.2, 125.0, 117.3, 115.9, 98.8, 56.7, 46.9, 26.1, 23.1, 20.1. **IR:** ν_max_ (thin film): 2926, 2856, 2172, 2047, 1590, 1552, 1427, 1015, 804, 771. **HRMS** (ES+): found 383.2119; C_26_H_27_N_2_O [M+H]^+^ requires 383.2118.

**(R)-4-((2-hydroxyethyl)amino)-1-(naphthalen-1-yl)-2-phenylquinolin-1-ium chloride (5p)**

Prepared according to **General Procedure C**, using quinolin-4-one **2c** (33 mg, 0.1 mmol, 1.0 equiv., 73 % *ee*), (COCl)_2_ (43 μL, 0.5 mmol, 5.0 equiv.), DCM (1 mL), ethanolamine (30 μL, 0.5 mmol, 5.0 equiv.), MeCN (1 mL). Crude mixture purified by flash column chromatography (silica gel, 5% MeOH/DCM) to afford **3o** as an orange foam (40 mg, 95 %, 75 % *ee*).

**1H NMR** (400 MHz, CDCl_3_): δ_H_ = 9.76 (1H, d, *J* = 8.4 Hz), 7.92 (2H, t, *J* = 8.8 Hz), 7.69 (1H, t, *J* = 7.7 Hz), 7.58-7.42 (6H, m), 7.18 (2H, t, *J* = 8.6 Hz), 7.12 (2H, d, *J* = 7.1 Hz), 7.08 (2H, t, *J* = 7.6 Hz), 6.86 (1H, d, *J* = 8.8 Hz), 6.70 (1H, s), 4.24-4.18 (2H, m), 3.72-3.65 (2H, m). **^13^C NMR** (101 MHz, CDCl_3_): δ_C_ = 156.8, 140.4, 134.4, 134.2, 131.0, 130.4, 130.1, 129.0, 128.9, 128.2, 127.6, 127.0, 124.4, 122.0, 119.2, 101.0, 49.0, 29.8. . **IR:** ν_max_ (thin film): 3507, 2897, 2856, 2166, 2022, 1607, 1574, 1347, 1073, 920, 726. **HRMS** (ES+): found 391.1893; C_27_H_27_N_2_ [M+H]^+^ requires 391.1805. **HPLC**: CHIRALPAK OD-RH, 30% MeCN/H_2_O, 0.75 mL/min, λ = 254 nm, t_R_ (major) = 21.5 min, t_R_ (minor) = 26.6 min. [α]_D_^25^ +129.3 (c = 0.1, CHCl_3_, 75 % *ee*).

## 2.4 Synthesis of Axially Chiral Imine

**N-(3-methylbenzyl)-1-(naphthalen-1-yl)-2-phenylquinolin-4(1H)-imine (6)**

Prepared based on the literature procedure.^[11]^ Quinolinium salt **5l** (25 mg, 0.05 mmol, 71% *ee*) was dissolved in DCM (1 mL). In a separating funnel, this solution was shaken with aqueous 1.0 M NaOH (3 x 1 mL) and the organic layer was separated, dried over anhydrous MgSO_4_, filtered and concentrated *in vacuo* to yield the desired imine **6** as an orange solid (22 mg, quantitative).

**1H NMR** (600 MHz, CDCl_3_): δ_H_ = 8.73 (1H, d, *J* = 7.9 Hz), 7.83 (1H, d, *J* = 7.7 Hz), 7.78 (1H, d, *J* = 8.1 Hz), 7.67 (1H, d, *J* = 7.9 Hz), 7.48 (2H, quin, *J* = 6.8 Hz), 7.41-7.29 (4H, m), 7.22 (1H, t, *J* = 7.6 Hz), 7.15 (1H, t, *J* = 7.8 Hz), 7.09-7.01 (4H, m), 6.97 (2H, t, *J* = 7.3 Hz), 6.45 (1H, d, *J* = 8.5 Hz), 6.26 (1H, s), 4.73 (2H, m), 2.40 (3H, s). **^13^C NMR** (151 MHz, CDCl_3_): 155.1, 150.0, 142.4, 141.4, 137.9, 136.9, 136.6, 134.4, 132.0, 130.0, 129.3, 129.2, 128.72, 128.70, 128.5, 128.3, 128.2, 127.6, 127.5, 126.9, 126.8, 125.4, 125.2, 125.04, 124.99, 123.5, 123.4, 117.5, 101.9, 53.9, 29.9, 21.7. **IR:** ν_max_ (thin film): 2928, 2848, 2197, 2025, 1621, 1560, 1460, 1394, 1327, 901, 718, 649. **HRMS** (ES+): found 451.2179; C_27_H_27_N_2_ [M+H]^+^ requires 451.2169.

Resolution of the enantiomers of **6** was not achieved by chiral HPLC. However, hydrolysis of **6** under aqueous acidic conditions (1 M HCl) led to recovery of the corresponding quinolin-4-one **2b** with no loss in *ee* (73 %).

**Figure S1.** Comparison of the ^1^H NMR spectra (CDCl_3_, 600 MHz) of quinolinium salt **5l** (in blue, bottom) and axially chiral imine **6** (in red, top).

# 3. Unsuccessful Reactions

## 3.1 Atroposelective Synthesis of N-aryl-quinolin-4-ones

Synthesis of N-aryl-quinolin-4-ones **2d** and **2e** was attempted according to **General Procedure B** but were unsuccessful as no product formation was observed by ^1^H NMR and LC-MS analysis of the crude materials. In the case of **2d,** we theorize that the low temperature conditions are not sufficient to overcome the reactivity barrier to accommodate the much more sterically hindered *ortho*‑*tert‑*butyl substituent.

As for **2e**, the predicted product should possess a much lower rotational barrier^[12]^ and therefore should not require high temperatures to overcome any kinetic barriers. However, we postulate that it may be possible that coordination of the methoxy oxygen and enaminone carbonyl C=O to the palladium centre may hinder reactivity by preventing N-Pd coordination.^[13]^

## 3.2 Formation of Quinolinium Salts

We found a small number of compounds which proved to be unsuitable to undergo S_N_Ar with chloroquinolinium intermediate **4**. Firstly, compounds which are weakly nucleophilic were unable carry out substitution of **4**, including carbazole and benzyl alcohol. These reactions were carried out at 80 °C and LC-MS analysis showed no product formation. Interestingly, dimethyl aniline was mixed with **4** under the same conditions and no product formation was observed, despite the known nucleophilicity of aniline compounds. This result may simply be down to product stability, undergoing hydrolysis back to the starting quinolone **2a**. In addition, we also subjected **4** to the reaction with glycine which also saw no product formation.

Furthermore, product stability proved a major factor in the isolation of the quinolinium salts. Salts **3p** and **3q** were synthesized according to **General Procedure C**. Product formation was observed by TLC but any attempt at purification by column chromatography, acid/base washing followed by extraction and recrystallization resulted in product decomposition back to **2a**. It is worth noting that **3p** displayed visible solvatochromism as a crude mixture and if isolated, these properties could be worth investigating further.

## 3.3 Synthesis of Quinolinimines

When quinolinium salt **5p** was reacted under the same conditions as described for synthesis of quinolinimine **6**, product decomposition was observed by ^1^H NMR. It is currently unclear what caused this decomposition as both ^1^H NMR and LC-MS analysis provided unclear results.

# 4. Density Functional Theory Calculations

## 4.1 Methods

All optimisation, frequency, and single-point calculations were performed with *Gaussian 16*, rev C.01.^[14]^ The B3LYP functional was used for all geometry optimisations with the 6-31G(d,p) basis set on all atoms. Grimme’s DFT-D3BJ correction was included in the optimisation procedure as well as single point calculations. All optimised structures were confirmed as minima by the absence of imaginary frequencies while transition states were characterised by the presence of a single imaginary frequency. Further refinements to the electronic energies were made through single point calculations on the optimised geometries using the B3LYP functional with the 6-311+G(d,p) basis set for all atoms. Free energies were determined from thermochemical corrections of the geometries applied to electronic energies.

Supporting data are openly available from Queen's Research Portal (Data Repository) under the DOI: [10.17034/daa2c78b-9801-44c8-a35c-f772ba19ac5a](https://eur02.safelinks.protection.outlook.com/?url=https%3A%2F%2Fdoi.org%2F10.17034%2Fdaa2c78b-9801-44c8-a35c-f772ba19ac5a&data=05%7C02%7CP.Knipe%40qub.ac.uk%7C8ecf9271943f4a3612ed08dec30522c5%7Ceaab77eab4a549e3a1e8d6dd23a1f286%7C0%7C0%7C639162623994230451%7CUnknown%7CTWFpbGZsb3d8eyJFbXB0eU1hcGkiOnRydWUsIlYiOiIwLjAuMDAwMCIsIlAiOiJXaW4zMiIsIkFOIjoiTWFpbCIsIldUIjoyfQ%3D%3D%7C0%7C%7C%7C&sdata=E4URRiErgAX5uHCetW84Szdbt9O89bu64nxBvWN3W0c%3D&reserved=0)

## 4.2 Basis Set comparison

Different basis sets for single point calculations were compared (Table S2). Geometry optimisation and frequency calculations remained at the same level of theory (B3LYP/6-31G(d,p)/GD3BJ). Very little difference in barrier energies are observed, providing confidence in our calculations.

Table S2: Single point basis set comparison

| **Compound** | **6-311+G(d,p)** | **def2TZVP** | **def2TZVPP** | **aug-cc-pVTZ** |
| --- | --- | --- | --- | --- |
| **5b** | 36.4 | 36.5 | 36.5 | 36.5 |
| **5c** | 36.0 | 36.1 | 36.1 | 36.0 |
| **5i** | 32.2 | 32.2 | 32.2 | 32.1 |
| **6** | 26.2 | 26.5 | 26.5 | 26.6 |

## 4.3 Coordinates

### 5b (Ground State)

| C | 1.46353 | -1.70644 | 2.92836 |
| --- | --- | --- | --- |
| C | 1.78441 | -0.46251 | 2.07884 |
| C | 2.48287 | -0.87406 | 0.79367 |
| C | 3.8316 | -1.25952 | 0.79946 |
| C | 4.47703 | -1.68899 | -0.35653 |
| C | 3.78489 | -1.75264 | -1.5677 |
| C | 2.44739 | -1.37519 | -1.60521 |
| C | 1.82046 | -0.93651 | -0.438 |
| N | 0.41902 | -0.55768 | -0.52327 |
| C | 0.06511 | 0.75658 | -0.57478 |
| C | 1.10907 | 1.80852 | -0.60078 |
| C | 1.09109 | 2.78783 | 0.40325 |
| C | 2.04458 | 3.80399 | 0.41096 |
| C | 3.01058 | 3.86053 | -0.59354 |
| C | 3.01855 | 2.90101 | -1.60794 |
| C | 2.07645 | 1.87704 | -1.61317 |
| C | -1.26542 | 1.12408 | -0.52824 |
| C | -2.31436 | 0.18787 | -0.38281 |
| N | -3.56664 | 0.63722 | -0.17664 |
| C | -4.72098 | -0.1219 | 0.36034 |
| C | -5.59049 | 0.95282 | 1.01591 |
| C | -5.36775 | 2.16504 | 0.10645 |
| C | -3.87066 | 2.08923 | -0.19294 |
| C | -1.94382 | -1.21541 | -0.47479 |
| C | -0.55816 | -1.55494 | -0.48775 |
| C | -0.16313 | -2.90839 | -0.47691 |
| C | -1.10799 | -3.91274 | -0.53536 |
| C | -2.47146 | -3.5963 | -0.6322 |
| C | -2.87356 | -2.2774 | -0.59877 |
| C | 2.59303 | 0.56998 | 2.87835 |
| H | 2.38212 | -2.22218 | 3.22498 |
| H | 0.92846 | -1.42045 | 3.83888 |
| H | 0.84274 | -2.41743 | 2.37495 |
| H | 0.83146 | 0.00576 | 1.81394 |
| H | 4.38375 | -1.22509 | 1.73213 |
| H | 5.52154 | -1.97906 | -0.31254 |
| H | 4.28063 | -2.09047 | -2.47103 |
| H | 1.88026 | -1.41676 | -2.52967 |
| H | 0.34651 | 2.73356 | 1.19073 |
| H | 2.03257 | 4.54761 | 1.20072 |
| H | 3.75261 | 4.652 | -0.5896 |
| H | 3.75925 | 2.95111 | -2.39884 |
| H | 2.08531 | 1.14103 | -2.40697 |
| H | -1.47022 | 2.18157 | -0.56857 |
| H | -5.27091 | -0.60493 | -0.45539 |
| H | -4.37911 | -0.88753 | 1.05833 |
| H | -6.63514 | 0.64368 | 1.08277 |
| H | -5.23092 | 1.16174 | 2.0287 |
| H | -5.64098 | 3.11374 | 0.57136 |
| H | -5.94549 | 2.06131 | -0.81743 |
| H | -3.59968 | 2.52089 | -1.16015 |
| H | -3.28731 | 2.59437 | 0.58722 |
| H | 0.88867 | -3.15313 | -0.44158 |
| H | -0.78645 | -4.94855 | -0.53169 |
| H | -3.2102 | -4.38305 | -0.73474 |
| H | -3.92351 | -2.06061 | -0.70398 |
| H | 3.52528 | 0.14469 | 3.26119 |
| H | 2.0139 | 0.91032 | 3.74182 |
| H | 2.84105 | 1.43971 | 2.26485 |

### 5b (Transition State)

| C | 3.1709 | -3.38061 | -0.6816 |
| --- | --- | --- | --- |
| C | 2.12221 | -2.89243 | 0.33137 |
| C | 2.39737 | -1.46482 | 0.81117 |
| C | 3.53171 | -1.35092 | 1.63745 |
| C | 3.84803 | -0.22359 | 2.37792 |
| C | 2.95227 | 0.8409 | 2.36388 |
| C | 1.85039 | 0.78733 | 1.52759 |
| C | 1.60573 | -0.2945 | 0.65355 |
| N | 0.51456 | -0.0675 | -0.2986 |
| C | -0.04505 | 1.21108 | -0.38677 |
| C | 0.82587 | 2.37443 | -0.63032 |
| C | 0.46442 | 3.66062 | -0.19782 |
| C | 1.26177 | 4.75643 | -0.51178 |
| C | 2.42693 | 4.58241 | -1.26122 |
| C | 2.79423 | 3.30572 | -1.69265 |
| C | 2.00472 | 2.20656 | -1.37614 |
| C | -1.41367 | 1.34619 | -0.32843 |
| C | -2.24949 | 0.19725 | -0.21194 |
| N | -3.47132 | 0.28406 | 0.30041 |
| C | -4.36538 | -0.84482 | 0.64498 |
| C | -5.15207 | -0.30947 | 1.84238 |
| C | -5.33687 | 1.17256 | 1.48874 |
| C | -3.99701 | 1.55804 | 0.85106 |
| C | -1.68497 | -1.00681 | -0.816 |
| C | -0.27274 | -1.06144 | -0.9363 |
| C | 0.30594 | -1.97103 | -1.82498 |
| C | -0.47929 | -2.89679 | -2.49932 |
| C | -1.86635 | -2.90905 | -2.32016 |
| C | -2.46294 | -1.95636 | -1.51015 |
| C | 2.02231 | -3.8625 | 1.52615 |
| H | 4.16059 | -3.44787 | -0.22051 |
| H | 2.91101 | -4.376 | -1.05429 |
| H | 3.26457 | -2.70659 | -1.53925 |
| H | 1.14736 | -2.9375 | -0.13769 |
| H | 4.17319 | -2.22039 | 1.72442 |
| H | 4.73893 | -0.20259 | 2.99583 |
| H | 3.09577 | 1.70644 | 3.00148 |
| H | 1.16175 | 1.61606 | 1.56134 |
| H | -0.41949 | 3.79832 | 0.41655 |
| H | 0.98005 | 5.74464 | -0.16423 |
| H | 3.04863 | 5.43762 | -1.50421 |
| H | 3.69703 | 3.16789 | -2.27803 |
| H | 2.28668 | 1.21555 | -1.71225 |
| H | -1.84471 | 2.33384 | -0.40901 |
| H | -5.03801 | -1.05557 | -0.19401 |
| H | -3.77408 | -1.73651 | 0.85611 |
| H | -6.09549 | -0.84036 | 1.98029 |
| H | -4.56089 | -0.41863 | 2.75715 |
| H | -5.56679 | 1.79658 | 2.35371 |
| H | -6.14785 | 1.29132 | 0.76378 |
| H | -4.09952 | 2.2996 | 0.05377 |
| H | -3.28016 | 1.94003 | 1.58648 |
| H | 1.3689 | -1.91756 | -2.0127 |
| H | -0.01242 | -3.58533 | -3.19518 |
| H | -2.48151 | -3.61981 | -2.8603 |
| H | -3.54175 | -1.89718 | -1.47831 |
| H | 2.97541 | -3.98621 | 2.04652 |
| H | 1.7105 | -4.84973 | 1.17096 |
| H | 1.28575 | -3.51238 | 2.25484 |

### 5c (Ground State)

| C | 0.2995 | 0.9095 | 0.05112 |
| --- | --- | --- | --- |
| C | -0.55503 | 2.11873 | 0.12132 |
| C | -1.66978 | 2.18569 | 0.96917 |
| C | -2.42391 | 3.35355 | 1.03207 |
| C | -2.07976 | 4.45823 | 0.25052 |
| C | -0.96715 | 4.39861 | -0.58847 |
| C | -0.20106 | 3.23627 | -0.64765 |
| N | -0.23585 | -0.28487 | -0.32991 |
| C | -1.62179 | -0.35622 | -0.7572 |
| C | -1.93969 | 0.07049 | -2.02413 |
| C | -3.28187 | 0.03449 | -2.46199 |
| C | -4.26652 | -0.43267 | -1.62279 |
| C | -3.95985 | -0.88593 | -0.31289 |
| C | -2.60255 | -0.85368 | 0.1438 |
| C | -2.30331 | -1.30097 | 1.45714 |
| C | -3.30577 | -1.75691 | 2.2829 |
| C | -4.64804 | -1.79163 | 1.8357 |
| C | -4.96518 | -1.36637 | 0.56695 |
| C | 0.55139 | -1.43934 | -0.3573 |
| C | -0.04476 | -2.67216 | -0.68895 |
| C | 0.68567 | -3.8409 | -0.62104 |
| C | 2.02436 | -3.81511 | -0.20084 |
| C | 2.63259 | -2.60959 | 0.08042 |
| C | 1.9386 | -1.37939 | -0.03253 |
| C | 2.54056 | -0.0768 | 0.20519 |
| N | 3.86313 | 0.14383 | 0.3213 |
| C | 4.96671 | -0.74094 | -0.12162 |
| C | 6.1163 | 0.22712 | -0.40775 |
| C | 5.9066 | 1.3177 | 0.64733 |
| C | 4.38769 | 1.49108 | 0.6544 |
| C | 1.64837 | 1.01304 | 0.32775 |
| H | -1.93764 | 1.33673 | 1.58588 |
| H | -3.28047 | 3.40179 | 1.69606 |
| H | -2.67514 | 5.36396 | 0.29909 |
| H | -0.69562 | 5.2542 | -1.1977 |
| H | 0.65934 | 3.18261 | -1.30715 |
| H | -1.15759 | 0.443 | -2.67667 |
| H | -3.52627 | 0.37816 | -3.46077 |
| H | -5.30011 | -0.46094 | -1.95351 |
| H | -1.27717 | -1.28131 | 1.80769 |
| H | -3.06648 | -2.09508 | 3.28576 |
| H | -5.42525 | -2.15542 | 2.49918 |
| H | -5.99301 | -1.38962 | 0.21826 |
| H | -1.08567 | -2.69725 | -0.97767 |
| H | 0.20975 | -4.78279 | -0.87116 |
| H | 2.58258 | -4.73843 | -0.09527 |
| H | 3.6526 | -2.6193 | 0.42732 |
| H | 5.24672 | -1.43094 | 0.68255 |
| H | 4.66046 | -1.32039 | -0.99373 |
| H | 7.08783 | -0.26554 | -0.33861 |
| H | 6.01559 | 0.64343 | -1.41532 |
| H | 6.25419 | 0.974 | 1.6267 |
| H | 6.42037 | 2.25202 | 0.41515 |
| H | 4.06895 | 2.20743 | -0.11313 |
| H | 3.99405 | 1.82178 | 1.6193 |
| H | 2.00319 | 1.98986 | 0.61419 |

### 5c (Transition State)

| C | -0.36231 | 1.14006 | 0.50221 |
| --- | --- | --- | --- |
| C | 0.41368 | 2.36087 | 0.78011 |
| C | -0.04314 | 3.62276 | 0.36812 |
| C | 0.66505 | 4.77149 | 0.7065 |
| C | 1.83714 | 4.67469 | 1.45863 |
| C | 2.30029 | 3.42225 | 1.86907 |
| C | 1.59855 | 2.27171 | 1.53022 |
| N | 0.30329 | -0.08595 | 0.40082 |
| C | 1.39721 | -0.16219 | -0.55552 |
| C | 1.43604 | 0.8774 | -1.47595 |
| C | 2.57091 | 1.15317 | -2.25832 |
| C | 3.68435 | 0.36352 | -2.13828 |
| C | 3.63041 | -0.81601 | -1.35455 |
| C | 2.44452 | -1.15897 | -0.6067 |
| C | 2.38841 | -2.48384 | -0.09588 |
| C | 3.46337 | -3.34341 | -0.17026 |
| C | 4.67189 | -2.94176 | -0.77289 |
| C | 4.73432 | -1.70858 | -1.37409 |
| C | -0.41779 | -1.16833 | 0.97465 |
| C | 0.20572 | -2.0486 | 1.86194 |
| C | -0.51451 | -3.07446 | 2.46215 |
| C | -1.88214 | -3.21076 | 2.21328 |
| C | -2.53319 | -2.28265 | 1.41498 |
| C | -1.82693 | -1.2322 | 0.79546 |
| C | -2.4618 | -0.05661 | 0.19468 |
| N | -3.6445 | -0.0441 | -0.41096 |
| C | -4.50559 | -1.21837 | -0.7002 |
| C | -5.55925 | -0.69833 | -1.69363 |
| C | -4.93708 | 0.57238 | -2.28576 |
| C | -4.15889 | 1.15759 | -1.1082 |
| C | -1.73315 | 1.15639 | 0.39627 |
| H | -0.9331 | 3.7037 | -0.24771 |
| H | 0.30878 | 5.74106 | 0.37514 |
| H | 2.38985 | 5.57082 | 1.7202 |
| H | 3.20893 | 3.3437 | 2.45639 |
| H | 1.95501 | 1.29971 | 1.85112 |
| H | 0.58684 | 1.53824 | -1.57212 |
| H | 2.55661 | 2.00498 | -2.92909 |
| H | 4.59311 | 0.58653 | -2.68765 |
| H | 1.46538 | -2.87333 | 0.29461 |
| H | 3.36343 | -4.35106 | 0.22034 |
| H | 5.51951 | -3.61761 | -0.8068 |
| H | 5.62461 | -1.4039 | -1.91554 |
| H | 1.24818 | -1.8984 | 2.10537 |
| H | -0.0158 | -3.74229 | 3.15619 |
| H | -2.45011 | -4.00073 | 2.69154 |
| H | -3.60809 | -2.33443 | 1.33841 |
| H | -3.88353 | -2.02015 | -1.10851 |
| H | -4.97877 | -1.57777 | 0.21465 |
| H | -5.80243 | -1.44718 | -2.44903 |
| H | -6.48179 | -0.45333 | -1.15967 |
| H | -4.24457 | 0.3246 | -3.09619 |
| H | -5.68022 | 1.27063 | -2.67446 |
| H | -4.80663 | 1.72642 | -0.43044 |
| H | -3.3245 | 1.79058 | -1.41136 |
| H | -2.25874 | 2.09419 | 0.50715 |
|  |  |  |  |

### 5i (Ground State)

| C | 5.52752 | -1.40758 | 0.17703 |
| --- | --- | --- | --- |
| C | 4.24031 | -1.01223 | 0.90863 |
| C | 4.51484 | -0.65234 | 2.37442 |
| N | 3.50149 | 0.08866 | 0.22028 |
| C | 4.21188 | 1.40245 | 0.19083 |
| C | 4.22298 | 1.99363 | -1.22143 |
| C | 3.79488 | 2.42615 | 1.26748 |
| C | 2.20805 | -0.04351 | -0.16236 |
| C | 1.33056 | 1.0596 | -0.15921 |
| C | -0.03941 | 0.92714 | -0.29543 |
| C | -0.89689 | 2.12611 | -0.1457 |
| C | -1.90763 | 2.20084 | 0.82354 |
| C | -2.66077 | 3.36388 | 0.95151 |
| C | -2.41848 | 4.45637 | 0.11618 |
| C | -1.40786 | 4.38976 | -0.84297 |
| C | -0.64215 | 3.23249 | -0.96838 |
| N | -0.59838 | -0.2945 | -0.52488 |
| C | -2.03358 | -0.42732 | -0.68927 |
| C | -2.60631 | -0.02001 | -1.87 |
| C | -4.00331 | -0.1285 | -2.0467 |
| C | -4.7856 | -0.64991 | -1.04246 |
| C | -4.21374 | -1.08537 | 0.18188 |
| C | -2.79842 | -0.97686 | 0.37517 |
| C | -2.23247 | -1.40091 | 1.60553 |
| C | -3.03453 | -1.91103 | 2.60127 |
| C | -4.43254 | -2.02422 | 2.41347 |
| C | -5.00665 | -1.6205 | 1.23066 |
| C | 0.21553 | -1.40983 | -0.74082 |
| C | 1.629 | -1.30221 | -0.615 |
| C | 2.40548 | -2.40379 | -1.05098 |
| C | 1.82351 | -3.57788 | -1.47976 |
| C | 0.42444 | -3.69959 | -1.49183 |
| C | -0.37066 | -2.62644 | -1.14481 |
| H | 6.30042 | -0.63911 | 0.26567 |
| H | 5.93095 | -2.32209 | 0.61989 |
| H | 5.35981 | -1.58599 | -0.88822 |
| H | 3.56507 | -1.86505 | 0.92126 |
| H | 5.21445 | 0.18101 | 2.47677 |
| H | 4.96223 | -1.51735 | 2.87192 |
| H | 3.58953 | -0.40009 | 2.89862 |
| H | 5.24189 | 1.12765 | 0.41653 |
| H | 4.63213 | 1.27716 | -1.93795 |
| H | 3.22567 | 2.28486 | -1.55937 |
| H | 4.85133 | 2.88822 | -1.23292 |
| H | 3.46137 | 1.9409 | 2.1858 |
| H | 3.00696 | 3.10401 | 0.93088 |
| H | 4.65903 | 3.05001 | 1.5117 |
| H | 1.68382 | 2.03907 | 0.10543 |
| H | -2.0939 | 1.36182 | 1.4828 |
| H | -3.43625 | 3.41848 | 1.70818 |
| H | -3.01288 | 5.35847 | 0.21659 |
| H | -1.21557 | 5.23597 | -1.49401 |
| H | 0.13956 | 3.17371 | -1.71933 |
| H | -1.98216 | 0.3929 | -2.65488 |
| H | -4.44978 | 0.20102 | -2.97809 |
| H | -5.85983 | -0.73557 | -1.17355 |
| H | -1.1616 | -1.31968 | 1.75676 |

### 5i (Transition State)

| C | 5.17052 | -1.85549 | 0.38648 |
| --- | --- | --- | --- |
| C | 3.7745 | -1.56842 | 0.94267 |
| C | 3.71242 | -1.67797 | 2.47061 |
| N | 3.26175 | -0.23538 | 0.49399 |
| C | 4.02805 | 0.94183 | 1.01385 |
| C | 4.65345 | 1.76059 | -0.12047 |
| C | 3.25155 | 1.76738 | 2.04653 |
| C | 2.12542 | -0.11375 | -0.18715 |
| C | 1.44788 | 1.12916 | -0.40377 |
| C | 0.07881 | 1.14827 | -0.52419 |
| C | -0.66264 | 2.39973 | -0.76169 |
| C | -0.17015 | 3.63686 | -0.3164 |
| C | -0.84905 | 4.81376 | -0.61621 |
| C | -2.02825 | 4.77091 | -1.36198 |
| C | -2.52754 | 3.54379 | -1.80478 |
| C | -1.85481 | 2.36536 | -1.50466 |
| N | -0.62813 | -0.06072 | -0.46808 |
| C | -1.70911 | -0.15956 | 0.49537 |
| C | -1.74978 | 0.85372 | 1.44433 |
| C | -2.87356 | 1.08244 | 2.25752 |
| C | -3.97194 | 0.27124 | 2.1424 |
| C | -3.9145 | -0.87958 | 1.31767 |
| C | -2.74186 | -1.17127 | 0.52865 |
| C | -2.68072 | -2.46866 | -0.04861 |
| C | -3.73932 | -3.34885 | 0.01558 |
| C | -4.93532 | -2.99703 | 0.67226 |
| C | -5.00177 | -1.79257 | 1.32851 |
| C | 0.0697 | -1.12767 | -1.09736 |
| C | 1.46845 | -1.22131 | -0.90085 |
| C | 2.19258 | -2.17697 | -1.6427 |
| C | 1.55154 | -3.01929 | -2.5378 |
| C | 0.17785 | -2.88306 | -2.75814 |
| C | -0.55077 | -1.92581 | -2.06274 |
| H | 5.92855 | -1.19209 | 0.81189 |
| H | 5.45448 | -2.87968 | 0.64147 |
| H | 5.20415 | -1.75194 | -0.70129 |
| H | 3.07883 | -2.30385 | 0.5475 |
| H | 4.40955 | -1.00408 | 2.97484 |
| H | 3.98146 | -2.69815 | 2.75671 |
| H | 2.70223 | -1.47852 | 2.83689 |
| H | 4.85997 | 0.48814 | 1.55093 |
| H | 5.23161 | 1.11604 | -0.78695 |
| H | 3.92289 | 2.3018 | -0.72373 |
| H | 5.33384 | 2.49895 | 0.3118 |
| H | 2.82872 | 1.12406 | 2.82126 |
| H | 2.44145 | 2.35209 | 1.61153 |
| H | 3.94535 | 2.46334 | 2.52543 |
| H | 1.98171 | 2.06229 | -0.46112 |
| H | 0.72588 | 3.67916 | 0.29436 |
| H | -0.46428 | 5.76319 | -0.25928 |
| H | -2.55815 | 5.68886 | -1.59339 |
| H | -3.44207 | 3.5065 | -2.38706 |
| H | -2.23971 | 1.41307 | -1.85047 |
| H | -0.91235 | 1.52861 | 1.544 |
| H | -2.86223 | 1.91519 | 2.95202 |
| H | -4.87062 | 0.4569 | 2.72144 |
| H | -1.76622 | -2.82173 | -0.4903 |
| H | -3.63642 | -4.33401 | -0.42807 |
| H | -5.77041 | -3.68867 | 0.6992 |
| H | -5.88269 | -1.52678 | 1.90471 |
| H | 3.27184 | -2.20599 | -1.57321 |
| H | 2.12872 | -3.7385 | -3.10801 |
| H | -0.31632 | -3.48722 | -3.5112 |
| H | -1.59502 | -1.76088 | -2.29068 |
|  |  |  |  |

### 6 (Ground State)

| C | -7.6922 | -1.77874 | -0.51106 |
| --- | --- | --- | --- |
| C | -7.05185 | -0.48849 | -0.05923 |
| C | -7.82753 | 0.57462 | 0.41161 |
| C | -7.21978 | 1.75168 | 0.85251 |
| C | -5.83375 | 1.876 | 0.82439 |
| C | -5.03896 | 0.8247 | 0.35114 |
| C | -3.53296 | 0.99518 | 0.28521 |
| N | -2.83964 | -0.26005 | 0.08958 |
| C | -1.55286 | -0.24153 | -0.03991 |
| C | -0.83684 | -1.51862 | -0.23904 |
| C | 0.56276 | -1.5454 | -0.4185 |
| C | 1.21189 | -2.77113 | -0.6572 |
| C | 0.48335 | -3.95012 | -0.69063 |
| C | -0.90296 | -3.93618 | -0.49136 |
| C | -1.54625 | -2.72928 | -0.27261 |
| N | 1.30295 | -0.34732 | -0.36711 |
| C | 2.7059 | -0.37111 | -0.67859 |
| C | 3.12959 | 0.02607 | -1.92539 |
| C | 4.50643 | 0.03987 | -2.24172 |
| C | 5.43431 | -0.35629 | -1.30748 |
| C | 5.02914 | -0.7815 | -0.01516 |
| C | 3.63614 | -0.79189 | 0.31488 |
| C | 3.23632 | -1.20206 | 1.61362 |
| C | 4.17455 | -1.58563 | 2.54429 |
| C | 5.55259 | -1.58074 | 2.22069 |
| C | 5.96767 | -1.18795 | 0.96977 |
| C | 0.65319 | 0.86266 | -0.16352 |
| C | 1.47058 | 2.10339 | -0.10779 |
| C | 2.48747 | 2.27629 | 0.84184 |
| C | 3.21192 | 3.4641 | 0.8864 |
| C | 2.93116 | 4.49496 | -0.01259 |
| C | 1.914 | 4.33463 | -0.95275 |
| C | 1.1863 | 3.14608 | -0.99865 |
| C | -0.69822 | 0.92831 | -0.00734 |
| C | -5.65835 | -0.34552 | -0.08537 |
| H | -7.22498 | -2.15623 | -1.42626 |
| H | -8.76113 | -1.64981 | -0.70346 |
| H | -7.58601 | -2.56122 | 0.25033 |
| H | -8.91015 | 0.48081 | 0.43565 |
| H | -7.83084 | 2.57134 | 1.21991 |
| H | -5.36264 | 2.79311 | 1.17088 |
| H | -3.30576 | 1.70593 | -0.52971 |
| H | -3.19901 | 1.49792 | 1.20894 |
| H | 2.28173 | -2.79257 | -0.81541 |
| H | 1.00072 | -4.88692 | -0.87324 |
| H | -1.46854 | -4.86212 | -0.51345 |
| H | -2.61787 | -2.67155 | -0.12249 |
| H | 2.39326 | 0.34095 | -2.65652 |
| H | 4.82361 | 0.36466 | -3.2271 |
| H | 6.4941 | -0.3477 | -1.54489 |
| H | 2.18051 | -1.21166 | 1.85871 |
| H | 3.8569 | -1.89689 | 3.53437 |
| H | 6.28033 | -1.88795 | 2.96504 |
| H | 7.0238 | -1.17973 | 0.71588 |
| H | 2.70509 | 1.48455 | 1.54886 |
| H | 3.99477 | 3.58664 | 1.62842 |
| H | 3.50033 | 5.41869 | 0.02292 |
| H | 1.68772 | 5.132 | -1.65384 |
| H | 0.39876 | 3.01163 | -1.733 |
| H | -1.12604 | 1.90277 | 0.18187 |
| H | -5.02853 | -1.15319 | -0.44236 |
|  |  |  |  |

### 6 (Transition State)

| C | -6.77198 | 1.3036 | 1.14769 |
| --- | --- | --- | --- |
| C | -6.10032 | -0.03922 | 0.99345 |
| C | -6.64761 | -1.19078 | 1.56415 |
| C | -6.00302 | -2.42256 | 1.43019 |
| C | -4.80782 | -2.51295 | 0.72424 |
| C | -4.24612 | -1.37017 | 0.14088 |
| C | -2.94507 | -1.49844 | -0.6315 |
| N | -2.45783 | -0.22174 | -1.11436 |
| C | -1.19563 | -0.04691 | -1.25867 |
| C | -0.68263 | 1.23507 | -1.80267 |
| C | 0.66671 | 1.54446 | -1.58105 |
| C | 1.24443 | 2.65697 | -2.19457 |
| C | 0.46405 | 3.48125 | -3.00166 |
| C | -0.89233 | 3.19796 | -3.19855 |
| C | -1.46015 | 2.07327 | -2.60908 |
| N | 1.43779 | 0.70689 | -0.7016 |
| C | 1.72266 | 1.26179 | 0.60292 |
| C | 1.2899 | 2.56075 | 0.84973 |
| C | 1.62737 | 3.27027 | 2.01635 |
| C | 2.40058 | 2.68913 | 2.98474 |
| C | 2.80461 | 1.34119 | 2.83343 |
| C | 2.44896 | 0.58271 | 1.65701 |
| C | 2.81548 | -0.7901 | 1.66551 |
| C | 3.52098 | -1.3628 | 2.70003 |
| C | 3.91393 | -0.59752 | 3.81601 |
| C | 3.54881 | 0.72433 | 3.87424 |
| C | 1.15951 | -0.68234 | -0.94275 |
| C | 2.30102 | -1.57448 | -1.23772 |
| C | 3.52548 | -1.02042 | -1.64035 |
| C | 4.59808 | -1.84304 | -1.97406 |
| C | 4.47031 | -3.23021 | -1.90345 |
| C | 3.25802 | -3.79112 | -1.49432 |
| C | 2.18292 | -2.97275 | -1.16441 |
| C | -0.12667 | -1.05388 | -1.13281 |
| C | -4.8976 | -0.14649 | 0.28105 |
| H | -7.77893 | 1.20222 | 1.56164 |
| H | -6.85086 | 1.82189 | 0.18634 |
| H | -6.20092 | 1.95665 | 1.81828 |
| H | -7.58083 | -1.12478 | 2.11705 |
| H | -6.43652 | -3.31173 | 1.87876 |
| H | -4.30652 | -3.47282 | 0.62333 |
| H | -2.20908 | -2.02087 | -0.00116 |
| H | -3.12174 | -2.15092 | -1.50258 |
| H | 2.29095 | 2.87662 | -2.01309 |
| H | 0.91566 | 4.34262 | -3.48364 |
| H | -1.49587 | 3.84381 | -3.82826 |
| H | -2.4998 | 1.80915 | -2.76843 |
| H | 0.67666 | 3.06873 | 0.12251 |
| H | 1.26503 | 4.28735 | 2.12914 |
| H | 2.68844 | 3.22899 | 3.88139 |
| H | 2.53091 | -1.431 | 0.85577 |
| H | 3.76906 | -2.41845 | 2.64816 |
| H | 4.47905 | -1.05199 | 4.62382 |
| H | 3.81133 | 1.33168 | 4.73589 |
| H | 3.61876 | 0.05808 | -1.68352 |
| H | 5.53774 | -1.39834 | -2.28668 |
| H | 5.30944 | -3.87054 | -2.1563 |
| H | 3.15365 | -4.86933 | -1.42301 |
| H | 1.25775 | -3.41777 | -0.81299 |
| H | -0.35349 | -2.07465 | -1.41867 |
| H | -4.45081 | 0.72984 | -0.17591 |

# 5. HPLC Data

## 5.1 Quinolin-4-ones (2)

**2a**

**Conditions**: CHIRALART Cellulose‑SB, 15 % IPA/Hex, 1 mL/min, λ=240 nm, t_R_ (major) = 11.4 min, t_R_ (minor) = 13.2 min.

**Racemic trace**

**Asymmetric – 92 % ee**

**Asymmetric – 83 % ee**

**2b**

**Conditions**: CHIRALART Cellulose‑SB, 15 % IPA/Hex, 1 mL/min, λ=240 nm, t_R_ (major) = 10.9 min, t_R_ (minor) = 14.6 min.

**Racemic trace**

**Asymmetric – 85 % ee**

**Asymmetric – 69 % ee**

**2c**

**Conditions**: CHIRALPAK OD-RH, 60 % MeCN/H_2_O, 0.75 mL/min, λ = 254 nm, t_R1_ = 15.1 min, t_R2_ = 18.2 min.

**Racemic trace**

**Asymmetric – 91 % ee**

**Asymmetric – 73 % ee**

## 5.2 4-amino-quinolinium salts (5)

**5a**

**Racemic trace**

**Asymmetric – 85 % ee**

**5b**

**Racemic trace**

**Asymmetric – 85 % ee**

**5c**

**Racemic trace**

**Asymmetric – 76 % ee**

**5d**

**Racemic Trace**

**Asymmetric – 85 % ee**

**5e**

**Racemic Trace**

**Asymmetric – 69 % ee**

**5f**

**Racemic trace**

**Asymmetric – 75 % ee**

**5j**

**Racemic trace**

**Asymmetric – 83 % ee**

**5k**

**Racemic trace**

**Asymmetric – 66 % ee**

**5l**

**Racemic trace**

**Asymmetric – 71 % ee**

**5o**

**Racemic trace**

**Asymmetric – 75 % ee**

# 6. Cyclic Voltammetry

Current/µA

| Potential/V vs Fc/Fc+ | 5a |
| --- | --- |
| -0.407251 | -0.0955952 |
| -0.402254 | 0.0258441 |
| -0.397257 | 0.103908 |
| -0.39226 | 0.165221 |
| -0.387263 | 0.213972 |
| -0.382266 | 0.252785 |
| -0.377269 | 0.285536 |
| -0.372272 | 0.311848 |
| -0.367275 | 0.330911 |
| -0.362278 | 0.344662 |
| -0.357282 | 0.355974 |
| -0.352285 | 0.364849 |
| -0.347288 | 0.370349 |
| -0.342291 | 0.375349 |
| -0.337294 | 0.376537 |
| -0.332297 | 0.377349 |
| -0.3273 | 0.3771 |
| -0.322303 | 0.379975 |
| -0.317306 | 0.378725 |
| -0.312309 | 0.38085 |
| -0.307312 | 0.378662 |
| -0.302315 | 0.3816 |
| -0.297318 | 0.382975 |
| -0.292321 | 0.385975 |
| -0.287324 | 0.385662 |
| -0.282327 | 0.38885 |
| -0.27733 | 0.39235 |
| -0.272333 | 0.3951 |
| -0.267336 | 0.398662 |
| -0.262339 | 0.40485 |
| -0.257343 | 0.410538 |
| -0.252346 | 0.4166 |
| -0.247349 | 0.42335 |
| -0.242352 | 0.430725 |
| -0.237355 | 0.438663 |
| -0.232358 | 0.448101 |
| -0.227361 | 0.455288 |
| -0.222364 | 0.466413 |
| -0.217367 | 0.476351 |
| -0.21237 | 0.488976 |
| -0.207373 | 0.498226 |
| -0.202376 | 0.510977 |
| -0.197379 | 0.523664 |
| -0.192382 | 0.537977 |
| -0.187385 | 0.550852 |
| -0.182388 | 0.567665 |
| -0.177391 | 0.58329 |
| -0.172394 | 0.598853 |
| -0.167397 | 0.615103 |
| -0.1624 | 0.635416 |
| -0.157403 | 0.651729 |
| -0.152407 | 0.672917 |
| -0.14741 | 0.691229 |
| -0.142413 | 0.714917 |
| -0.137416 | 0.73598 |
| -0.132419 | 0.76073 |
| -0.127422 | 0.783981 |
| -0.122425 | 0.809169 |
| -0.117428 | 0.837607 |
| -0.112431 | 0.865607 |
| -0.107434 | 0.896732 |
| -0.102437 | 0.927358 |
| -0.0974401 | 0.960483 |
| -0.0924431 | 0.994046 |
| -0.0874462 | 1.03098 |
| -0.0824492 | 1.07199 |
| -0.0774523 | 1.11136 |
| -0.0724553 | 1.15517 |
| -0.0674584 | 1.19924 |
| -0.0624614 | 1.24774 |
| -0.0574645 | 1.29405 |
| -0.0524675 | 1.34436 |
| -0.0474706 | 1.39349 |
| -0.0424736 | 1.44655 |
| -0.0374767 | 1.49587 |
| -0.0324797 | 1.54787 |
| -0.0274828 | 1.59962 |
| -0.0224858 | 1.64596 |
| -0.0174889 | 1.69346 |
| -0.0124919 | 1.73784 |
| -0.00749495 | 1.77909 |
| -0.00249799 | 1.81972 |
| 0.00249896 | 1.85534 |
| 0.00749592 | 1.89034 |
| 0.0124928 | 1.91847 |
| 0.0174898 | 1.94659 |
| 0.0224868 | 1.97159 |
| 0.0274837 | 1.99472 |
| 0.0324807 | 2.01347 |
| 0.0374776 | 2.03659 |
| 0.0424746 | 2.04909 |
| 0.0474715 | 2.06847 |
| 0.0524685 | 2.08159 |
| 0.0574654 | 2.09722 |
| 0.0624624 | 2.10909 |
| 0.0674593 | 2.12409 |
| 0.0724563 | 2.1391 |
| 0.0774532 | 2.15035 |
| 0.0824502 | 2.16535 |
| 0.0874471 | 2.1791 |
| 0.0924441 | 2.19097 |
| 0.097441 | 2.2066 |
| 0.102438 | 2.21972 |
| 0.107435 | 2.2366 |
| 0.112432 | 2.25097 |
| 0.117429 | 2.27035 |
| 0.122426 | 2.28347 |
| 0.127423 | 2.30035 |
| 0.13242 | 2.31722 |
| 0.137417 | 2.33597 |
| 0.142414 | 2.35035 |
| 0.147411 | 2.36847 |
| 0.152407 | 2.38285 |
| 0.157404 | 2.3991 |
| 0.162401 | 2.41597 |
| 0.167398 | 2.4316 |
| 0.172395 | 2.44847 |
| 0.177392 | 2.46222 |
| 0.182389 | 2.47723 |
| 0.187386 | 2.4916 |
| 0.192383 | 2.51035 |
| 0.19738 | 2.5241 |
| 0.202377 | 2.53973 |
| 0.207374 | 2.55348 |
| 0.212371 | 2.5691 |
| 0.217368 | 2.5841 |
| 0.222365 | 2.6016 |
| 0.227362 | 2.61973 |
| 0.232359 | 2.6366 |
| 0.237356 | 2.65223 |
| 0.242353 | 2.66973 |
| 0.24735 | 2.68723 |
| 0.252347 | 2.70348 |
| 0.257343 | 2.7216 |
| 0.26234 | 2.7391 |
| 0.267337 | 2.75723 |
| 0.272334 | 2.77598 |
| 0.277331 | 2.79723 |
| 0.282328 | 2.81598 |
| 0.287325 | 2.83848 |
| 0.292322 | 2.85848 |
| 0.297319 | 2.88411 |
| 0.302316 | 2.90536 |
| 0.307313 | 2.92973 |
| 0.31231 | 2.95536 |
| 0.317307 | 2.98286 |
| 0.322304 | 3.01161 |
| 0.327301 | 3.03973 |
| 0.332298 | 3.07098 |
| 0.337295 | 3.10598 |
| 0.342292 | 3.14286 |
| 0.347289 | 3.18099 |
| 0.352286 | 3.22036 |
| 0.357283 | 3.26224 |
| 0.362279 | 3.30786 |
| 0.367276 | 3.35474 |
| 0.372273 | 3.40724 |
| 0.37727 | 3.45974 |
| 0.382267 | 3.51474 |
| 0.387264 | 3.57412 |
| 0.392261 | 3.63599 |
| 0.397258 | 3.70224 |
| 0.402255 | 3.76599 |
| 0.407252 | 3.841 |
| 0.412249 | 3.91475 |
| 0.417246 | 3.99412 |
| 0.422243 | 4.07475 |
| 0.42724 | 4.15725 |
| 0.432237 | 4.24975 |
| 0.437234 | 4.33913 |
| 0.442231 | 4.44101 |
| 0.447228 | 4.54538 |
| 0.452225 | 4.65413 |
| 0.457222 | 4.77539 |
| 0.462218 | 4.90101 |
| 0.467215 | 5.03539 |
| 0.472212 | 5.18664 |
| 0.477209 | 5.35102 |
| 0.482206 | 5.53477 |
| 0.487203 | 5.75353 |
| 0.4922 | 6.02165 |
| 0.497197 | 6.33666 |
| 0.502194 | 6.70104 |
| 0.507191 | 7.11042 |
| 0.512188 | 7.5648 |
| 0.517185 | 8.06044 |
| 0.522182 | 8.60794 |
| 0.527179 | 9.20733 |
| 0.532176 | 9.86609 |
| 0.537173 | 10.5742 |
| 0.54217 | 11.3417 |
| 0.547167 | 12.1617 |
| 0.552164 | 13.0418 |
| 0.557161 | 13.9693 |
| 0.562158 | 14.9418 |
| 0.567154 | 15.9568 |
| 0.572151 | 16.9846 |
| 0.577148 | 18.0597 |
| 0.582145 | 19.1472 |
| 0.587142 | 20.2409 |

| Potential/V vs Fc/Fc+ | 5d |
| --- | --- |
| -0.595356 | -0.012207 |
| -0.592914 | 0.0579834 |
| -0.590473 | 0.00305176 |
| -0.588031 | -0.112915 |
| -0.58559 | -0.0671387 |
| -0.583149 | 0.0823975 |
| -0.580707 | 0.0732422 |
| -0.578266 | -0.0183105 |
| -0.575824 | -0.0823975 |
| -0.573383 | 0.0366211 |
| -0.570941 | 0.115967 |
| -0.5685 | 0.0244141 |
| -0.566059 | -0.0701904 |
| -0.563617 | -0.0518799 |
| -0.561176 | 0.0793457 |
| -0.558735 | 0.0946045 |
| -0.556293 | 0.012207 |
| -0.553852 | -0.0640869 |
| -0.55141 | 0.0488281 |
| -0.548969 | 0.131226 |
| -0.546528 | 0.0701904 |
| -0.544086 | -0.0579834 |
| -0.541645 | -0.0366211 |
| -0.539203 | 0.10376 |
| -0.536762 | 0.109863 |
| -0.53432 | 0.0152588 |
| -0.531879 | -0.0305176 |
| -0.529438 | 0.0335693 |
| -0.526996 | 0.15564 |
| -0.524555 | 0.0946045 |
| -0.522113 | -0.0213623 |
| -0.519672 | -0.0183105 |
| -0.517231 | 0.106812 |
| -0.514789 | 0.134277 |
| -0.512348 | 0.0427246 |
| -0.509906 | -0.0152588 |
| -0.507465 | 0.0488281 |
| -0.505024 | 0.161743 |
| -0.502582 | 0.125122 |
| -0.500141 | 0.00305176 |
| -0.497699 | -0.00610352 |
| -0.495258 | 0.115967 |
| -0.492817 | 0.170898 |
| -0.490375 | 0.0671387 |
| -0.487934 | -0.012207 |
| -0.485492 | 0.0610352 |
| -0.483051 | 0.17395 |
| -0.480609 | 0.143433 |
| -0.478168 | 0.0396729 |
| -0.475727 | 0.00305176 |
| -0.473285 | 0.125122 |
| -0.470844 | 0.180054 |
| -0.468402 | 0.0976562 |
| -0.465961 | 0 |
| -0.46352 | 0.0701904 |
| -0.461078 | 0.189209 |
| -0.458637 | 0.17395 |
| -0.456195 | 0.0579834 |
| -0.453754 | 0.0305176 |
| -0.451313 | 0.140381 |
| -0.448871 | 0.192261 |
| -0.44643 | 0.12207 |
| -0.443988 | 0.0152588 |
| -0.441547 | 0.0732422 |
| -0.439106 | 0.219727 |
| -0.436664 | 0.198364 |
| -0.434223 | 0.0946045 |
| -0.431781 | 0.0518799 |
| -0.42934 | 0.15564 |
| -0.426899 | 0.213623 |
| -0.424457 | 0.158691 |
| -0.422016 | 0.0366211 |
| -0.419574 | 0.0762939 |
| -0.417133 | 0.204468 |
| -0.414692 | 0.22583 |
| -0.41225 | 0.112915 |
| -0.409809 | 0.0640869 |
| -0.407367 | 0.161743 |
| -0.404926 | 0.259399 |
| -0.402484 | 0.17395 |
| -0.400043 | 0.0701904 |
| -0.397602 | 0.10376 |
| -0.39516 | 0.219727 |
| -0.392719 | 0.265503 |
| -0.390277 | 0.164795 |
| -0.387836 | 0.0762939 |
| -0.385395 | 0.183105 |
| -0.382953 | 0.274658 |
| -0.380512 | 0.20752 |
| -0.37807 | 0.0854492 |
| -0.375629 | 0.106812 |
| -0.373188 | 0.231934 |
| -0.370746 | 0.259399 |
| -0.368305 | 0.167847 |
| -0.365863 | 0.0976562 |
| -0.363422 | 0.170898 |
| -0.360981 | 0.299072 |
| -0.358539 | 0.231934 |
| -0.356098 | 0.106812 |
| -0.353656 | 0.100708 |
| -0.351215 | 0.231934 |
| -0.348774 | 0.274658 |
| -0.346332 | 0.164795 |
| -0.343891 | 0.115967 |
| -0.341449 | 0.177002 |
| -0.339008 | 0.27771 |
| -0.336567 | 0.259399 |
| -0.334125 | 0.112915 |
| -0.331684 | 0.125122 |
| -0.329242 | 0.250244 |
| -0.326801 | 0.274658 |
| -0.324359 | 0.180054 |
| -0.321918 | 0.115967 |
| -0.319477 | 0.189209 |
| -0.317035 | 0.280762 |
| -0.314594 | 0.234985 |
| -0.312152 | 0.140381 |
| -0.309711 | 0.134277 |
| -0.30727 | 0.228882 |
| -0.304828 | 0.268555 |
| -0.302387 | 0.189209 |
| -0.299945 | 0.106812 |
| -0.297504 | 0.186157 |
| -0.295063 | 0.292969 |
| -0.292621 | 0.259399 |
| -0.29018 | 0.140381 |
| -0.287738 | 0.131226 |
| -0.285297 | 0.222778 |
| -0.282856 | 0.268555 |
| -0.280414 | 0.189209 |
| -0.277973 | 0.0946045 |
| -0.275531 | 0.161743 |
| -0.27309 | 0.274658 |
| -0.270649 | 0.250244 |
| -0.268207 | 0.146484 |
| -0.265766 | 0.125122 |
| -0.263324 | 0.231934 |
| -0.260883 | 0.274658 |
| -0.258442 | 0.213623 |
| -0.256 | 0.10376 |
| -0.253559 | 0.140381 |
| -0.251117 | 0.27771 |
| -0.248676 | 0.268555 |
| -0.246234 | 0.161743 |
| -0.243793 | 0.112915 |
| -0.241352 | 0.222778 |
| -0.23891 | 0.286865 |
| -0.236469 | 0.204468 |
| -0.234027 | 0.10376 |
| -0.231586 | 0.131226 |
| -0.229145 | 0.274658 |
| -0.226703 | 0.265503 |
| -0.224262 | 0.167847 |
| -0.22182 | 0.106812 |
| -0.219379 | 0.213623 |
| -0.216938 | 0.302124 |
| -0.214496 | 0.216675 |
| -0.212055 | 0.10376 |
| -0.209613 | 0.131226 |
| -0.207172 | 0.274658 |
| -0.204731 | 0.271606 |
| -0.202289 | 0.186157 |
| -0.199848 | 0.10376 |
| -0.197406 | 0.22583 |
| -0.194965 | 0.311279 |
| -0.192524 | 0.234985 |
| -0.190082 | 0.100708 |
| -0.187641 | 0.12207 |
| -0.185199 | 0.274658 |
| -0.182758 | 0.289917 |
| -0.180317 | 0.186157 |
| -0.177875 | 0.125122 |
| -0.175434 | 0.219727 |
| -0.172992 | 0.323486 |
| -0.170551 | 0.274658 |
| -0.168109 | 0.134277 |
| -0.165668 | 0.143433 |
| -0.163227 | 0.27771 |
| -0.160785 | 0.320435 |
| -0.158344 | 0.204468 |
| -0.155902 | 0.134277 |
| -0.153461 | 0.222778 |
| -0.15102 | 0.338745 |
| -0.148578 | 0.289917 |
| -0.146137 | 0.170898 |
| -0.143695 | 0.158691 |
| -0.141254 | 0.289917 |
| -0.138813 | 0.350952 |
| -0.136371 | 0.241089 |
| -0.13393 | 0.143433 |
| -0.131488 | 0.238037 |
| -0.129047 | 0.363159 |
| -0.126606 | 0.332642 |
| -0.124164 | 0.195313 |
| -0.121723 | 0.158691 |
| -0.119281 | 0.299072 |
| -0.11684 | 0.369263 |
| -0.114399 | 0.265503 |
| -0.111957 | 0.161743 |
| -0.109516 | 0.247192 |

| Potential/V vs Fc/Fc+ | 5e | 5f |
| --- | --- | --- |
| -0.55495 | -0.131846 | -0.139658 |
| -0.549953 | -0.0876576 | -0.0444069 |
| -0.544956 | -0.0603447 | 0.000281237 |
| -0.539959 | -0.0410319 | 0.0346568 |
| -0.534962 | -0.0234691 | 0.0602822 |
| -0.529965 | -0.0088439 | 0.0809075 |
| -0.524968 | 0.00503131 | 0.114345 |
| -0.519971 | 0.015469 | 0.136346 |
| -0.514974 | 0.0280317 | 0.158096 |
| -0.509977 | 0.0375318 | 0.163034 |
| -0.504981 | 0.0482195 | 0.190597 |
| -0.499984 | 0.0572196 | 0.216785 |
| -0.494987 | 0.0672198 | 0.224722 |
| -0.48999 | 0.0757824 | 0.23716 |
| -0.484993 | 0.0837825 | 0.255973 |
| -0.479996 | 0.0916576 | 0.258785 |
| -0.474999 | 0.0997828 | 0.286989 |
| -0.470002 | 0.107345 | 0.308786 |
| -0.465005 | 0.11547 | 0.324411 |
| -0.460008 | 0.121471 | 0.325536 |
| -0.455011 | 0.131283 | 0.323474 |
| -0.450014 | 0.136783 | 0.349412 |
| -0.445017 | 0.144533 | 0.361099 |
| -0.44002 | 0.149784 | 0.365662 |
| -0.435023 | 0.160534 | 0.375974 |
| -0.430026 | 0.166159 | 0.392412 |
| -0.425029 | 0.172471 | 0.395162 |
| -0.420032 | 0.179659 | 0.406787 |
| -0.415035 | 0.187847 | 0.395037 |
| -0.410038 | 0.193034 | 0.418725 |
| -0.405042 | 0.201597 | 0.41785 |
| -0.400045 | 0.206784 | 0.4251 |
| -0.395048 | 0.215597 | 0.434225 |
| -0.390051 | 0.22016 | 0.442163 |
| -0.385054 | 0.228222 | 0.437725 |
| -0.380057 | 0.234597 | 0.452226 |
| -0.37506 | 0.241597 | 0.448601 |
| -0.370063 | 0.24716 | 0.467601 |
| -0.365066 | 0.25441 | 0.483539 |
| -0.360069 | 0.25866 | 0.479414 |
| -0.355072 | 0.266223 | 0.488476 |
| -0.350075 | 0.27066 | 0.502101 |
| -0.345078 | 0.27741 | 0.505601 |
| -0.340081 | 0.281098 | 0.507414 |
| -0.335084 | 0.288098 | 0.506164 |
| -0.330087 | 0.292473 | 0.524914 |
| -0.32509 | 0.299036 | 0.531977 |
| -0.320093 | 0.303223 | 0.533977 |
| -0.315096 | 0.310848 | 0.546665 |
| -0.310099 | 0.314849 | 0.549102 |
| -0.305102 | 0.320411 | 0.57854 |
| -0.300106 | 0.324911 | 0.56304 |
| -0.295109 | 0.331786 | 0.587665 |
| -0.290112 | 0.336411 | 0.579228 |
| -0.285115 | 0.342286 | 0.601415 |
| -0.280118 | 0.345724 | 0.609478 |
| -0.275121 | 0.352474 | 0.620416 |
| -0.270124 | 0.357162 | 0.636478 |
| -0.265127 | 0.363724 | 0.642854 |
| -0.26013 | 0.366537 | 0.654541 |
| -0.255133 | 0.372662 | 0.671041 |
| -0.250136 | 0.377037 | 0.688667 |
| -0.245139 | 0.383662 | 0.737355 |
| -0.240142 | 0.387412 | 0.722855 |
| -0.235145 | 0.394787 | 0.743105 |
| -0.230148 | 0.397787 | 0.75973 |
| -0.225151 | 0.405287 | 0.762105 |
| -0.220154 | 0.40785 | 0.791543 |
| -0.215157 | 0.415725 | 0.805669 |
| -0.21016 | 0.419538 | 0.823981 |
| -0.205163 | 0.4261 | 0.836982 |
| -0.200167 | 0.4301 | 0.872295 |
| -0.19517 | 0.438038 | 0.876232 |
| -0.190173 | 0.440913 | 0.898232 |
| -0.185176 | 0.449913 | 0.918545 |
| -0.180179 | 0.454163 | 0.930733 |
| -0.175182 | 0.461601 | 0.965234 |
| -0.170185 | 0.465663 | 0.965984 |
| -0.165188 | 0.474788 | 1.01311 |
| -0.160191 | 0.477539 | 1.02698 |
| -0.155194 | 0.488414 | 1.05898 |
| -0.150197 | 0.493539 | 1.08117 |
| -0.1452 | 0.502351 | 1.10586 |
| -0.140203 | 0.508164 | 1.12886 |
| -0.135206 | 0.517414 | 1.17767 |
| -0.130209 | 0.523164 | 1.1878 |
| -0.125212 | 0.532602 | 1.21786 |
| -0.120215 | 0.540102 | 1.23911 |
| -0.115218 | 0.551477 | 1.27574 |
| -0.110221 | 0.55829 | 1.27218 |
| -0.105224 | 0.570227 | 1.34411 |
| -0.100227 | 0.577728 | 1.36755 |
| -0.0952305 | 0.590728 | 1.38186 |
| -0.0902336 | 0.597853 | 1.43218 |
| -0.0852366 | 0.612291 | 1.44655 |
| -0.0802397 | 0.619916 | 1.47468 |
| -0.0752427 | 0.634666 | 1.51318 |
| -0.0702458 | 0.643229 | 1.5328 |
| -0.0652488 | 0.659166 | 1.57181 |
| -0.0602519 | 0.670229 | 1.59674 |
| -0.0552549 | 0.685354 | 1.62534 |
| -0.050258 | 0.697104 | 1.62159 |
| -0.045261 | 0.713605 | 1.67909 |
| -0.0402641 | 0.726167 | 1.70909 |
| -0.0352671 | 0.743918 | 1.71846 |
| -0.0302702 | 0.756043 | 1.76409 |
| -0.0252732 | 0.772043 | 1.79471 |
| -0.0202762 | 0.786418 | 1.82597 |
| -0.0152793 | 0.805356 | 1.83409 |
| -0.0102823 | 0.818544 | 1.88659 |
| -0.00528543 | 0.836544 | 1.89909 |
| -0.000288479 | 0.848544 | 1.92159 |
| 0.00470848 | 0.867857 | 1.96909 |
| 0.00970543 | 0.880232 | 1.99472 |
| 0.0147024 | 0.898607 | 2.02472 |
| 0.0196993 | 0.91292 | 2.05659 |
| 0.0246963 | 0.93067 | 2.10659 |
| 0.0296933 | 0.942796 | 2.11972 |
| 0.0346902 | 0.959233 | 2.16097 |
| 0.0396872 | 0.972171 | 2.1841 |
| 0.0446841 | 0.988859 | 2.19785 |
| 0.0496811 | 1.0023 | 2.1991 |
| 0.054678 | 1.01867 | 2.28722 |
| 0.059675 | 1.03286 | 2.31347 |
| 0.0646719 | 1.04736 | 2.33597 |
| 0.0696688 | 1.06111 | 2.39035 |
| 0.0746658 | 1.07805 | 2.41597 |
| 0.0796627 | 1.09086 | 2.4391 |
| 0.0846597 | 1.10905 | 2.50723 |
| 0.0896567 | 1.12249 | 2.52285 |
| 0.0946536 | 1.13867 | 2.5741 |
| 0.0996506 | 1.15374 | 2.60785 |
| 0.104648 | 1.1723 | 2.63223 |
| 0.109644 | 1.18361 | 2.66285 |
| 0.114641 | 1.20136 | 2.72598 |
| 0.119638 | 1.2118 | 2.78598 |
| 0.124635 | 1.23024 | 2.82973 |
| 0.129632 | 1.24361 | 2.86473 |
| 0.134629 | 1.25943 | 2.91473 |
| 0.139626 | 1.27361 | 2.98661 |
| 0.144623 | 1.29055 | 3.03411 |
| 0.14962 | 1.30436 | 3.07973 |
| 0.154617 | 1.31986 | 3.12786 |
| 0.159614 | 1.33436 | 3.15661 |
| 0.164611 | 1.35149 | 3.22724 |
| 0.169608 | 1.36461 | 3.32224 |
| 0.174605 | 1.38143 | 3.36036 |
| 0.179602 | 1.39662 | 3.43786 |
| 0.184599 | 1.41149 | 3.52412 |
| 0.189596 | 1.42755 | 3.60412 |
| 0.194593 | 1.44124 | 3.69037 |
| 0.19959 | 1.45824 | 3.776 |
| 0.204587 | 1.4733 | 3.8535 |
| 0.209584 | 1.4873 | 3.93912 |
| 0.21458 | 1.50505 | 4.03037 |
| 0.219577 | 1.5208 | 4.1235 |
| 0.224574 | 1.53518 | 4.201 |
| 0.229571 | 1.55193 | 4.2885 |
| 0.234568 | 1.56631 | 4.37038 |
| 0.239565 | 1.58118 | 4.44351 |
| 0.244562 | 1.59784 | 4.54226 |
| 0.249559 | 1.61471 | 4.60663 |
| 0.254556 | 1.63096 | 4.68413 |
| 0.259553 | 1.64471 | 4.74726 |
| 0.26455 | 1.66346 | 4.81414 |
| 0.269547 | 1.68096 | 4.87976 |
| 0.274544 | 1.69534 | 4.92726 |
| 0.279541 | 1.71284 | 4.97726 |
| 0.284538 | 1.72971 | 5.04101 |
| 0.289535 | 1.74846 | 5.09477 |
| 0.294532 | 1.76721 | 5.15352 |
| 0.299529 | 1.78596 | 5.18164 |
| 0.304526 | 1.80471 | 5.25164 |
| 0.309523 | 1.82722 | 5.28477 |
| 0.314519 | 1.85097 | 5.36289 |
| 0.319516 | 1.87659 | 5.4279 |
| 0.324513 | 1.90409 | 5.48227 |
| 0.32951 | 1.93597 | 5.55602 |
| 0.334507 | 1.96597 | 5.62165 |
| 0.339504 | 1.99972 | 5.70352 |
| 0.344501 | 2.03722 | 5.79478 |
| 0.349498 | 2.07972 | 5.8804 |
| 0.354495 | 2.12159 | 5.9979 |
| 0.359492 | 2.16972 | 6.09603 |
| 0.364489 | 2.22035 | 6.23353 |
| 0.369486 | 2.28097 | 6.38916 |
| 0.374483 | 2.34285 | 6.54479 |
| 0.37948 | 2.40847 | 6.70979 |
| 0.384477 | 2.48035 | 6.92167 |
| 0.389474 | 2.55348 | 7.13167 |
| 0.394471 | 2.63473 | 7.36605 |
| 0.399468 | 2.7191 | 7.6073 |
| 0.404465 | 2.80786 | 7.89043 |
| 0.409462 | 2.90598 | 8.20106 |
| 0.414459 | 3.00536 | 8.55669 |
| 0.419455 | 3.10973 | 8.95232 |
| 0.424452 | 3.21661 | 9.39858 |
| 0.429449 | 3.33474 | 9.89734 |
| 0.434446 | 3.44849 | 10.4405 |
| 0.439443 | 3.57162 | 11.0399 |

| Potential/V vs Fc/Fc+ | 5g |
| --- | --- |
| -0.600238 | 0.119019 |
| -0.60268 | 0.20752 |
| -0.605121 | 0.0762939 |
| -0.607563 | -0.164795 |
| -0.610004 | -0.0946045 |
| -0.612445 | 0.128174 |
| -0.614887 | 0.10376 |
| -0.617328 | -0.0549316 |
| -0.61977 | -0.180054 |
| -0.622211 | -0.00915527 |
| -0.624653 | 0.149536 |
| -0.627094 | 0.0274658 |
| -0.629535 | -0.180054 |
| -0.631977 | -0.167847 |
| -0.634418 | 0.0549316 |
| -0.63686 | 0.0762939 |
| -0.639301 | -0.0946045 |
| -0.641742 | -0.222778 |
| -0.644184 | -0.0854492 |
| -0.646625 | 0.109863 |
| -0.649066 | 0.0335693 |
| -0.651508 | -0.17395 |
| -0.653949 | -0.204468 |
| -0.656391 | -0.012207 |
| -0.658832 | 0.0732422 |
| -0.661274 | -0.0854492 |
| -0.663715 | -0.283813 |
| -0.666156 | -0.17395 |
| -0.668598 | 0.0488281 |
| -0.671039 | 0.012207 |
| -0.673481 | -0.20752 |
| -0.675922 | -0.256348 |
| -0.678363 | -0.0610352 |
| -0.680805 | 0.0152588 |
| -0.683246 | -0.100708 |
| -0.685688 | -0.314331 |
| -0.688129 | -0.247192 |
| -0.69057 | -0.0305176 |
| -0.693012 | -0.0335693 |
| -0.695453 | -0.201416 |
| -0.697895 | -0.323486 |
| -0.700336 | -0.143433 |
| -0.702777 | 0.0183105 |
| -0.705219 | -0.100708 |
| -0.70766 | -0.317383 |
| -0.710102 | -0.308228 |
| -0.712543 | -0.0671387 |
| -0.714985 | -0.0396729 |
| -0.717426 | -0.216675 |
| -0.719867 | -0.338745 |
| -0.722309 | -0.213623 |
| -0.72475 | -0.0152588 |
| -0.727192 | -0.0793457 |
| -0.729633 | -0.286865 |
| -0.732074 | -0.341797 |
| -0.734516 | -0.115967 |
| -0.736957 | -0.0213623 |
| -0.739399 | -0.195313 |
| -0.74184 | -0.408936 |
| -0.744281 | -0.271606 |
| -0.746723 | -0.0366211 |
| -0.749164 | -0.0915527 |
| -0.751606 | -0.302124 |
| -0.754047 | -0.387573 |
| -0.756488 | -0.17395 |
| -0.75893 | -0.0671387 |
| -0.761371 | -0.201416 |
| -0.763813 | -0.430298 |
| -0.766254 | -0.378418 |
| -0.768695 | -0.115967 |
| -0.771137 | -0.10376 |
| -0.773578 | -0.302124 |
| -0.77602 | -0.436401 |
| -0.778461 | -0.228882 |
| -0.780902 | -0.0732422 |
| -0.783344 | -0.201416 |
| -0.785785 | -0.448608 |
| -0.788227 | -0.424194 |
| -0.790668 | -0.17395 |
| -0.79311 | -0.137329 |
| -0.795551 | -0.32959 |
| -0.797992 | -0.45166 |
| -0.800434 | -0.296021 |
| -0.802875 | -0.0946045 |
| -0.805317 | -0.180054 |
| -0.807758 | -0.393677 |
| -0.810199 | -0.45166 |
| -0.812641 | -0.222778 |
| -0.815082 | -0.109863 |
| -0.817524 | -0.314331 |
| -0.819965 | -0.512695 |
| -0.822406 | -0.372314 |
| -0.824848 | -0.125122 |
| -0.827289 | -0.17395 |
| -0.829731 | -0.411987 |
| -0.832172 | -0.488281 |
| -0.834613 | -0.250244 |
| -0.837055 | -0.158691 |
| -0.839496 | -0.302124 |
| -0.841938 | -0.543213 |
| -0.844379 | -0.488281 |
| -0.84682 | -0.216675 |
| -0.849262 | -0.20752 |
| -0.851703 | -0.411987 |
| -0.854145 | -0.558472 |
| -0.856586 | -0.320435 |
| -0.859027 | -0.146484 |
| -0.861469 | -0.308228 |
| -0.86391 | -0.552368 |
| -0.866352 | -0.512695 |
| -0.868793 | -0.253296 |
| -0.871235 | -0.219727 |
| -0.873676 | -0.439453 |
| -0.876117 | -0.564575 |
| -0.878559 | -0.408936 |
| -0.881 | -0.186157 |
| -0.883442 | -0.268555 |
| -0.885883 | -0.500488 |
| -0.888324 | -0.558472 |
| -0.890766 | -0.320435 |
| -0.893207 | -0.201416 |
| -0.895649 | -0.396729 |
| -0.89809 | -0.616455 |
| -0.900531 | -0.45166 |
| -0.902973 | -0.20752 |
| -0.905414 | -0.262451 |
| -0.907856 | -0.521851 |
| -0.910297 | -0.592041 |
| -0.912738 | -0.341797 |
| -0.91518 | -0.238037 |
| -0.917621 | -0.38147 |
| -0.920063 | -0.653076 |
| -0.922504 | -0.576782 |
| -0.924945 | -0.280762 |
| -0.927387 | -0.27771 |
| -0.929828 | -0.509644 |
| -0.93227 | -0.646973 |
| -0.934711 | -0.427246 |
| -0.937152 | -0.241089 |
| -0.939594 | -0.405884 |
| -0.942035 | -0.665283 |
| -0.944477 | -0.616455 |
| -0.946918 | -0.335693 |
| -0.94936 | -0.302124 |
| -0.951801 | -0.515747 |
| -0.954242 | -0.668335 |
| -0.956684 | -0.491333 |
| -0.959125 | -0.259399 |
| -0.961567 | -0.350952 |
| -0.964008 | -0.631714 |
| -0.966449 | -0.67749 |
| -0.968891 | -0.39978 |
| -0.971332 | -0.292969 |
| -0.973774 | -0.506592 |
| -0.976215 | -0.701904 |
| -0.978656 | -0.552368 |
| -0.981098 | -0.274658 |
| -0.983539 | -0.341797 |
| -0.985981 | -0.610352 |
| -0.988422 | -0.689697 |
| -0.990863 | -0.439453 |
| -0.993305 | -0.341797 |
| -0.995746 | -0.497437 |
| -0.998188 | -0.756836 |
| -1.00063 | -0.680542 |
| -1.00307 | -0.384521 |
| -1.00551 | -0.384521 |
| -1.00795 | -0.628662 |
| -1.01039 | -0.756836 |
| -1.01284 | -0.524902 |
| -1.01528 | -0.344849 |
| -1.01772 | -0.527954 |
| -1.02016 | -0.808716 |
| -1.0226 | -0.753784 |
| -1.02504 | -0.463867 |
| -1.02748 | -0.439453 |
| -1.02993 | -0.671387 |
| -1.03237 | -0.805664 |
| -1.03481 | -0.610352 |
| -1.03725 | -0.405884 |
| -1.03969 | -0.506592 |
| -1.04213 | -0.787354 |
| -1.04457 | -0.808716 |
| -1.04702 | -0.524902 |
| -1.04946 | -0.448608 |
| -1.0519 | -0.680542 |
| -1.05434 | -0.872803 |
| -1.05678 | -0.723267 |
| -1.05922 | -0.448608 |
| -1.06166 | -0.531006 |
| -1.06411 | -0.796509 |
| -1.06655 | -0.872803 |
| -1.06899 | -0.6073 |
| -1.07143 | -0.50354 |
| -1.07387 | -0.692749 |
| -1.07631 | -0.958252 |
| -1.07875 | -0.848389 |
| -1.0812 | -0.570679 |
| -1.08364 | -0.601196 |
| -1.08608 | -0.872803 |

**Figure S2.** CVs of 5**a**, 5**d**, 5**e**, 5**f** and 5**g** (5mM) recorded in MeCN/TBAP (0.15 M) at a scan rate of 100 mV s¹. Potentials referenced to Fc/Fc.

| Potential (V vs. Fc/Fc+) | Current (µA) |
| --- | --- |
| -0.407251 | -0.0955952 |
| -0.402254 | 0.0258441 |
| -0.397257 | 0.103908 |
| -0.39226 | 0.165221 |
| -0.387263 | 0.213972 |
| -0.382266 | 0.252785 |
| -0.377269 | 0.285536 |
| -0.372272 | 0.311848 |
| -0.367275 | 0.330911 |
| -0.362278 | 0.344662 |
| -0.357282 | 0.355974 |
| -0.352285 | 0.364849 |
| -0.347288 | 0.370349 |
| -0.342291 | 0.375349 |
| -0.337294 | 0.376537 |
| -0.332297 | 0.377349 |
| -0.3273 | 0.3771 |
| -0.322303 | 0.379975 |
| -0.317306 | 0.378725 |
| -0.312309 | 0.38085 |
| -0.307312 | 0.378662 |
| -0.302315 | 0.3816 |
| -0.297318 | 0.382975 |
| -0.292321 | 0.385975 |
| -0.287324 | 0.385662 |
| -0.282327 | 0.38885 |
| -0.27733 | 0.39235 |
| -0.272333 | 0.3951 |
| -0.267336 | 0.398662 |
| -0.262339 | 0.40485 |
| -0.257343 | 0.410538 |
| -0.252346 | 0.4166 |
| -0.247349 | 0.42335 |
| -0.242352 | 0.430725 |
| -0.237355 | 0.438663 |
| -0.232358 | 0.448101 |
| -0.227361 | 0.455288 |
| -0.222364 | 0.466413 |
| -0.217367 | 0.476351 |
| -0.21237 | 0.488976 |
| -0.207373 | 0.498226 |
| -0.202376 | 0.510977 |
| -0.197379 | 0.523664 |
| -0.192382 | 0.537977 |
| -0.187385 | 0.550852 |
| -0.182388 | 0.567665 |
| -0.177391 | 0.58329 |
| -0.172394 | 0.598853 |
| -0.167397 | 0.615103 |
| -0.1624 | 0.635416 |
| -0.157403 | 0.651729 |
| -0.152407 | 0.672917 |
| -0.14741 | 0.691229 |
| -0.142413 | 0.714917 |
| -0.137416 | 0.73598 |
| -0.132419 | 0.76073 |
| -0.127422 | 0.783981 |
| -0.122425 | 0.809169 |
| -0.117428 | 0.837607 |
| -0.112431 | 0.865607 |
| -0.107434 | 0.896732 |
| -0.102437 | 0.927358 |
| -0.0974401 | 0.960483 |
| -0.0924431 | 0.994046 |
| -0.0874462 | 1.03098 |
| -0.0824492 | 1.07199 |
| -0.0774523 | 1.11136 |
| -0.0724553 | 1.15517 |
| -0.0674584 | 1.19924 |
| -0.0624614 | 1.24774 |
| -0.0574645 | 1.29405 |
| -0.0524675 | 1.34436 |
| -0.0474706 | 1.39349 |
| -0.0424736 | 1.44655 |
| -0.0374767 | 1.49587 |
| -0.0324797 | 1.54787 |
| -0.0274828 | 1.59962 |
| -0.0224858 | 1.64596 |
| -0.0174889 | 1.69346 |
| -0.0124919 | 1.73784 |
| -0.00749495 | 1.77909 |
| -0.00249799 | 1.81972 |
| 0.00249896 | 1.85534 |
| 0.00749592 | 1.89034 |
| 0.0124928 | 1.91847 |
| 0.0174898 | 1.94659 |
| 0.0224868 | 1.97159 |
| 0.0274837 | 1.99472 |
| 0.0324807 | 2.01347 |
| 0.0374776 | 2.03659 |
| 0.0424746 | 2.04909 |
| 0.0474715 | 2.06847 |
| 0.0524685 | 2.08159 |
| 0.0574654 | 2.09722 |
| 0.0624624 | 2.10909 |
| 0.0674593 | 2.12409 |
| 0.0724563 | 2.1391 |
| 0.0774532 | 2.15035 |
| 0.0824502 | 2.16535 |
| 0.0874471 | 2.1791 |
| 0.0924441 | 2.19097 |
| 0.097441 | 2.2066 |
| 0.102438 | 2.21972 |
| 0.107435 | 2.2366 |
| 0.112432 | 2.25097 |
| 0.117429 | 2.27035 |
| 0.122426 | 2.28347 |
| 0.127423 | 2.30035 |
| 0.13242 | 2.31722 |
| 0.137417 | 2.33597 |
| 0.142414 | 2.35035 |
| 0.147411 | 2.36847 |
| 0.152407 | 2.38285 |
| 0.157404 | 2.3991 |
| 0.162401 | 2.41597 |
| 0.167398 | 2.4316 |
| 0.172395 | 2.44847 |
| 0.177392 | 2.46222 |
| 0.182389 | 2.47723 |
| 0.187386 | 2.4916 |
| 0.192383 | 2.51035 |
| 0.19738 | 2.5241 |
| 0.202377 | 2.53973 |
| 0.207374 | 2.55348 |
| 0.212371 | 2.5691 |
| 0.217368 | 2.5841 |
| 0.222365 | 2.6016 |
| 0.227362 | 2.61973 |
| 0.232359 | 2.6366 |
| 0.237356 | 2.65223 |
| 0.242353 | 2.66973 |
| 0.24735 | 2.68723 |
| 0.252347 | 2.70348 |
| 0.257343 | 2.7216 |
| 0.26234 | 2.7391 |
| 0.267337 | 2.75723 |
| 0.272334 | 2.77598 |
| 0.277331 | 2.79723 |
| 0.282328 | 2.81598 |
| 0.287325 | 2.83848 |
| 0.292322 | 2.85848 |
| 0.297319 | 2.88411 |
| 0.302316 | 2.90536 |
| 0.307313 | 2.92973 |
| 0.31231 | 2.95536 |
| 0.317307 | 2.98286 |
| 0.322304 | 3.01161 |
| 0.327301 | 3.03973 |
| 0.332298 | 3.07098 |
| 0.337295 | 3.10598 |
| 0.342292 | 3.14286 |
| 0.347289 | 3.18099 |
| 0.352286 | 3.22036 |
| 0.357283 | 3.26224 |
| 0.362279 | 3.30786 |
| 0.367276 | 3.35474 |
| 0.372273 | 3.40724 |
| 0.37727 | 3.45974 |
| 0.382267 | 3.51474 |
| 0.387264 | 3.57412 |
| 0.392261 | 3.63599 |
| 0.397258 | 3.70224 |
| 0.402255 | 3.76599 |
| 0.407252 | 3.841 |
| 0.412249 | 3.91475 |
| 0.417246 | 3.99412 |
| 0.422243 | 4.07475 |
| 0.42724 | 4.15725 |
| 0.432237 | 4.24975 |
| 0.437234 | 4.33913 |
| 0.442231 | 4.44101 |
| 0.447228 | 4.54538 |
| 0.452225 | 4.65413 |
| 0.457222 | 4.77539 |
| 0.462218 | 4.90101 |
| 0.467215 | 5.03539 |
| 0.472212 | 5.18664 |
| 0.477209 | 5.35102 |
| 0.482206 | 5.53477 |
| 0.487203 | 5.75353 |
| 0.4922 | 6.02165 |
| 0.497197 | 6.33666 |
| 0.502194 | 6.70104 |
| 0.507191 | 7.11042 |
| 0.512188 | 7.5648 |
| 0.517185 | 8.06044 |
| 0.522182 | 8.60794 |
| 0.527179 | 9.20733 |
| 0.532176 | 9.86609 |
| 0.537173 | 10.5742 |
| 0.54217 | 11.3417 |
| 0.547167 | 12.1617 |
| 0.552164 | 13.0418 |
| 0.557161 | 13.9693 |
| 0.562158 | 14.9418 |
| 0.567154 | 15.9568 |
| 0.572151 | 16.9846 |
| 0.577148 | 18.0597 |
| 0.582145 | 19.1472 |
| 0.587142 | 20.2409 |

**Figure S3.** CV of **5a** (5mM) recorded in MeCN/TBAP (0.15 M) at a scan rate of 100 mV s⁻¹. Potentials referenced to Fc/Fc⁺.

| Potential (V vs. Fc/Fc+) | Current (µA) |
| --- | --- |
| -0.595356 | -0.012207 |
| -0.592914 | 0.0579834 |
| -0.590473 | 0.00305176 |
| -0.588031 | -0.112915 |
| -0.58559 | -0.0671387 |
| -0.583149 | 0.0823975 |
| -0.580707 | 0.0732422 |
| -0.578266 | -0.0183105 |
| -0.575824 | -0.0823975 |
| -0.573383 | 0.0366211 |
| -0.570941 | 0.115967 |
| -0.5685 | 0.0244141 |
| -0.566059 | -0.0701904 |
| -0.563617 | -0.0518799 |
| -0.561176 | 0.0793457 |
| -0.558735 | 0.0946045 |
| -0.556293 | 0.012207 |
| -0.553852 | -0.0640869 |
| -0.55141 | 0.0488281 |
| -0.548969 | 0.131226 |
| -0.546528 | 0.0701904 |
| -0.544086 | -0.0579834 |
| -0.541645 | -0.0366211 |
| -0.539203 | 0.10376 |
| -0.536762 | 0.109863 |
| -0.53432 | 0.0152588 |
| -0.531879 | -0.0305176 |
| -0.529438 | 0.0335693 |
| -0.526996 | 0.15564 |
| -0.524555 | 0.0946045 |
| -0.522113 | -0.0213623 |
| -0.519672 | -0.0183105 |
| -0.517231 | 0.106812 |
| -0.514789 | 0.134277 |
| -0.512348 | 0.0427246 |
| -0.509906 | -0.0152588 |
| -0.507465 | 0.0488281 |
| -0.505024 | 0.161743 |
| -0.502582 | 0.125122 |
| -0.500141 | 0.00305176 |
| -0.497699 | -0.00610352 |
| -0.495258 | 0.115967 |
| -0.492817 | 0.170898 |
| -0.490375 | 0.0671387 |
| -0.487934 | -0.012207 |
| -0.485492 | 0.0610352 |
| -0.483051 | 0.17395 |
| -0.480609 | 0.143433 |
| -0.478168 | 0.0396729 |
| -0.475727 | 0.00305176 |
| -0.473285 | 0.125122 |
| -0.470844 | 0.180054 |
| -0.468402 | 0.0976562 |
| -0.465961 | 0 |
| -0.46352 | 0.0701904 |
| -0.461078 | 0.189209 |
| -0.458637 | 0.17395 |
| -0.456195 | 0.0579834 |
| -0.453754 | 0.0305176 |
| -0.451313 | 0.140381 |
| -0.448871 | 0.192261 |
| -0.44643 | 0.12207 |
| -0.443988 | 0.0152588 |
| -0.441547 | 0.0732422 |
| -0.439106 | 0.219727 |
| -0.436664 | 0.198364 |
| -0.434223 | 0.0946045 |
| -0.431781 | 0.0518799 |
| -0.42934 | 0.15564 |
| -0.426899 | 0.213623 |
| -0.424457 | 0.158691 |
| -0.422016 | 0.0366211 |
| -0.419574 | 0.0762939 |
| -0.417133 | 0.204468 |
| -0.414692 | 0.22583 |
| -0.41225 | 0.112915 |
| -0.409809 | 0.0640869 |
| -0.407367 | 0.161743 |
| -0.404926 | 0.259399 |
| -0.402484 | 0.17395 |
| -0.400043 | 0.0701904 |
| -0.397602 | 0.10376 |
| -0.39516 | 0.219727 |
| -0.392719 | 0.265503 |
| -0.390277 | 0.164795 |
| -0.387836 | 0.0762939 |
| -0.385395 | 0.183105 |
| -0.382953 | 0.274658 |
| -0.380512 | 0.20752 |
| -0.37807 | 0.0854492 |
| -0.375629 | 0.106812 |
| -0.373188 | 0.231934 |
| -0.370746 | 0.259399 |
| -0.368305 | 0.167847 |
| -0.365863 | 0.0976562 |
| -0.363422 | 0.170898 |
| -0.360981 | 0.299072 |
| -0.358539 | 0.231934 |
| -0.356098 | 0.106812 |
| -0.353656 | 0.100708 |
| -0.351215 | 0.231934 |
| -0.348774 | 0.274658 |
| -0.346332 | 0.164795 |
| -0.343891 | 0.115967 |
| -0.341449 | 0.177002 |
| -0.339008 | 0.27771 |
| -0.336567 | 0.259399 |
| -0.334125 | 0.112915 |
| -0.331684 | 0.125122 |
| -0.329242 | 0.250244 |
| -0.326801 | 0.274658 |
| -0.324359 | 0.180054 |
| -0.321918 | 0.115967 |
| -0.319477 | 0.189209 |
| -0.317035 | 0.280762 |
| -0.314594 | 0.234985 |
| -0.312152 | 0.140381 |
| -0.309711 | 0.134277 |
| -0.30727 | 0.228882 |
| -0.304828 | 0.268555 |
| -0.302387 | 0.189209 |
| -0.299945 | 0.106812 |
| -0.297504 | 0.186157 |
| -0.295063 | 0.292969 |
| -0.292621 | 0.259399 |
| -0.29018 | 0.140381 |
| -0.287738 | 0.131226 |
| -0.285297 | 0.222778 |
| -0.282856 | 0.268555 |
| -0.280414 | 0.189209 |
| -0.277973 | 0.0946045 |
| -0.275531 | 0.161743 |
| -0.27309 | 0.274658 |
| -0.270649 | 0.250244 |
| -0.268207 | 0.146484 |
| -0.265766 | 0.125122 |
| -0.263324 | 0.231934 |
| -0.260883 | 0.274658 |
| -0.258442 | 0.213623 |
| -0.256 | 0.10376 |
| -0.253559 | 0.140381 |
| -0.251117 | 0.27771 |
| -0.248676 | 0.268555 |
| -0.246234 | 0.161743 |
| -0.243793 | 0.112915 |
| -0.241352 | 0.222778 |
| -0.23891 | 0.286865 |
| -0.236469 | 0.204468 |
| -0.234027 | 0.10376 |
| -0.231586 | 0.131226 |
| -0.229145 | 0.274658 |
| -0.226703 | 0.265503 |
| -0.224262 | 0.167847 |
| -0.22182 | 0.106812 |
| -0.219379 | 0.213623 |
| -0.216938 | 0.302124 |
| -0.214496 | 0.216675 |
| -0.212055 | 0.10376 |
| -0.209613 | 0.131226 |
| -0.207172 | 0.274658 |
| -0.204731 | 0.271606 |
| -0.202289 | 0.186157 |
| -0.199848 | 0.10376 |
| -0.197406 | 0.22583 |
| -0.194965 | 0.311279 |
| -0.192524 | 0.234985 |
| -0.190082 | 0.100708 |
| -0.187641 | 0.12207 |
| -0.185199 | 0.274658 |
| -0.182758 | 0.289917 |
| -0.180317 | 0.186157 |
| -0.177875 | 0.125122 |
| -0.175434 | 0.219727 |
| -0.172992 | 0.323486 |
| -0.170551 | 0.274658 |
| -0.168109 | 0.134277 |
| -0.165668 | 0.143433 |
| -0.163227 | 0.27771 |
| -0.160785 | 0.320435 |
| -0.158344 | 0.204468 |
| -0.155902 | 0.134277 |
| -0.153461 | 0.222778 |
| -0.15102 | 0.338745 |
| -0.148578 | 0.289917 |
| -0.146137 | 0.170898 |
| -0.143695 | 0.158691 |
| -0.141254 | 0.289917 |
| -0.138813 | 0.350952 |
| -0.136371 | 0.241089 |
| -0.13393 | 0.143433 |
| -0.131488 | 0.238037 |
| -0.129047 | 0.363159 |
| -0.126606 | 0.332642 |
| -0.124164 | 0.195313 |
| -0.121723 | 0.158691 |
| -0.119281 | 0.299072 |
| -0.11684 | 0.369263 |
| -0.114399 | 0.265503 |
| -0.111957 | 0.161743 |
| -0.109516 | 0.247192 |

**Figure S4.** CV of **5d** (5mM) recorded in MeCN/TBAP (0.15 M) at a scan rate of 100 mV s¹. Potentials referenced to Fc/Fc.

| Potential (V vs. Fc/Fc+) | Current (µA) |
| --- | --- |
| -0.55495 | -0.131846 |
| -0.549953 | -0.0876576 |
| -0.544956 | -0.0603447 |
| -0.539959 | -0.0410319 |
| -0.534962 | -0.0234691 |
| -0.529965 | -0.0088439 |
| -0.524968 | 0.00503131 |
| -0.519971 | 0.015469 |
| -0.514974 | 0.0280317 |
| -0.509977 | 0.0375318 |
| -0.504981 | 0.0482195 |
| -0.499984 | 0.0572196 |
| -0.494987 | 0.0672198 |
| -0.48999 | 0.0757824 |
| -0.484993 | 0.0837825 |
| -0.479996 | 0.0916576 |
| -0.474999 | 0.0997828 |
| -0.470002 | 0.107345 |
| -0.465005 | 0.11547 |
| -0.460008 | 0.121471 |
| -0.455011 | 0.131283 |
| -0.450014 | 0.136783 |
| -0.445017 | 0.144533 |
| -0.44002 | 0.149784 |
| -0.435023 | 0.160534 |
| -0.430026 | 0.166159 |
| -0.425029 | 0.172471 |
| -0.420032 | 0.179659 |
| -0.415035 | 0.187847 |
| -0.410038 | 0.193034 |
| -0.405042 | 0.201597 |
| -0.400045 | 0.206784 |
| -0.395048 | 0.215597 |
| -0.390051 | 0.22016 |
| -0.385054 | 0.228222 |
| -0.380057 | 0.234597 |
| -0.37506 | 0.241597 |
| -0.370063 | 0.24716 |
| -0.365066 | 0.25441 |
| -0.360069 | 0.25866 |
| -0.355072 | 0.266223 |
| -0.350075 | 0.27066 |
| -0.345078 | 0.27741 |
| -0.340081 | 0.281098 |
| -0.335084 | 0.288098 |
| -0.330087 | 0.292473 |
| -0.32509 | 0.299036 |
| -0.320093 | 0.303223 |
| -0.315096 | 0.310848 |
| -0.310099 | 0.314849 |
| -0.305102 | 0.320411 |
| -0.300106 | 0.324911 |
| -0.295109 | 0.331786 |
| -0.290112 | 0.336411 |
| -0.285115 | 0.342286 |
| -0.280118 | 0.345724 |
| -0.275121 | 0.352474 |
| -0.270124 | 0.357162 |
| -0.265127 | 0.363724 |
| -0.26013 | 0.366537 |
| -0.255133 | 0.372662 |
| -0.250136 | 0.377037 |
| -0.245139 | 0.383662 |
| -0.240142 | 0.387412 |
| -0.235145 | 0.394787 |
| -0.230148 | 0.397787 |
| -0.225151 | 0.405287 |
| -0.220154 | 0.40785 |
| -0.215157 | 0.415725 |
| -0.21016 | 0.419538 |
| -0.205163 | 0.4261 |
| -0.200167 | 0.4301 |
| -0.19517 | 0.438038 |
| -0.190173 | 0.440913 |
| -0.185176 | 0.449913 |
| -0.180179 | 0.454163 |
| -0.175182 | 0.461601 |
| -0.170185 | 0.465663 |
| -0.165188 | 0.474788 |
| -0.160191 | 0.477539 |
| -0.155194 | 0.488414 |
| -0.150197 | 0.493539 |
| -0.1452 | 0.502351 |
| -0.140203 | 0.508164 |
| -0.135206 | 0.517414 |
| -0.130209 | 0.523164 |
| -0.125212 | 0.532602 |
| -0.120215 | 0.540102 |
| -0.115218 | 0.551477 |
| -0.110221 | 0.55829 |
| -0.105224 | 0.570227 |
| -0.100227 | 0.577728 |
| -0.0952305 | 0.590728 |
| -0.0902336 | 0.597853 |
| -0.0852366 | 0.612291 |
| -0.0802397 | 0.619916 |
| -0.0752427 | 0.634666 |
| -0.0702458 | 0.643229 |
| -0.0652488 | 0.659166 |
| -0.0602519 | 0.670229 |
| -0.0552549 | 0.685354 |
| -0.050258 | 0.697104 |
| -0.045261 | 0.713605 |
| -0.0402641 | 0.726167 |
| -0.0352671 | 0.743918 |
| -0.0302702 | 0.756043 |
| -0.0252732 | 0.772043 |
| -0.0202762 | 0.786418 |
| -0.0152793 | 0.805356 |
| -0.0102823 | 0.818544 |
| -0.00528543 | 0.836544 |
| -0.000288479 | 0.848544 |
| 0.00470848 | 0.867857 |
| 0.00970543 | 0.880232 |
| 0.0147024 | 0.898607 |
| 0.0196993 | 0.91292 |
| 0.0246963 | 0.93067 |
| 0.0296933 | 0.942796 |
| 0.0346902 | 0.959233 |
| 0.0396872 | 0.972171 |
| 0.0446841 | 0.988859 |
| 0.0496811 | 1.0023 |
| 0.054678 | 1.01867 |
| 0.059675 | 1.03286 |
| 0.0646719 | 1.04736 |
| 0.0696688 | 1.06111 |
| 0.0746658 | 1.07805 |
| 0.0796627 | 1.09086 |
| 0.0846597 | 1.10905 |
| 0.0896567 | 1.12249 |
| 0.0946536 | 1.13867 |
| 0.0996506 | 1.15374 |
| 0.104648 | 1.1723 |
| 0.109644 | 1.18361 |
| 0.114641 | 1.20136 |
| 0.119638 | 1.2118 |
| 0.124635 | 1.23024 |
| 0.129632 | 1.24361 |
| 0.134629 | 1.25943 |
| 0.139626 | 1.27361 |
| 0.144623 | 1.29055 |
| 0.14962 | 1.30436 |
| 0.154617 | 1.31986 |
| 0.159614 | 1.33436 |
| 0.164611 | 1.35149 |
| 0.169608 | 1.36461 |
| 0.174605 | 1.38143 |
| 0.179602 | 1.39662 |
| 0.184599 | 1.41149 |
| 0.189596 | 1.42755 |
| 0.194593 | 1.44124 |
| 0.19959 | 1.45824 |
| 0.204587 | 1.4733 |
| 0.209584 | 1.4873 |
| 0.21458 | 1.50505 |
| 0.219577 | 1.5208 |
| 0.224574 | 1.53518 |
| 0.229571 | 1.55193 |
| 0.234568 | 1.56631 |
| 0.239565 | 1.58118 |
| 0.244562 | 1.59784 |
| 0.249559 | 1.61471 |
| 0.254556 | 1.63096 |
| 0.259553 | 1.64471 |
| 0.26455 | 1.66346 |
| 0.269547 | 1.68096 |
| 0.274544 | 1.69534 |
| 0.279541 | 1.71284 |
| 0.284538 | 1.72971 |
| 0.289535 | 1.74846 |
| 0.294532 | 1.76721 |
| 0.299529 | 1.78596 |
| 0.304526 | 1.80471 |
| 0.309523 | 1.82722 |
| 0.314519 | 1.85097 |
| 0.319516 | 1.87659 |
| 0.324513 | 1.90409 |
| 0.32951 | 1.93597 |
| 0.334507 | 1.96597 |
| 0.339504 | 1.99972 |
| 0.344501 | 2.03722 |
| 0.349498 | 2.07972 |
| 0.354495 | 2.12159 |
| 0.359492 | 2.16972 |
| 0.364489 | 2.22035 |
| 0.369486 | 2.28097 |
| 0.374483 | 2.34285 |
| 0.37948 | 2.40847 |
| 0.384477 | 2.48035 |
| 0.389474 | 2.55348 |
| 0.394471 | 2.63473 |
| 0.399468 | 2.7191 |
| 0.404465 | 2.80786 |
| 0.409462 | 2.90598 |
| 0.414459 | 3.00536 |
| 0.419455 | 3.10973 |
| 0.424452 | 3.21661 |
| 0.429449 | 3.33474 |
| 0.434446 | 3.44849 |
| 0.439443 | 3.57162 |

**Figure S5.** CV of **5e** (5mM) recorded in MeCN/TBAP (0.15 M) at a scan rate of 100 mV s⁻¹. Potentials referenced to Fc/Fc.

| Potential (V vs. Fc/Fc+) | Current (µA) |
| --- | --- |
| -0.55495 | -0.139658 |
| -0.549953 | -0.0444069 |
| -0.544956 | 0.000281237 |
| -0.539959 | 0.0346568 |
| -0.534962 | 0.0602822 |
| -0.529965 | 0.0809075 |
| -0.524968 | 0.114345 |
| -0.519971 | 0.136346 |
| -0.514974 | 0.158096 |
| -0.509977 | 0.163034 |
| -0.504981 | 0.190597 |
| -0.499984 | 0.216785 |
| -0.494987 | 0.224722 |
| -0.48999 | 0.23716 |
| -0.484993 | 0.255973 |
| -0.479996 | 0.258785 |
| -0.474999 | 0.286989 |
| -0.470002 | 0.308786 |
| -0.465005 | 0.324411 |
| -0.460008 | 0.325536 |
| -0.455011 | 0.323474 |
| -0.450014 | 0.349412 |
| -0.445017 | 0.361099 |
| -0.44002 | 0.365662 |
| -0.435023 | 0.375974 |
| -0.430026 | 0.392412 |
| -0.425029 | 0.395162 |
| -0.420032 | 0.406787 |
| -0.415035 | 0.395037 |
| -0.410038 | 0.418725 |
| -0.405042 | 0.41785 |
| -0.400045 | 0.4251 |
| -0.395048 | 0.434225 |
| -0.390051 | 0.442163 |
| -0.385054 | 0.437725 |
| -0.380057 | 0.452226 |
| -0.37506 | 0.448601 |
| -0.370063 | 0.467601 |
| -0.365066 | 0.483539 |
| -0.360069 | 0.479414 |
| -0.355072 | 0.488476 |
| -0.350075 | 0.502101 |
| -0.345078 | 0.505601 |
| -0.340081 | 0.507414 |
| -0.335084 | 0.506164 |
| -0.330087 | 0.524914 |
| -0.32509 | 0.531977 |
| -0.320093 | 0.533977 |
| -0.315096 | 0.546665 |
| -0.310099 | 0.549102 |
| -0.305102 | 0.57854 |
| -0.300106 | 0.56304 |
| -0.295109 | 0.587665 |
| -0.290112 | 0.579228 |
| -0.285115 | 0.601415 |
| -0.280118 | 0.609478 |
| -0.275121 | 0.620416 |
| -0.270124 | 0.636478 |
| -0.265127 | 0.642854 |
| -0.26013 | 0.654541 |
| -0.255133 | 0.671041 |
| -0.250136 | 0.688667 |
| -0.245139 | 0.737355 |
| -0.240142 | 0.722855 |
| -0.235145 | 0.743105 |
| -0.230148 | 0.75973 |
| -0.225151 | 0.762105 |
| -0.220154 | 0.791543 |
| -0.215157 | 0.805669 |
| -0.21016 | 0.823981 |
| -0.205163 | 0.836982 |
| -0.200167 | 0.872295 |
| -0.19517 | 0.876232 |
| -0.190173 | 0.898232 |
| -0.185176 | 0.918545 |
| -0.180179 | 0.930733 |
| -0.175182 | 0.965234 |
| -0.170185 | 0.965984 |
| -0.165188 | 1.01311 |
| -0.160191 | 1.02698 |
| -0.155194 | 1.05898 |
| -0.150197 | 1.08117 |
| -0.1452 | 1.10586 |
| -0.140203 | 1.12886 |
| -0.135206 | 1.17767 |
| -0.130209 | 1.1878 |
| -0.125212 | 1.21786 |
| -0.120215 | 1.23911 |
| -0.115218 | 1.27574 |
| -0.110221 | 1.27218 |
| -0.105224 | 1.34411 |
| -0.100227 | 1.36755 |
| -0.0952305 | 1.38186 |
| -0.0902336 | 1.43218 |
| -0.0852366 | 1.44655 |
| -0.0802397 | 1.47468 |
| -0.0752427 | 1.51318 |
| -0.0702458 | 1.5328 |
| -0.0652488 | 1.57181 |
| -0.0602519 | 1.59674 |
| -0.0552549 | 1.62534 |
| -0.050258 | 1.62159 |
| -0.045261 | 1.67909 |
| -0.0402641 | 1.70909 |
| -0.0352671 | 1.71846 |
| -0.0302702 | 1.76409 |
| -0.0252732 | 1.79471 |
| -0.0202762 | 1.82597 |
| -0.0152793 | 1.83409 |
| -0.0102823 | 1.88659 |
| -0.00528543 | 1.89909 |
| -0.000288479 | 1.92159 |
| 0.00470848 | 1.96909 |
| 0.00970543 | 1.99472 |
| 0.0147024 | 2.02472 |
| 0.0196993 | 2.05659 |
| 0.0246963 | 2.10659 |
| 0.0296933 | 2.11972 |
| 0.0346902 | 2.16097 |
| 0.0396872 | 2.1841 |
| 0.0446841 | 2.19785 |
| 0.0496811 | 2.1991 |
| 0.054678 | 2.28722 |
| 0.059675 | 2.31347 |
| 0.0646719 | 2.33597 |
| 0.0696688 | 2.39035 |
| 0.0746658 | 2.41597 |
| 0.0796627 | 2.4391 |
| 0.0846597 | 2.50723 |
| 0.0896567 | 2.52285 |
| 0.0946536 | 2.5741 |
| 0.0996506 | 2.60785 |
| 0.104648 | 2.63223 |
| 0.109644 | 2.66285 |
| 0.114641 | 2.72598 |
| 0.119638 | 2.78598 |
| 0.124635 | 2.82973 |
| 0.129632 | 2.86473 |
| 0.134629 | 2.91473 |
| 0.139626 | 2.98661 |
| 0.144623 | 3.03411 |
| 0.14962 | 3.07973 |
| 0.154617 | 3.12786 |
| 0.159614 | 3.15661 |
| 0.164611 | 3.22724 |
| 0.169608 | 3.32224 |
| 0.174605 | 3.36036 |
| 0.179602 | 3.43786 |
| 0.184599 | 3.52412 |
| 0.189596 | 3.60412 |
| 0.194593 | 3.69037 |
| 0.19959 | 3.776 |
| 0.204587 | 3.8535 |
| 0.209584 | 3.93912 |
| 0.21458 | 4.03037 |
| 0.219577 | 4.1235 |
| 0.224574 | 4.201 |
| 0.229571 | 4.2885 |
| 0.234568 | 4.37038 |
| 0.239565 | 4.44351 |
| 0.244562 | 4.54226 |
| 0.249559 | 4.60663 |
| 0.254556 | 4.68413 |
| 0.259553 | 4.74726 |
| 0.26455 | 4.81414 |
| 0.269547 | 4.87976 |
| 0.274544 | 4.92726 |
| 0.279541 | 4.97726 |
| 0.284538 | 5.04101 |
| 0.289535 | 5.09477 |
| 0.294532 | 5.15352 |
| 0.299529 | 5.18164 |
| 0.304526 | 5.25164 |
| 0.309523 | 5.28477 |
| 0.314519 | 5.36289 |
| 0.319516 | 5.4279 |
| 0.324513 | 5.48227 |
| 0.32951 | 5.55602 |
| 0.334507 | 5.62165 |
| 0.339504 | 5.70352 |
| 0.344501 | 5.79478 |
| 0.349498 | 5.8804 |
| 0.354495 | 5.9979 |
| 0.359492 | 6.09603 |
| 0.364489 | 6.23353 |
| 0.369486 | 6.38916 |
| 0.374483 | 6.54479 |
| 0.37948 | 6.70979 |
| 0.384477 | 6.92167 |
| 0.389474 | 7.13167 |
| 0.394471 | 7.36605 |
| 0.399468 | 7.6073 |
| 0.404465 | 7.89043 |
| 0.409462 | 8.20106 |
| 0.414459 | 8.55669 |
| 0.419455 | 8.95232 |
| 0.424452 | 9.39858 |
| 0.429449 | 9.89734 |
| 0.434446 | 10.4405 |
| 0.439443 | 11.0399 |

**Figure S6.** CV of **5f** (5mM) recorded in MeCN/TBAP (0.15 M) at a scan rate of 100 mV s⁻¹. Potentials referenced to Fc/Fc.

| Potential (V vs. Fc/Fc+) | Current (µA) |
| --- | --- |
| -0.600238 | 0.119019 |
| -0.60268 | 0.20752 |
| -0.605121 | 0.0762939 |
| -0.607563 | -0.164795 |
| -0.610004 | -0.0946045 |
| -0.612445 | 0.128174 |
| -0.614887 | 0.10376 |
| -0.617328 | -0.0549316 |
| -0.61977 | -0.180054 |
| -0.622211 | -0.00915527 |
| -0.624653 | 0.149536 |
| -0.627094 | 0.0274658 |
| -0.629535 | -0.180054 |
| -0.631977 | -0.167847 |
| -0.634418 | 0.0549316 |
| -0.63686 | 0.0762939 |
| -0.639301 | -0.0946045 |
| -0.641742 | -0.222778 |
| -0.644184 | -0.0854492 |
| -0.646625 | 0.109863 |
| -0.649066 | 0.0335693 |
| -0.651508 | -0.17395 |
| -0.653949 | -0.204468 |
| -0.656391 | -0.012207 |
| -0.658832 | 0.0732422 |
| -0.661274 | -0.0854492 |
| -0.663715 | -0.283813 |
| -0.666156 | -0.17395 |
| -0.668598 | 0.0488281 |
| -0.671039 | 0.012207 |
| -0.673481 | -0.20752 |
| -0.675922 | -0.256348 |
| -0.678363 | -0.0610352 |
| -0.680805 | 0.0152588 |
| -0.683246 | -0.100708 |
| -0.685688 | -0.314331 |
| -0.688129 | -0.247192 |
| -0.69057 | -0.0305176 |
| -0.693012 | -0.0335693 |
| -0.695453 | -0.201416 |
| -0.697895 | -0.323486 |
| -0.700336 | -0.143433 |
| -0.702777 | 0.0183105 |
| -0.705219 | -0.100708 |
| -0.70766 | -0.317383 |
| -0.710102 | -0.308228 |
| -0.712543 | -0.0671387 |
| -0.714985 | -0.0396729 |
| -0.717426 | -0.216675 |
| -0.719867 | -0.338745 |
| -0.722309 | -0.213623 |
| -0.72475 | -0.0152588 |
| -0.727192 | -0.0793457 |
| -0.729633 | -0.286865 |
| -0.732074 | -0.341797 |
| -0.734516 | -0.115967 |
| -0.736957 | -0.0213623 |
| -0.739399 | -0.195313 |
| -0.74184 | -0.408936 |
| -0.744281 | -0.271606 |
| -0.746723 | -0.0366211 |
| -0.749164 | -0.0915527 |
| -0.751606 | -0.302124 |
| -0.754047 | -0.387573 |
| -0.756488 | -0.17395 |
| -0.75893 | -0.0671387 |
| -0.761371 | -0.201416 |
| -0.763813 | -0.430298 |
| -0.766254 | -0.378418 |
| -0.768695 | -0.115967 |
| -0.771137 | -0.10376 |
| -0.773578 | -0.302124 |
| -0.77602 | -0.436401 |
| -0.778461 | -0.228882 |
| -0.780902 | -0.0732422 |
| -0.783344 | -0.201416 |
| -0.785785 | -0.448608 |
| -0.788227 | -0.424194 |
| -0.790668 | -0.17395 |
| -0.79311 | -0.137329 |
| -0.795551 | -0.32959 |
| -0.797992 | -0.45166 |
| -0.800434 | -0.296021 |
| -0.802875 | -0.0946045 |
| -0.805317 | -0.180054 |
| -0.807758 | -0.393677 |
| -0.810199 | -0.45166 |
| -0.812641 | -0.222778 |
| -0.815082 | -0.109863 |
| -0.817524 | -0.314331 |
| -0.819965 | -0.512695 |
| -0.822406 | -0.372314 |
| -0.824848 | -0.125122 |
| -0.827289 | -0.17395 |
| -0.829731 | -0.411987 |
| -0.832172 | -0.488281 |
| -0.834613 | -0.250244 |
| -0.837055 | -0.158691 |
| -0.839496 | -0.302124 |
| -0.841938 | -0.543213 |
| -0.844379 | -0.488281 |
| -0.84682 | -0.216675 |
| -0.849262 | -0.20752 |
| -0.851703 | -0.411987 |
| -0.854145 | -0.558472 |
| -0.856586 | -0.320435 |
| -0.859027 | -0.146484 |
| -0.861469 | -0.308228 |
| -0.86391 | -0.552368 |
| -0.866352 | -0.512695 |
| -0.868793 | -0.253296 |
| -0.871235 | -0.219727 |
| -0.873676 | -0.439453 |
| -0.876117 | -0.564575 |
| -0.878559 | -0.408936 |
| -0.881 | -0.186157 |
| -0.883442 | -0.268555 |
| -0.885883 | -0.500488 |
| -0.888324 | -0.558472 |
| -0.890766 | -0.320435 |
| -0.893207 | -0.201416 |
| -0.895649 | -0.396729 |
| -0.89809 | -0.616455 |
| -0.900531 | -0.45166 |
| -0.902973 | -0.20752 |
| -0.905414 | -0.262451 |
| -0.907856 | -0.521851 |
| -0.910297 | -0.592041 |
| -0.912738 | -0.341797 |
| -0.91518 | -0.238037 |
| -0.917621 | -0.38147 |
| -0.920063 | -0.653076 |
| -0.922504 | -0.576782 |
| -0.924945 | -0.280762 |
| -0.927387 | -0.27771 |
| -0.929828 | -0.509644 |
| -0.93227 | -0.646973 |
| -0.934711 | -0.427246 |
| -0.937152 | -0.241089 |
| -0.939594 | -0.405884 |
| -0.942035 | -0.665283 |
| -0.944477 | -0.616455 |
| -0.946918 | -0.335693 |
| -0.94936 | -0.302124 |
| -0.951801 | -0.515747 |
| -0.954242 | -0.668335 |
| -0.956684 | -0.491333 |
| -0.959125 | -0.259399 |
| -0.961567 | -0.350952 |
| -0.964008 | -0.631714 |
| -0.966449 | -0.67749 |
| -0.968891 | -0.39978 |
| -0.971332 | -0.292969 |
| -0.973774 | -0.506592 |
| -0.976215 | -0.701904 |
| -0.978656 | -0.552368 |
| -0.981098 | -0.274658 |
| -0.983539 | -0.341797 |
| -0.985981 | -0.610352 |
| -0.988422 | -0.689697 |
| -0.990863 | -0.439453 |
| -0.993305 | -0.341797 |
| -0.995746 | -0.497437 |
| -0.998188 | -0.756836 |
| -1.00063 | -0.680542 |
| -1.00307 | -0.384521 |
| -1.00551 | -0.384521 |
| -1.00795 | -0.628662 |
| -1.01039 | -0.756836 |
| -1.01284 | -0.524902 |
| -1.01528 | -0.344849 |
| -1.01772 | -0.527954 |
| -1.02016 | -0.808716 |
| -1.0226 | -0.753784 |
| -1.02504 | -0.463867 |
| -1.02748 | -0.439453 |
| -1.02993 | -0.671387 |
| -1.03237 | -0.805664 |
| -1.03481 | -0.610352 |
| -1.03725 | -0.405884 |
| -1.03969 | -0.506592 |
| -1.04213 | -0.787354 |
| -1.04457 | -0.808716 |
| -1.04702 | -0.524902 |
| -1.04946 | -0.448608 |
| -1.0519 | -0.680542 |
| -1.05434 | -0.872803 |
| -1.05678 | -0.723267 |
| -1.05922 | -0.448608 |
| -1.06166 | -0.531006 |
| -1.06411 | -0.796509 |
| -1.06655 | -0.872803 |
| -1.06899 | -0.6073 |
| -1.07143 | -0.50354 |
| -1.07387 | -0.692749 |
| -1.07631 | -0.958252 |
| -1.07875 | -0.848389 |
| -1.0812 | -0.570679 |
| -1.08364 | -0.601196 |
| -1.08608 | -0.872803 |

**Figure S7**. CV of **5g** (5mM) recorded in MeCN/TBAP (0.15 M) at a scan rate of 100 mV s⁻¹. Potentials referenced to Fc/Fc.

# 7. UV-vis Data

## 7.1 Quinolin-4-ones (2)

| Wavelength (nm) | Absorption (a.u.) |
| --- | --- |
| 800.015 | -2.95608e-05 |
| 794.986 | -0.000206262 |
| 790.016 | 0.000265205 |
| 785.002 | 0.00110098 |
| 780.012 | -0.00032304 |
| 775.013 | 2.3764e-05 |
| 770.005 | -0.000335818 |
| 764.987 | -0.00139513 |
| 759.995 | -0.00201273 |
| 754.994 | -0.00264498 |
| 749.983 | -0.00280668 |
| 744.999 | -0.00379256 |
| 740.006 | -0.00566091 |
| 735.004 | -0.00735927 |
| 729.993 | -0.00903057 |
| 725.009 | -0.01012 |
| 720.017 | -0.0114455 |
| 715.016 | -0.0110766 |
| 710.007 | -0.00897276 |
| 704.989 | -0.00589101 |
| 699.999 | -0.00177783 |
| 695 | 0.00180635 |
| 689.994 | 0.0041782 |
| 685.016 | 0.00594449 |
| 679.994 | 0.00863447 |
| 675 | 0.00915609 |
| 669.998 | 0.0103527 |
| 664.988 | 0.00997616 |
| 660.008 | 0.0096904 |
| 654.983 | 0.00937404 |
| 649.988 | 0.00913999 |
| 644.985 | 0.0089416 |
| 640.011 | 0.00974197 |
| 634.994 | 0.0097839 |
| 630.006 | 0.00950229 |
| 625.011 | 0.009325 |
| 620.009 | 0.00974427 |
| 615.001 | 0.00986757 |
| 609.985 | 0.0095948 |
| 605 | 0.0099199 |
| 600.008 | 0.00975481 |
| 595.009 | 0.00933963 |
| 590.004 | 0.00937266 |
| 584.992 | 0.00892234 |
| 580.011 | 0.00862275 |
| 574.987 | 0.00785048 |
| 569.994 | 0.00700739 |
| 564.994 | 0.00628176 |
| 559.988 | 0.00579732 |
| 555.014 | 0.00418596 |
| 549.996 | 0.00316767 |
| 545.01 | 0.00225505 |
| 540.018 | 0.00138885 |
| 534.983 | 0.00188228 |
| 530.017 | 0.00285584 |
| 525.008 | 0.00440638 |
| 519.994 | 0.00671903 |
| 515.012 | 0.00877553 |
| 509.986 | 0.0106478 |
| 504.993 | 0.0120696 |
| 499.995 | 0.0127378 |
| 494.991 | 0.0132915 |
| 489.982 | 0.0133598 |
| 485.006 | 0.0125271 |
| 479.987 | 0.0119204 |
| 475.001 | 0.0123808 |
| 470.01 | 0.0144518 |
| 465.014 | 0.0168595 |
| 460.014 | 0.0193866 |
| 455.008 | 0.0211346 |
| 449.998 | 0.0224259 |
| 444.984 | 0.0234275 |
| 440.003 | 0.0240985 |
| 435.017 | 0.0244006 |
| 429.989 | 0.0243965 |
| 424.995 | 0.0246753 |
| 419.996 | 0.0245469 |
| 414.994 | 0.0243325 |
| 409.987 | 0.0241717 |
| 405.014 | 0.0239598 |
| 399.999 | 0.0240948 |
| 395.018 | 0.0244085 |
| 389.995 | 0.025018 |
| 385.006 | 0.0263558 |
| 380.013 | 0.0287494 |
| 375.017 | 0.0315945 |
| 370.017 | 0.0347737 |
| 365.013 | 0.0383352 |
| 360.006 | 0.0427599 |
| 354.995 | 0.0511155 |
| 350.019 | 0.0760073 |
| 345.001 | 0.174087 |
| 340.019 | 0.378904 |
| 334.994 | 0.510823 |
| 330.005 | 0.448882 |
| 325.012 | 0.45691 |
| 320.016 | 0.434474 |
| 315.018 | 0.341785 |
| 310.016 | 0.285809 |
| 305.011 | 0.233105 |
| 300.003 | 0.193959 |
| 294.992 | 0.187596 |
| 290.017 | 0.192849 |
| 285 | 0.182342 |
| 279.981 | 0.195699 |
| 274.998 | 0.210298 |
| 270.012 | 0.259092 |
| 264.984 | 0.361662 |
| 259.993 | 0.549846 |
| 255 | 0.826904 |
| 250.004 | 0.848577 |
| 245.006 | 0.853258 |
| 240.005 | 0.78436 |
| 235.002 | 0.753127 |
| 229.997 | 0.756213 |
| 224.989 | 0.844563 |
| 220.019 | 1.05552 |
| 215.007 | 1.38491 |
| 209.993 | 1.60225 |
| 205.017 | 1.62268 |
| 199.999 | 1.72971 |
| 195.019 | 2.16651 |
| 189.997 | 2.94864 |

**Figure S8.** UV-vis absorption spectrum for **2a** (2mM in MeCN)

| Wavelength (nm) | Absorption (a.u.) |
| --- | --- |
| 800.015 | 0.000122976 |
| 794.986 | 0.00039621 |
| 790.016 | 0.000325664 |
| 785.002 | 0.00110344 |
| 780.012 | 0.000522538 |
| 775.013 | -0.000400582 |
| 770.005 | -0.000520152 |
| 764.987 | -0.000913435 |
| 759.995 | -0.00197351 |
| 754.994 | -0.003104 |
| 749.983 | -0.00405135 |
| 744.999 | -0.00423202 |
| 740.006 | -0.00587589 |
| 735.004 | -0.00702919 |
| 729.993 | -0.00898508 |
| 725.009 | -0.0102399 |
| 720.017 | -0.011048 |
| 715.016 | -0.0110662 |
| 710.007 | -0.00858656 |
| 704.989 | -0.00567737 |
| 699.999 | -0.00197289 |
| 695 | 0.00170555 |
| 689.994 | 0.00415651 |
| 685.016 | 0.00621947 |
| 679.994 | 0.00800713 |
| 675 | 0.00921391 |
| 669.998 | 0.0105763 |
| 664.988 | 0.0097215 |
| 660.008 | 0.00950536 |
| 654.983 | 0.00967632 |
| 649.988 | 0.00932831 |
| 644.985 | 0.00972746 |
| 640.011 | 0.00929504 |
| 634.994 | 0.0101802 |
| 630.006 | 0.00976283 |
| 625.011 | 0.00999873 |
| 620.009 | 0.00978446 |
| 615.001 | 0.00957321 |
| 609.985 | 0.00978449 |
| 605 | 0.0101346 |
| 600.008 | 0.010128 |
| 595.009 | 0.00953259 |
| 590.004 | 0.00970131 |
| 584.992 | 0.00902455 |
| 580.011 | 0.00872312 |
| 574.987 | 0.00811607 |
| 569.994 | 0.00721884 |
| 564.994 | 0.00642592 |
| 559.988 | 0.00533127 |
| 555.014 | 0.00438904 |
| 549.996 | 0.00322079 |
| 545.01 | 0.00236649 |
| 540.018 | 0.00166088 |
| 534.983 | 0.00205614 |
| 530.017 | 0.00307874 |
| 525.008 | 0.00464331 |
| 519.994 | 0.00690459 |
| 515.012 | 0.0090614 |
| 509.986 | 0.0108408 |
| 504.993 | 0.0123836 |
| 499.995 | 0.0132748 |
| 494.991 | 0.0135257 |
| 489.982 | 0.0133693 |
| 485.006 | 0.0129131 |
| 479.987 | 0.0127329 |
| 475.001 | 0.0129425 |
| 470.01 | 0.0149441 |
| 465.014 | 0.017461 |
| 460.014 | 0.019815 |
| 455.008 | 0.0214562 |
| 449.998 | 0.023164 |
| 444.984 | 0.0238937 |
| 440.003 | 0.0246085 |
| 435.017 | 0.0250916 |
| 429.989 | 0.0250575 |
| 424.995 | 0.0251132 |
| 419.996 | 0.0252099 |
| 414.994 | 0.0252449 |
| 409.987 | 0.0249279 |
| 405.014 | 0.0247871 |
| 399.999 | 0.0244847 |
| 395.018 | 0.0244065 |
| 389.995 | 0.0251237 |
| 385.006 | 0.0263126 |
| 380.013 | 0.0278568 |
| 375.017 | 0.0309106 |
| 370.017 | 0.0342377 |
| 365.013 | 0.0380003 |
| 360.006 | 0.0422629 |
| 354.995 | 0.0493826 |
| 350.019 | 0.067258 |
| 345.001 | 0.127696 |
| 340.019 | 0.236625 |
| 334.994 | 0.300608 |
| 330.005 | 0.274314 |
| 325.012 | 0.282238 |
| 320.016 | 0.26909 |
| 315.018 | 0.224592 |
| 310.016 | 0.197554 |
| 305.011 | 0.171318 |
| 300.003 | 0.15385 |
| 294.992 | 0.153372 |
| 290.017 | 0.155533 |
| 285 | 0.153042 |
| 279.981 | 0.160328 |
| 274.998 | 0.171815 |
| 270.012 | 0.205915 |
| 264.984 | 0.279217 |
| 259.993 | 0.390577 |
| 255 | 0.521582 |
| 250.004 | 0.528123 |
| 245.006 | 0.525443 |
| 240.005 | 0.491743 |
| 235.002 | 0.472742 |
| 229.997 | 0.461337 |
| 224.989 | 0.491644 |
| 220.019 | 0.636476 |
| 215.007 | 0.836711 |
| 209.993 | 0.968133 |
| 205.017 | 0.983075 |
| 199.999 | 1.05504 |
| 195.019 | 1.39859 |
| 189.997 | 2.08669 |

**Figure S9.** UV-vis absorption spectrum for **2b** (2 mM in MeCN)

| Wavelength (nm) | Absorption (a.u.) |
| --- | --- |
| 800.015 | 0.00321951 |
| 794.986 | 0.00392449 |
| 790.016 | 0.00299183 |
| 785.002 | 0.00390088 |
| 780.012 | 0.00181693 |
| 775.013 | 0.00245574 |
| 770.005 | 0.00202925 |
| 764.987 | 0.00195396 |
| 759.995 | 0.00157964 |
| 754.994 | 0.00116602 |
| 749.983 | 0.00011557 |
| 744.999 | -0.000987462 |
| 740.006 | -0.00267025 |
| 735.004 | -0.00462159 |
| 729.993 | -0.00544286 |
| 725.009 | -0.00700326 |
| 720.017 | -0.0081707 |
| 715.016 | -0.00794613 |
| 710.007 | -0.00606606 |
| 704.989 | -0.0025982 |
| 699.999 | 0.00156535 |
| 695 | 0.0045661 |
| 689.994 | 0.00723393 |
| 685.016 | 0.0100374 |
| 679.994 | 0.0111998 |
| 675 | 0.0125099 |
| 669.998 | 0.0140233 |
| 664.988 | 0.0129714 |
| 660.008 | 0.0127041 |
| 654.983 | 0.0126959 |
| 649.988 | 0.012732 |
| 644.985 | 0.0128074 |
| 640.011 | 0.012973 |
| 634.994 | 0.0131322 |
| 630.006 | 0.012718 |
| 625.011 | 0.0131335 |
| 620.009 | 0.013167 |
| 615.001 | 0.0131463 |
| 609.985 | 0.0132388 |
| 605 | 0.0132391 |
| 600.008 | 0.0129962 |
| 595.009 | 0.0126554 |
| 590.004 | 0.0125794 |
| 584.992 | 0.0123311 |
| 580.011 | 0.0115365 |
| 574.987 | 0.0110871 |
| 569.994 | 0.0106824 |
| 564.994 | 0.00979232 |
| 559.988 | 0.00869661 |
| 555.014 | 0.00760921 |
| 549.996 | 0.00634651 |
| 545.01 | 0.00567367 |
| 540.018 | 0.00485063 |
| 534.983 | 0.00532406 |
| 530.017 | 0.00626204 |
| 525.008 | 0.00793191 |
| 519.994 | 0.0101368 |
| 515.012 | 0.012302 |
| 509.986 | 0.0143986 |
| 504.993 | 0.0155008 |
| 499.995 | 0.0163364 |
| 494.991 | 0.0167555 |
| 489.982 | 0.0165559 |
| 485.006 | 0.0158613 |
| 479.987 | 0.0155568 |
| 475.001 | 0.0160327 |
| 470.01 | 0.01812 |
| 465.014 | 0.0206049 |
| 460.014 | 0.0228799 |
| 455.008 | 0.0247245 |
| 449.998 | 0.0262856 |
| 444.984 | 0.027119 |
| 440.003 | 0.0276149 |
| 435.017 | 0.02777 |
| 429.989 | 0.0280679 |
| 424.995 | 0.0280733 |
| 419.996 | 0.0282395 |
| 414.994 | 0.0279492 |
| 409.987 | 0.0277203 |
| 405.014 | 0.0274113 |
| 399.999 | 0.0273201 |
| 395.018 | 0.0274995 |
| 389.995 | 0.0276374 |
| 385.006 | 0.028964 |
| 380.013 | 0.0307422 |
| 375.017 | 0.0332353 |
| 370.017 | 0.036671 |
| 365.013 | 0.040299 |
| 360.006 | 0.0443192 |
| 354.995 | 0.0490961 |
| 350.019 | 0.0599433 |
| 345.001 | 0.0918942 |
| 340.019 | 0.163103 |
| 334.994 | 0.234306 |
| 330.005 | 0.220001 |
| 325.012 | 0.222957 |
| 320.016 | 0.226021 |
| 315.018 | 0.195193 |
| 310.016 | 0.17963 |
| 305.011 | 0.167545 |
| 300.003 | 0.163081 |
| 294.992 | 0.169919 |
| 290.017 | 0.181582 |
| 285 | 0.189317 |
| 279.981 | 0.191907 |
| 274.998 | 0.196383 |
| 270.012 | 0.203233 |
| 264.984 | 0.227351 |
| 259.993 | 0.278863 |
| 255 | 0.365318 |
| 250.004 | 0.392803 |
| 245.006 | 0.416199 |
| 240.005 | 0.432152 |
| 235.002 | 0.506427 |
| 229.997 | 0.651465 |
| 224.989 | 0.720879 |
| 220.019 | 0.800834 |
| 215.007 | 0.931158 |
| 209.993 | 0.971595 |
| 205.017 | 0.893199 |
| 199.999 | 0.867585 |
| 195.019 | 1.03647 |
| 189.997 | 1.56059 |

**Figure S10.** UV-vis absorption spectrum for **2c** (2 mM in MeCN)

## 7.2 4-amino-quinolinium salts (5)

| Wavelength (nm) | absorption (a.u.) |
| --- | --- |
| 799.991 | -9e-05 |
| 798.985 | 0.00011 |
| 798.014 | 0.00055 |
| 797.008 | 0.00051 |
| 796.002 | -0.00015 |
| 794.995 | 0.00041 |
| 793.987 | 0.00011 |
| 793.015 | 0.00014 |
| 792.006 | -0.00045 |
| 790.998 | -0.00031 |
| 789.989 | 0.00035 |
| 789.014 | 0.0006 |
| 788.005 | -0.00019 |
| 786.995 | 0.00028 |
| 785.984 | -0.00028 |
| 785.008 | 3e-05 |
| 783.997 | 5e-05 |
| 782.985 | 0.00014 |
| 782.008 | 0.00013 |
| 780.995 | -0.00079 |
| 779.983 | 0.00016 |
| 779.004 | -7e-05 |
| 777.991 | -0.00036 |
| 777.012 | -0.00023 |
| 775.997 | 0.00016 |
| 775.018 | -0.00023 |
| 774.002 | -0.0006 |
| 772.987 | -0.00079 |
| 772.006 | -9e-05 |
| 770.99 | -0.00088 |
| 770.008 | -0.0008 |
| 768.991 | -0.00028 |
| 768.008 | -0.00095 |
| 766.99 | -0.00119 |
| 766.007 | -0.00062 |
| 764.989 | -0.00123 |
| 764.005 | -0.00159 |
| 762.985 | -0.00112 |
| 762.001 | -0.00099 |
| 761.016 | -0.00135 |
| 759.995 | -0.00147 |
| 759.01 | -0.00216 |
| 757.988 | -0.00185 |
| 757.002 | -0.00192 |
| 756.015 | -0.00248 |
| 754.993 | -0.00218 |
| 754.005 | -0.00242 |
| 753.017 | -0.00287 |
| 751.994 | -0.00245 |
| 751.005 | -0.00334 |
| 750.016 | -0.00346 |
| 748.992 | -0.00325 |
| 748.002 | -0.00401 |
| 747.012 | -0.00395 |
| 745.986 | -0.00372 |
| 744.996 | -0.00469 |
| 744.004 | -0.00451 |
| 743.013 | -0.00489 |
| 741.986 | -0.00503 |
| 740.994 | -0.00528 |
| 740.001 | -0.00582 |
| 739.008 | -0.00579 |
| 738.015 | -0.00659 |
| 736.986 | -0.00631 |
| 735.992 | -0.00669 |
| 734.998 | -0.00689 |
| 734.003 | -0.00746 |
| 733.008 | -0.00775 |
| 732.013 | -0.00816 |
| 731.017 | -0.00843 |
| 729.986 | -0.00871 |
| 728.99 | -0.00929 |
| 727.993 | -0.00927 |
| 726.996 | -0.00998 |
| 725.998 | -0.01001 |
| 725.001 | -0.01064 |
| 724.003 | -0.01052 |
| 723.004 | -0.01103 |
| 722.006 | -0.01093 |
| 721.007 | -0.01121 |
| 720.007 | -0.01128 |
| 719.007 | -0.0114 |
| 718.007 | -0.01123 |
| 717.007 | -0.01127 |
| 716.006 | -0.011 |
| 715.005 | -0.01099 |
| 714.003 | -0.01053 |
| 713.002 | -0.01027 |
| 712 | -0.00973 |
| 710.997 | -0.0093 |
| 709.994 | -0.00855 |
| 708.991 | -0.00807 |
| 707.988 | -0.0073 |
| 706.984 | -0.00662 |
| 706.016 | -0.00606 |
| 705.011 | -0.00522 |
| 704.006 | -0.00476 |
| 703.001 | -0.00382 |
| 701.996 | -0.00322 |
| 700.99 | -0.00224 |
| 699.984 | -0.00179 |
| 699.013 | -0.00103 |
| 698.007 | -0.00022 |
| 697 | 0.00046 |
| 695.992 | 0.001 |
| 694.984 | 0.0017 |
| 694.012 | 0.00224 |
| 693.004 | 0.00302 |
| 691.995 | 0.00357 |
| 690.986 | 0.00403 |
| 690.013 | 0.00459 |
| 689.003 | 0.0051 |
| 687.993 | 0.00543 |
| 686.983 | 0.00608 |
| 686.008 | 0.00641 |
| 684.997 | 0.00694 |
| 683.986 | 0.00715 |
| 683.01 | 0.00757 |
| 681.998 | 0.00791 |
| 680.986 | 0.00824 |
| 680.01 | 0.00839 |
| 678.997 | 0.00884 |
| 677.984 | 0.00906 |
| 677.006 | 0.0093 |
| 675.992 | 0.00946 |
| 675.015 | 0.00988 |
| 674 | 0.00999 |
| 672.985 | 0.01027 |
| 672.007 | 0.01045 |
| 670.991 | 0.01061 |
| 670.012 | 0.01088 |
| 668.996 | 0.01087 |
| 668.016 | 0.01067 |
| 666.999 | 0.01027 |
| 665.982 | 0.01012 |
| 665.001 | 0.01024 |
| 663.984 | 0.01019 |
| 663.002 | 0.0099 |
| 661.984 | 0.00999 |
| 661.002 | 0.0098 |
| 659.983 | 0.00987 |
| 659 | 0.00985 |
| 658.017 | 0.00999 |
| 656.997 | 0.00974 |
| 656.014 | 0.01002 |
| 654.993 | 0.01002 |
| 654.009 | 0.00976 |
| 652.988 | 0.01001 |
| 652.004 | 0.00986 |
| 650.982 | 0.01005 |
| 649.997 | 0.01003 |
| 649.011 | 0.01013 |
| 647.989 | 0.01008 |
| 647.002 | 0.01009 |
| 646.016 | 0.00997 |
| 644.993 | 0.01009 |
| 644.006 | 0.01009 |
| 643.018 | 0.01042 |
| 641.994 | 0.01016 |
| 641.006 | 0.01013 |
| 640.018 | 0.01017 |
| 638.993 | 0.01023 |
| 638.004 | 0.01014 |
| 637.015 | 0.01012 |
| 635.989 | 0.01024 |
| 634.999 | 0.01025 |
| 634.009 | 0.01018 |
| 632.983 | 0.01034 |
| 631.992 | 0.01014 |
| 631.001 | 0.01025 |
| 630.01 | 0.01011 |
| 628.982 | 0.01012 |
| 627.991 | 0.01024 |
| 626.999 | 0.01006 |
| 626.007 | 0.01011 |
| 625.014 | 0.01016 |
| 623.985 | 0.01031 |
| 622.992 | 0.01031 |
| 621.999 | 0.01029 |
| 621.005 | 0.01036 |
| 620.011 | 0.01028 |
| 619.017 | 0.01019 |
| 617.986 | 0.0102 |
| 616.991 | 0.01022 |
| 615.996 | 0.01017 |
| 615.001 | 0.01033 |
| 614.006 | 0.01034 |
| 613.01 | 0.01025 |
| 612.014 | 0.01013 |
| 611.018 | 0.01022 |
| 609.985 | 0.0102 |
| 608.988 | 0.01022 |
| 607.991 | 0.0103 |
| 606.993 | 0.01032 |
| 605.996 | 0.01039 |
| 604.998 | 0.01007 |
| 604 | 0.01002 |
| 603.001 | 0.01007 |
| 602.003 | 0.01015 |
| 601.004 | 0.01008 |

**Figure S11.** UV-vis absorption spectrum for **5c** (2 mM in MeCN)

| Wavelength (nm) | absorption (a.u.) |
| --- | --- |
| 799.991 | -0.00131194 |
| 798.985 | -0.000776092 |
| 798.014 | -0.000440304 |
| 797.008 | -0.000543059 |
| 796.002 | -0.00125898 |
| 794.995 | -0.000951974 |
| 793.987 | -0.00106127 |
| 793.015 | -0.000956881 |
| 792.006 | -0.00148569 |
| 790.998 | -0.00107847 |
| 789.989 | -0.00131421 |
| 789.014 | -0.00103607 |
| 788.005 | -0.00126393 |
| 786.995 | -0.00065039 |
| 785.984 | -0.000671532 |
| 785.008 | -0.00108095 |
| 783.997 | -0.000855206 |
| 782.985 | -0.00122093 |
| 782.008 | -0.000933996 |
| 780.995 | -0.00165964 |
| 779.983 | -0.0012613 |
| 779.004 | -0.0012598 |
| 777.991 | -0.000991439 |
| 777.012 | -0.00140076 |
| 775.997 | -0.00117033 |
| 775.018 | -0.00168094 |
| 774.002 | -0.00187403 |
| 772.987 | -0.00184815 |
| 772.006 | -0.00132773 |
| 770.99 | -0.00199644 |
| 770.008 | -0.00237835 |
| 768.991 | -0.00157479 |
| 768.008 | -0.00211841 |
| 766.99 | -0.00229941 |
| 766.007 | -0.00213587 |
| 764.989 | -0.00250263 |
| 764.005 | -0.00266577 |
| 762.985 | -0.00216302 |
| 762.001 | -0.00224827 |
| 761.016 | -0.00268965 |
| 759.995 | -0.00240348 |
| 759.01 | -0.00331763 |
| 757.988 | -0.00308858 |
| 757.002 | -0.00336495 |
| 756.015 | -0.00378748 |
| 754.993 | -0.00354076 |
| 754.005 | -0.00368683 |
| 753.017 | -0.0042659 |
| 751.994 | -0.00394645 |
| 751.005 | -0.00431163 |
| 750.016 | -0.00463256 |
| 748.992 | -0.00440379 |
| 748.002 | -0.00480622 |
| 747.012 | -0.00504204 |
| 745.986 | -0.00519942 |
| 744.996 | -0.00581915 |
| 744.004 | -0.00547532 |
| 743.013 | -0.0060244 |
| 741.986 | -0.00641401 |
| 740.994 | -0.00651592 |
| 740.001 | -0.00693576 |
| 739.008 | -0.0069943 |
| 738.015 | -0.00786092 |
| 736.986 | -0.00739057 |
| 735.992 | -0.00807838 |
| 734.998 | -0.00806567 |
| 734.003 | -0.00859098 |
| 733.008 | -0.00896039 |
| 732.013 | -0.00915027 |
| 731.017 | -0.00991404 |
| 729.986 | -0.00994309 |
| 728.99 | -0.0105127 |
| 727.993 | -0.0104921 |
| 726.996 | -0.0111393 |
| 725.998 | -0.0113067 |
| 725.001 | -0.0115831 |
| 724.003 | -0.0118977 |
| 723.004 | -0.0121605 |
| 722.006 | -0.0122232 |
| 721.007 | -0.0124208 |
| 720.007 | -0.0123753 |
| 719.007 | -0.0125445 |
| 718.007 | -0.0124892 |
| 717.007 | -0.0125189 |
| 716.006 | -0.0120903 |
| 715.005 | -0.012166 |
| 714.003 | -0.0117243 |
| 713.002 | -0.0116515 |
| 712 | -0.0108428 |
| 710.997 | -0.0105504 |
| 709.994 | -0.0100567 |
| 708.991 | -0.00939044 |
| 707.988 | -0.0086839 |
| 706.984 | -0.00800438 |
| 706.016 | -0.00730006 |
| 705.011 | -0.00659766 |
| 704.006 | -0.00578844 |
| 703.001 | -0.00493333 |
| 701.996 | -0.00442357 |
| 700.99 | -0.00354687 |
| 699.984 | -0.00311824 |
| 699.013 | -0.00228726 |
| 698.007 | -0.00160151 |
| 697 | -0.000846474 |
| 695.992 | -0.00033437 |
| 694.984 | 0.000386624 |
| 694.012 | 0.0010155 |
| 693.004 | 0.00142786 |
| 691.995 | 0.00229528 |
| 690.986 | 0.0026464 |
| 690.013 | 0.00331489 |
| 689.003 | 0.00368547 |
| 687.993 | 0.004342 |
| 686.983 | 0.00440081 |
| 686.008 | 0.00504006 |
| 684.997 | 0.00553535 |
| 683.986 | 0.00585535 |
| 683.01 | 0.00610027 |
| 681.998 | 0.00648285 |
| 680.986 | 0.00677563 |
| 680.01 | 0.00712434 |
| 678.997 | 0.00735065 |
| 677.984 | 0.00761838 |
| 677.006 | 0.00807593 |
| 675.992 | 0.00814985 |
| 675.015 | 0.00847134 |
| 674 | 0.00849098 |
| 672.985 | 0.0089518 |
| 672.007 | 0.00900558 |
| 670.991 | 0.00924501 |
| 670.012 | 0.00946771 |
| 668.996 | 0.00944168 |
| 668.016 | 0.00933611 |
| 666.999 | 0.00860833 |
| 665.982 | 0.00870112 |
| 665.001 | 0.0087615 |
| 663.984 | 0.00859267 |
| 663.002 | 0.00863656 |
| 661.984 | 0.00842401 |
| 661.002 | 0.00852406 |
| 659.983 | 0.00860149 |
| 659 | 0.00834717 |
| 658.017 | 0.00841177 |
| 656.997 | 0.00842116 |
| 656.014 | 0.0085588 |
| 654.993 | 0.00833777 |
| 654.009 | 0.00842357 |
| 652.988 | 0.00857287 |
| 652.004 | 0.00820794 |
| 650.982 | 0.00849259 |
| 649.997 | 0.00853018 |
| 649.011 | 0.00847662 |
| 647.989 | 0.00841121 |
| 647.002 | 0.00859331 |
| 646.016 | 0.00842544 |
| 644.993 | 0.00835363 |
| 644.006 | 0.00857205 |
| 643.018 | 0.00849945 |
| 641.994 | 0.00854515 |
| 641.006 | 0.0087732 |
| 640.018 | 0.00848361 |
| 638.993 | 0.00853493 |
| 638.004 | 0.00867757 |
| 637.015 | 0.00853311 |
| 635.989 | 0.00866048 |
| 634.999 | 0.00850368 |
| 634.009 | 0.00861765 |
| 632.983 | 0.0086594 |
| 631.992 | 0.00867548 |
| 631.001 | 0.00867598 |
| 630.01 | 0.00876678 |
| 628.982 | 0.00879457 |
| 627.991 | 0.00882273 |
| 626.999 | 0.0085244 |
| 626.007 | 0.00872011 |
| 625.014 | 0.00870754 |
| 623.985 | 0.00874972 |
| 622.992 | 0.00891346 |
| 621.999 | 0.00870997 |
| 621.005 | 0.00890363 |
| 620.011 | 0.008982 |
| 619.017 | 0.00870342 |
| 617.986 | 0.00880054 |
| 616.991 | 0.00884447 |
| 615.996 | 0.00890878 |
| 615.001 | 0.00878406 |
| 614.006 | 0.00862549 |
| 613.01 | 0.00874381 |
| 612.014 | 0.00878089 |
| 611.018 | 0.00878734 |
| 609.985 | 0.00875416 |
| 608.988 | 0.00877466 |
| 607.991 | 0.00882609 |
| 606.993 | 0.00878049 |
| 605.996 | 0.00891235 |
| 604.998 | 0.00877489 |
| 604 | 0.0086463 |
| 603.001 | 0.00870316 |
| 602.003 | 0.00861116 |
| 601.004 | 0.00857269 |

**Figure S12.** UV-vis absorption spectrum for **5f** (2 mM in MeCN)

| Wavelength (nm) | absorption (a.u.) |
| --- | --- |
| 799.991 | -0.00094097 |
| 798.985 | -0.00100492 |
| 798.014 | -0.000863835 |
| 797.008 | -0.000679286 |
| 796.002 | -0.00116703 |
| 794.995 | -0.000737434 |
| 793.987 | -0.00039484 |
| 793.015 | -0.000507017 |
| 792.006 | -0.00095559 |
| 790.998 | -0.00113656 |
| 789.989 | -0.000452924 |
| 789.014 | -0.000553917 |
| 788.005 | -0.00114049 |
| 786.995 | -0.000978629 |
| 785.984 | -0.000494916 |
| 785.008 | -0.000997947 |
| 783.997 | -0.00120513 |
| 782.985 | -0.000760278 |
| 782.008 | -0.000894009 |
| 780.995 | -0.00105466 |
| 779.983 | -0.000854483 |
| 779.004 | -0.000791286 |
| 777.991 | -0.00143647 |
| 777.012 | -0.00174339 |
| 775.997 | -0.00101628 |
| 775.018 | -0.00111033 |
| 774.002 | -0.00168743 |
| 772.987 | -0.00178608 |
| 772.006 | -0.0013636 |
| 770.99 | -0.00195485 |
| 770.008 | -0.00192856 |
| 768.991 | -0.00159568 |
| 768.008 | -0.00215019 |
| 766.99 | -0.00199459 |
| 766.007 | -0.00201767 |
| 764.989 | -0.00240126 |
| 764.005 | -0.0025823 |
| 762.985 | -0.00189635 |
| 762.001 | -0.0020388 |
| 761.016 | -0.00241439 |
| 759.995 | -0.00240579 |
| 759.01 | -0.00309228 |
| 757.988 | -0.00295907 |
| 757.002 | -0.00318485 |
| 756.015 | -0.00356751 |
| 754.993 | -0.00357475 |
| 754.005 | -0.003552 |
| 753.017 | -0.00405412 |
| 751.994 | -0.00360453 |
| 751.005 | -0.00407756 |
| 750.016 | -0.00437739 |
| 748.992 | -0.00415449 |
| 748.002 | -0.00487472 |
| 747.012 | -0.00471435 |
| 745.986 | -0.00504475 |
| 744.996 | -0.00563766 |
| 744.004 | -0.00545099 |
| 743.013 | -0.00593509 |
| 741.986 | -0.00598626 |
| 740.994 | -0.00641237 |
| 740.001 | -0.00675887 |
| 739.008 | -0.00662958 |
| 738.015 | -0.00729757 |
| 736.986 | -0.00756364 |
| 735.992 | -0.0079051 |
| 734.998 | -0.00796351 |
| 734.003 | -0.00843248 |
| 733.008 | -0.0086263 |
| 732.013 | -0.00907438 |
| 731.017 | -0.00966991 |
| 729.986 | -0.00972409 |
| 728.99 | -0.0103162 |
| 727.993 | -0.0103285 |
| 726.996 | -0.0109243 |
| 725.998 | -0.011013 |
| 725.001 | -0.0115468 |
| 724.003 | -0.0116657 |
| 723.004 | -0.0119796 |
| 722.006 | -0.0119435 |
| 721.007 | -0.0121966 |
| 720.007 | -0.012129 |
| 719.007 | -0.012303 |
| 718.007 | -0.0121957 |
| 717.007 | -0.0122781 |
| 716.006 | -0.0119557 |
| 715.005 | -0.0118874 |
| 714.003 | -0.0116645 |
| 713.002 | -0.0111997 |
| 712 | -0.0108486 |
| 710.997 | -0.0101111 |
| 709.994 | -0.00963751 |
| 708.991 | -0.00919539 |
| 707.988 | -0.00854001 |
| 706.984 | -0.00766244 |
| 706.016 | -0.00703892 |
| 705.011 | -0.00637993 |
| 704.006 | -0.00545651 |
| 703.001 | -0.00474016 |
| 701.996 | -0.00413331 |
| 700.99 | -0.00328171 |
| 699.984 | -0.00272052 |
| 699.013 | -0.00207724 |
| 698.007 | -0.00142672 |
| 697 | -0.000706526 |
| 695.992 | 5.26e-05 |
| 694.984 | 0.00069671 |
| 694.012 | 0.00144313 |
| 693.004 | 0.00185888 |
| 691.995 | 0.00258436 |
| 690.986 | 0.00293235 |
| 690.013 | 0.00355078 |
| 689.003 | 0.00386609 |
| 687.993 | 0.00465362 |
| 686.983 | 0.00485338 |
| 686.008 | 0.00525648 |
| 684.997 | 0.00570651 |
| 683.986 | 0.00614855 |
| 683.01 | 0.00638284 |
| 681.998 | 0.00681481 |
| 680.986 | 0.00709889 |
| 680.01 | 0.00745442 |
| 678.997 | 0.00781611 |
| 677.984 | 0.00799932 |
| 677.006 | 0.0083155 |
| 675.992 | 0.0082034 |
| 675.015 | 0.00869027 |
| 674 | 0.00878564 |
| 672.985 | 0.00909613 |
| 672.007 | 0.00921611 |
| 670.991 | 0.00967198 |
| 670.012 | 0.00987462 |
| 668.996 | 0.00963913 |
| 668.016 | 0.00951277 |
| 666.999 | 0.00906875 |
| 665.982 | 0.00911088 |
| 665.001 | 0.00908032 |
| 663.984 | 0.00892564 |
| 663.002 | 0.00876969 |
| 661.984 | 0.00865457 |
| 661.002 | 0.0086642 |
| 659.983 | 0.0086224 |
| 659 | 0.00856516 |
| 658.017 | 0.00857836 |
| 656.997 | 0.00846107 |
| 656.014 | 0.00869986 |
| 654.993 | 0.00859357 |
| 654.009 | 0.00885689 |
| 652.988 | 0.00848084 |
| 652.004 | 0.00846477 |
| 650.982 | 0.00862621 |
| 649.997 | 0.00856529 |
| 649.011 | 0.00883327 |
| 647.989 | 0.00871858 |
| 647.002 | 0.00875168 |
| 646.016 | 0.00884022 |
| 644.993 | 0.00870606 |
| 644.006 | 0.00872106 |
| 643.018 | 0.00874264 |
| 641.994 | 0.00883533 |
| 641.006 | 0.00891338 |
| 640.018 | 0.00888696 |
| 638.993 | 0.00886846 |
| 638.004 | 0.00911421 |
| 637.015 | 0.00906724 |
| 635.989 | 0.00894226 |
| 634.999 | 0.00886846 |
| 634.009 | 0.00900526 |
| 632.983 | 0.00905516 |
| 631.992 | 0.00900119 |
| 631.001 | 0.00896726 |
| 630.01 | 0.00903111 |
| 628.982 | 0.0088943 |
| 627.991 | 0.00906713 |
| 626.999 | 0.00908976 |
| 626.007 | 0.00922948 |
| 625.014 | 0.0090854 |
| 623.985 | 0.00896731 |
| 622.992 | 0.00910253 |
| 621.999 | 0.00930765 |
| 621.005 | 0.00918681 |
| 620.011 | 0.00924612 |
| 619.017 | 0.0092737 |
| 617.986 | 0.00939533 |
| 616.991 | 0.00925646 |
| 615.996 | 0.0091417 |
| 615.001 | 0.00917502 |
| 614.006 | 0.00909386 |
| 613.01 | 0.00924625 |
| 612.014 | 0.00916399 |
| 611.018 | 0.00912011 |
| 609.985 | 0.00907171 |
| 608.988 | 0.0092109 |
| 607.991 | 0.00939353 |
| 606.993 | 0.00924395 |
| 605.996 | 0.0092833 |
| 604.998 | 0.00911236 |
| 604 | 0.00918853 |
| 603.001 | 0.00899554 |
| 602.003 | 0.00914765 |
| 601.004 | 0.00905146 |

**Figure S13.** UV-vis absorption spectrum for **5i** (2 mM in MeCN)

| Wavelength (nm) | absorption (a.u.) |
| --- | --- |
| 799.991 | -0.00081268 |
| 798.985 | -0.000579097 |
| 798.014 | 7.06e-05 |
| 797.008 | -9.36e-05 |
| 796.002 | -0.000670498 |
| 794.995 | -0.000588352 |
| 793.987 | -0.00032573 |
| 793.015 | -0.00026049 |
| 792.006 | -0.000959309 |
| 790.998 | -0.000839395 |
| 789.989 | -0.000335353 |
| 789.014 | -0.000509499 |
| 788.005 | -0.00110186 |
| 786.995 | -0.00053251 |
| 785.984 | -0.000329093 |
| 785.008 | -0.0010232 |
| 783.997 | -0.000798417 |
| 782.985 | -0.000687866 |
| 782.008 | -0.000620199 |
| 780.995 | -0.00123306 |
| 779.983 | -0.00130337 |
| 779.004 | -0.000754903 |
| 777.991 | -0.00147893 |
| 777.012 | -0.00134125 |
| 775.997 | -0.00108565 |
| 775.018 | -0.000916276 |
| 774.002 | -0.00148595 |
| 772.987 | -0.00134476 |
| 772.006 | -0.00117188 |
| 770.99 | -0.0017966 |
| 770.008 | -0.00179644 |
| 768.991 | -0.00128845 |
| 768.008 | -0.0018847 |
| 766.99 | -0.001848 |
| 766.007 | -0.00184629 |
| 764.989 | -0.00205508 |
| 764.005 | -0.00240816 |
| 762.985 | -0.00161213 |
| 762.001 | -0.00218579 |
| 761.016 | -0.00235569 |
| 759.995 | -0.00225074 |
| 759.01 | -0.00270729 |
| 757.988 | -0.00259234 |
| 757.002 | -0.00294081 |
| 756.015 | -0.0033539 |
| 754.993 | -0.00302977 |
| 754.005 | -0.00329199 |
| 753.017 | -0.00392218 |
| 751.994 | -0.00374191 |
| 751.005 | -0.00394158 |
| 750.016 | -0.00432137 |
| 748.992 | -0.00432742 |
| 748.002 | -0.00476991 |
| 747.012 | -0.00475619 |
| 745.986 | -0.00485588 |
| 744.996 | -0.00555946 |
| 744.004 | -0.00551581 |
| 743.013 | -0.0058267 |
| 741.986 | -0.00575094 |
| 740.994 | -0.00643722 |
| 740.001 | -0.00671722 |
| 739.008 | -0.00698187 |
| 738.015 | -0.00739592 |
| 736.986 | -0.00716692 |
| 735.992 | -0.00784536 |
| 734.998 | -0.00805205 |
| 734.003 | -0.00824482 |
| 733.008 | -0.00863463 |
| 732.013 | -0.00893249 |
| 731.017 | -0.00918996 |
| 729.986 | -0.00964267 |
| 728.99 | -0.0103192 |
| 727.993 | -0.0102378 |
| 726.996 | -0.0108237 |
| 725.998 | -0.0108239 |
| 725.001 | -0.0115368 |
| 724.003 | -0.0115572 |
| 723.004 | -0.0119129 |
| 722.006 | -0.0119834 |
| 721.007 | -0.0121218 |
| 720.007 | -0.0121432 |
| 719.007 | -0.0123461 |
| 718.007 | -0.0120741 |
| 717.007 | -0.0122193 |
| 716.006 | -0.0119051 |
| 715.005 | -0.0118822 |
| 714.003 | -0.0114471 |
| 713.002 | -0.0112843 |
| 712 | -0.0106148 |
| 710.997 | -0.0102209 |
| 709.994 | -0.00950067 |
| 708.991 | -0.00902895 |
| 707.988 | -0.00817741 |
| 706.984 | -0.00751439 |
| 706.016 | -0.00686498 |
| 705.011 | -0.00631013 |
| 704.006 | -0.00541233 |
| 703.001 | -0.00485081 |
| 701.996 | -0.00413654 |
| 700.99 | -0.00317544 |
| 699.984 | -0.00269597 |
| 699.013 | -0.00187485 |
| 698.007 | -0.00138027 |
| 697 | -0.000434046 |
| 695.992 | 4.05e-05 |
| 694.984 | 0.00078967 |
| 694.012 | 0.0015064 |
| 693.004 | 0.00196234 |
| 691.995 | 0.00250791 |
| 690.986 | 0.00308025 |
| 690.013 | 0.00363519 |
| 689.003 | 0.00405284 |
| 687.993 | 0.00444438 |
| 686.983 | 0.00483155 |
| 686.008 | 0.00529128 |
| 684.997 | 0.00590951 |
| 683.986 | 0.0062283 |
| 683.01 | 0.00644954 |
| 681.998 | 0.00679914 |
| 680.986 | 0.00696506 |
| 680.01 | 0.00731653 |
| 678.997 | 0.00781073 |
| 677.984 | 0.00816349 |
| 677.006 | 0.008267 |
| 675.992 | 0.00855896 |
| 675.015 | 0.00865673 |
| 674 | 0.00890109 |
| 672.985 | 0.00921724 |
| 672.007 | 0.00925833 |
| 670.991 | 0.00966761 |
| 670.012 | 0.00981832 |
| 668.996 | 0.00978716 |
| 668.016 | 0.00941869 |
| 666.999 | 0.00907075 |
| 665.982 | 0.00919746 |
| 665.001 | 0.00904036 |
| 663.984 | 0.0087611 |
| 663.002 | 0.00872653 |
| 661.984 | 0.00883779 |
| 661.002 | 0.00876137 |
| 659.983 | 0.00877582 |
| 659 | 0.00874143 |
| 658.017 | 0.00861625 |
| 656.997 | 0.0086096 |
| 656.014 | 0.008688 |
| 654.993 | 0.00868866 |
| 654.009 | 0.00867398 |
| 652.988 | 0.00878836 |
| 652.004 | 0.00857166 |
| 650.982 | 0.00872698 |
| 649.997 | 0.00871037 |
| 649.011 | 0.00865369 |
| 647.989 | 0.00857018 |
| 647.002 | 0.0087895 |
| 646.016 | 0.00864271 |
| 644.993 | 0.00856553 |
| 644.006 | 0.00852142 |
| 643.018 | 0.00874151 |
| 641.994 | 0.00874222 |
| 641.006 | 0.00871071 |
| 640.018 | 0.00892403 |
| 638.993 | 0.00879919 |
| 638.004 | 0.00894348 |
| 637.015 | 0.00887924 |
| 635.989 | 0.00873055 |
| 634.999 | 0.0090176 |
| 634.009 | 0.00889993 |
| 632.983 | 0.00904075 |
| 631.992 | 0.00907234 |
| 631.001 | 0.00893201 |
| 630.01 | 0.0089193 |
| 628.982 | 0.0090683 |
| 627.991 | 0.00907583 |
| 626.999 | 0.00909486 |
| 626.007 | 0.00912584 |
| 625.014 | 0.00910856 |
| 623.985 | 0.00914226 |
| 622.992 | 0.00910361 |
| 621.999 | 0.00922824 |
| 621.005 | 0.00938928 |
| 620.011 | 0.00908965 |
| 619.017 | 0.00937309 |
| 617.986 | 0.00928745 |
| 616.991 | 0.00926418 |
| 615.996 | 0.00915109 |
| 615.001 | 0.00911307 |
| 614.006 | 0.0090489 |
| 613.01 | 0.00905997 |
| 612.014 | 0.00895151 |
| 611.018 | 0.0091029 |
| 609.985 | 0.00908228 |
| 608.988 | 0.00911931 |
| 607.991 | 0.0092508 |
| 606.993 | 0.0091131 |
| 605.996 | 0.00898314 |
| 604.998 | 0.00887401 |
| 604 | 0.00887724 |
| 603.001 | 0.00894831 |
| 602.003 | 0.0090319 |
| 601.004 | 0.00894181 |

**Figure S14.** UV-vis absorption spectrum for **5l** (2 mM in MeCN)

| Wavelength (nm) | absorption (a.u.) |
| --- | --- |
| 799.991 | -0.00121257 |
| 798.985 | -0.00127616 |
| 798.014 | -0.000301726 |
| 797.008 | -0.000456078 |
| 796.002 | -0.00118092 |
| 794.995 | -0.00106519 |
| 793.987 | -0.000460991 |
| 793.015 | -0.00107929 |
| 792.006 | -0.00122351 |
| 790.998 | -0.00162235 |
| 789.989 | -0.00101324 |
| 789.014 | -0.00126197 |
| 788.005 | -0.00110336 |
| 786.995 | -0.00122661 |
| 785.984 | -0.00129299 |
| 785.008 | -0.0012279 |
| 783.997 | -0.00129 |
| 782.985 | -0.00113098 |
| 782.008 | -0.00124292 |
| 780.995 | -0.00156107 |
| 779.983 | -0.00149797 |
| 779.004 | -0.00107248 |
| 777.991 | -0.00143771 |
| 777.012 | -0.00181588 |
| 775.997 | -0.00155529 |
| 775.018 | -0.00142429 |
| 774.002 | -0.00172095 |
| 772.987 | -0.00157726 |
| 772.006 | -0.001603 |
| 770.99 | -0.00175952 |
| 770.008 | -0.00196444 |
| 768.991 | -0.00184537 |
| 768.008 | -0.00205493 |
| 766.99 | -0.00244003 |
| 766.007 | -0.00198144 |
| 764.989 | -0.0023923 |
| 764.005 | -0.0026687 |
| 762.985 | -0.00219073 |
| 762.001 | -0.00275005 |
| 761.016 | -0.00269844 |
| 759.995 | -0.00259121 |
| 759.01 | -0.00340013 |
| 757.988 | -0.00294333 |
| 757.002 | -0.00322565 |
| 756.015 | -0.00411254 |
| 754.993 | -0.00362081 |
| 754.005 | -0.00366871 |
| 753.017 | -0.00409295 |
| 751.994 | -0.00405176 |
| 751.005 | -0.00444022 |
| 750.016 | -0.00470662 |
| 748.992 | -0.00436858 |
| 748.002 | -0.00531395 |
| 747.012 | -0.00504854 |
| 745.986 | -0.00535634 |
| 744.996 | -0.00571528 |
| 744.004 | -0.00558916 |
| 743.013 | -0.00619 |
| 741.986 | -0.00642201 |
| 740.994 | -0.00663784 |
| 740.001 | -0.00700454 |
| 739.008 | -0.00712286 |
| 738.015 | -0.0076777 |
| 736.986 | -0.00761604 |
| 735.992 | -0.00833711 |
| 734.998 | -0.00838053 |
| 734.003 | -0.00860336 |
| 733.008 | -0.00899751 |
| 732.013 | -0.00936951 |
| 731.017 | -0.00977456 |
| 729.986 | -0.00991106 |
| 728.99 | -0.0105815 |
| 727.993 | -0.0104838 |
| 726.996 | -0.0112491 |
| 725.998 | -0.0111522 |
| 725.001 | -0.0114814 |
| 724.003 | -0.0116451 |
| 723.004 | -0.0121774 |
| 722.006 | -0.01215 |
| 721.007 | -0.0124637 |
| 720.007 | -0.0125784 |
| 719.007 | -0.0124335 |
| 718.007 | -0.0125718 |
| 717.007 | -0.0125663 |
| 716.006 | -0.0123336 |
| 715.005 | -0.0120694 |
| 714.003 | -0.0119172 |
| 713.002 | -0.0114508 |
| 712 | -0.0110465 |
| 710.997 | -0.0106753 |
| 709.994 | -0.00986308 |
| 708.991 | -0.00941445 |
| 707.988 | -0.00878818 |
| 706.984 | -0.00804549 |
| 706.016 | -0.00730302 |
| 705.011 | -0.00677844 |
| 704.006 | -0.00592452 |
| 703.001 | -0.00507055 |
| 701.996 | -0.00455664 |
| 700.99 | -0.00350024 |
| 699.984 | -0.00323023 |
| 699.013 | -0.00225857 |
| 698.007 | -0.00168805 |
| 697 | -0.00101148 |
| 695.992 | -8.83e-05 |
| 694.984 | 0.000311908 |
| 694.012 | 0.000982292 |
| 693.004 | 0.00140547 |
| 691.995 | 0.00232006 |
| 690.986 | 0.00284008 |
| 690.013 | 0.00309276 |
| 689.003 | 0.00359962 |
| 687.993 | 0.00416568 |
| 686.983 | 0.00456242 |
| 686.008 | 0.0051129 |
| 684.997 | 0.00525813 |
| 683.986 | 0.00576621 |
| 683.01 | 0.00623061 |
| 681.998 | 0.00637693 |
| 680.986 | 0.00680942 |
| 680.01 | 0.00712779 |
| 678.997 | 0.00726275 |
| 677.984 | 0.00778664 |
| 677.006 | 0.00778867 |
| 675.992 | 0.00801957 |
| 675.015 | 0.00818778 |
| 674 | 0.0086454 |
| 672.985 | 0.00864385 |
| 672.007 | 0.00872582 |
| 670.991 | 0.00925712 |
| 670.012 | 0.00908706 |
| 668.996 | 0.00927028 |
| 668.016 | 0.00906005 |
| 666.999 | 0.0087521 |
| 665.982 | 0.00844231 |
| 665.001 | 0.00838617 |
| 663.984 | 0.00833841 |
| 663.002 | 0.00842602 |
| 661.984 | 0.00838559 |
| 661.002 | 0.00852332 |
| 659.983 | 0.00834144 |
| 659 | 0.00824516 |
| 658.017 | 0.00828814 |
| 656.997 | 0.00844708 |
| 656.014 | 0.00819807 |
| 654.993 | 0.00817285 |
| 654.009 | 0.00824732 |
| 652.988 | 0.00816544 |
| 652.004 | 0.00820715 |
| 650.982 | 0.00827212 |
| 649.997 | 0.00841839 |
| 649.011 | 0.00823479 |
| 647.989 | 0.00839968 |
| 647.002 | 0.00847694 |
| 646.016 | 0.00846469 |
| 644.993 | 0.00814687 |
| 644.006 | 0.00825606 |
| 643.018 | 0.00822508 |
| 641.994 | 0.00860181 |
| 641.006 | 0.00831455 |
| 640.018 | 0.00831286 |
| 638.993 | 0.00849998 |
| 638.004 | 0.00844099 |
| 637.015 | 0.00851455 |
| 635.989 | 0.00839206 |
| 634.999 | 0.00829616 |
| 634.009 | 0.0084071 |
| 632.983 | 0.00856474 |
| 631.992 | 0.00850291 |
| 631.001 | 0.00848792 |
| 630.01 | 0.00861604 |
| 628.982 | 0.00855595 |
| 627.991 | 0.00859618 |
| 626.999 | 0.00847139 |
| 626.007 | 0.00859798 |
| 625.014 | 0.00860957 |
| 623.985 | 0.0084824 |
| 622.992 | 0.00845471 |
| 621.999 | 0.00878094 |
| 621.005 | 0.0087591 |
| 620.011 | 0.00872373 |
| 619.017 | 0.0086548 |
| 617.986 | 0.00875413 |
| 616.991 | 0.00862053 |
| 615.996 | 0.00874623 |
| 615.001 | 0.00854602 |
| 614.006 | 0.00871771 |
| 613.01 | 0.00862692 |
| 612.014 | 0.00850341 |
| 611.018 | 0.00863426 |
| 609.985 | 0.00862594 |
| 608.988 | 0.0087176 |
| 607.991 | 0.00855537 |
| 606.993 | 0.00869745 |
| 605.996 | 0.00867992 |
| 604.998 | 0.00851366 |
| 604 | 0.00852316 |
| 603.001 | 0.00851854 |
| 602.003 | 0.00841694 |
| 601.004 | 0.00865179 |

**Figure S15.** UV-vis absorption spectrum for **5o** (2 mM in MeCN).

# 8. NMR Spectra

# 9. X-ray Crystallography

## Single Crystal Data for 5k (CCDC 2534670)

| ***Crystal data*** | |
| --- | --- |
| Identification code | DH_4_quin_iPr_BrNH_auto |
| Chemical formula | C_33_H_32_Cl_4_N_2_ |
| Formula weight (M_r_) | 598.40 |
| Temperature/K | 100 |
| Crystal system | triclinic |
| Space group | P-1 |
| a/Å | 9.88180(10) |
| b/Å | 12.0904(2) |
| c/Å | 15.1890(3) |
| α/° | 108.223(2) |
| β/° | 97.4190(10) |
| γ/° | 110.673(2) |
| Volume/Å^3^ | 1553.02(5) |
| Z | 2 |
| ρ_calc_g/cm^3^ | 1.280 |
| μ/mm^‑1^ | 3.644 |
| F(000) | 624.0 |
| Crystal size/mm^3^ | 0.245 × 0.11 × 0.086 |
| Radiation | Cu*K*α (λ = 1.54184) |
| ***Data Collection*** | |
| Diffractometer | Agilent SuperNova EosS2 diffractometer |
| Absorption correction | Multi-scan CrysAlis^PRO^ |
| Tmin,Tmax | 0.634,1.000 |
| 2Θ range for data collection/° | 8.406 to 143.124 |
| Index ranges | -12 ≤ h ≤ 12, -14 ≤ k ≤ 14, -18 ≤ l ≤ 18 |
| Reflections collected | 50622 |
| Independent reflections | 5983 [R_int_ = 0.0415, R_sigma_ = 0.0183] |
| ***Data refinement*** | |
| Data/restraints/parameters | 5983/0/355 |
| Goodness-of-fit on F^2^ | 1.071 |
| Final R indexes [I>=2σ (I)] | R_1_ = 0.0855, wR_2_ = 0.2658 |
| Final R indexes [all data] | R_1_ = 0.0914, wR_2_ = 0.2744 |
| Largest diff. peak/hole / e Å^-3^ | 1.38/-0.84 |

Computer programs: SuperNova, (Oxford Diffraction, 2010), CrysAlis PRO (Rigaku Oxford Diffraction, 2017), Olex2 (2009)

# 10. References

[1]	J. Cosier, A. M. Glazer, *Journal of Applied Crystallography* **1986**, *19*, 105-107.

[2]	Oxford Diffraction/Agilent Technologies UK Ltd, Yarnton, England.

[3]	G. M. Sheldrick, *Acta Crystallographica A* **2015**, *71*, 3-8.

[4]	G. Sheldrick, *Acta Crystallographica Section C* **2015**, *71*, 3-8.

[5]	O. V. Dolomanov, L. J. Bourhis, R. J. Gildea, J. A. K. Howard, H. Puschmann, *Journal of Applied Crystallography* **2009**, *42*, 339-341.

[6]	J. S. Sweet, S. Rajkumar, P. Dingwall, P. C. Knipe, *European Journal of Organic Chemistry* **2021**, *2021*, 3980-3985.

[7]	P. Zhang, C.-Q. Guo, W. Yao, C.-J. Lu, Y. Li, R. S. Paton, R.-R. Liu, *ACS Catalysis* **2023**, *13*, 7680-7690.

[8]	X. Pu, Y. Zhang, X. He, X. Zhang, L. Jiang, R. Cao, M. Kin Tse, L. Qiu, *Advanced Synthesis & Catalysis* **2023**, *365*, 1152-1157.

[9]	I. Takahashi, F. Morita, S. Kusagaya, H. Fukaya, O. Kitagawa, *Tetrahedron: Asymmetry* **2012**, *23*, 1657-1662.

[10]	H. Tinnermann, C. Wille, M. Alcarazo, *Angewandte Chemie International Edition* **2014**, *53*, 8732-8736.

[11]	J. S. Bandar, T. H. Lambert, *Journal of the American Chemical Society* **2012**, *134*, 5552-5555.

[12]	A. Arunachalampillai, P. Chandrappa, A. Cherney, R. Crockett, J. Doerfler, G. Johnson, V. C. Kommuri, A. Kyad, J. McManus, J. Murray, T. Myren, N. Fine Nathel, I. Ndukwe, A. Ortiz, M. Reed, H. Rui, M. V. Silva Elipe, J. Tedrow, S. Wells, S. Yacoob, K. Yamamoto, *Organic Letters* **2023**, *25*, 5856-5861.

[13]	J. J. Nuricumbo-Escobar, C. Campos-Alvarado, G. Ríos-Moreno, D. Morales-Morales, P. J. Walsh, M. Parra-Hake, *Inorganic Chemistry* **2007**, *46*, 6182-6189.

[14]	M. J. Frisch, G. W. Trucks, H. B. Schlegel, G. E. Scuseria, M. A. Robb, J. R. Cheeseman, G. Scalmani, V. Barone, G. A. Petersson, H. Nakatsuji, X. Li, M. Caricato, A. V. Marenich, J. Bloino, B. G. Janesko, R. Gomperts, B. Mennucci, H. P. Hratchian, J. V. Ortiz, A. F. Izmaylov, J. L. Sonnenberg, Williams, F. Ding, F. Lipparini, F. Egidi, J. Goings, B. Peng, A. Petrone, T. Henderson, D. Ranasinghe, V. G. Zakrzewski, J. Gao, N. Rega, G. Zheng, W. Liang, M. Hada, M. Ehara, K. Toyota, R. Fukuda, J. Hasegawa, M. Ishida, T. Nakajima, Y. Honda, O. Kitao, H. Nakai, T. Vreven, K. Throssell, J. A. Montgomery Jr., J. E. Peralta, F. Ogliaro, M. J. Bearpark, J. J. Heyd, E. N. Brothers, K. N. Kudin, V. N. Staroverov, T. A. Keith, R. Kobayashi, J. Normand, K. Raghavachari, A. P. Rendell, J. C. Burant, S. S. Iyengar, J. Tomasi, M. Cossi, J. M. Millam, M. Klene, C. Adamo, R. Cammi, J. W. Ochterski, R. L. Martin, K. Morokuma, O. Farkas, J. B. Foresman, D. J. Fox, Wallingford, CT, **2016**.
